# Synthesis of Selenylated Dibenzo[*b,f*]oxepines from an Electrophilic Cyclization Reaction

**DOI:** 10.1021/acsomega.6c07135

**Published:** 2026-07-29

**Authors:** Liliane S. de Farias, Sabrina B. Acosta, Luiz H. Dapper, Bruna R. Schneider, Angelita M. Barcellos, Liane K. Soares, Adriano B. de Oliveira, Diego Alves, Alex F. C. Flores, Gabriel P. da Costa

**Affiliations:** † Núcleo de Estudo Estrutural e Síntese de Heterociclos—NEESH, Universidade Federal do Rio Grande—FURG, Escola de Química e Alimentos—EQA, Itália Ave., km 8, no number—Campus Carreiros, Rio Grande, RS 96203-900, Brazil; ‡ Laboratório de Síntese Orgânica Limpa—LASOL, CCQFA, 37902 Universidade Federal de Pelotas—UFPel, P.O. Box 354, Pelotas, RS 96010-900, Brazil; § Pesquisa em Síntese Orgânica Sustentável—PSOS, Universidade Federal do Rio Grande—FURG, Escola de Química e Alimentos—EQA, Itália Ave., km 8, no number—Campus Carreiros, Rio Grande, RS 96203-900, Brazil; ∥ Núcleo de Química, Universidade Federal do Rio Grande, Escola de Química e Alimentos—EQA, Itália Ave., km 8, no number—Campus Carreiros, Rio Grande, RS 96203-900, Brazil

## Abstract

An efficient FeCl_3_-mediated intramolecular cyclization of *o*-alkynyl diaryl ethers with diorganyl diselenides has been developed, providing selenylated dibenzo­[*b,f*]­oxepines in good to excellent yields under mild conditions. The optimized protocol using 0.6 equiv of diorganyl diselenide and 1 equiv of FeCl_3_ in DCM at room temperature exhibited a broad substrate scope and high tolerance toward diverse functional groups. This approach occurs by *in situ* formation of an electrophilic selenium species, which undergoes a 7-*endo-dig* cyclization to furnish the heterocyclic core. This method provides straightforward access to functionalized dibenzo­[*b,f*]­oxepines containing selenium, a motif of interest due to its relevance in medicinal chemistry and materials science.

## Graphical abstract

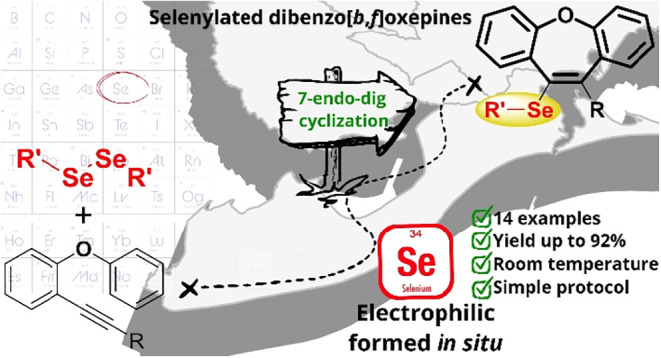

## Introduction

Oxygen heterocycles such as oxepines are valuable scaffolds in synthetic and medicinal chemistry.^1–3^ Among them, dibenzo­[*b,f*]­oxepines (DBOs) are particularly attractive^4–8^ due to their rigid polycyclic architecture and presence in bioactive compounds, including Omigapil (CGP 3466)^9^ and fluradoline,^10^ as well as natural products with anticancer and anti-inflammatory properties such as Artocarpol A,^11,12^ Bauhiniastatin 2,^13^ and Pacharin^14^ (Figure 1a).^1–8^ Several strategies have been developed for their construction,^1–8,15^ ranging from classical Friedel–Crafts and xanthene rearrangements to more recent Lewis acid-promoted 7-*endo-dig* cyclizations of *o*-phenoxy alkynes,^16–19^ which offer high efficiency and atom economy (Figure 1b).

**Figure 1:**
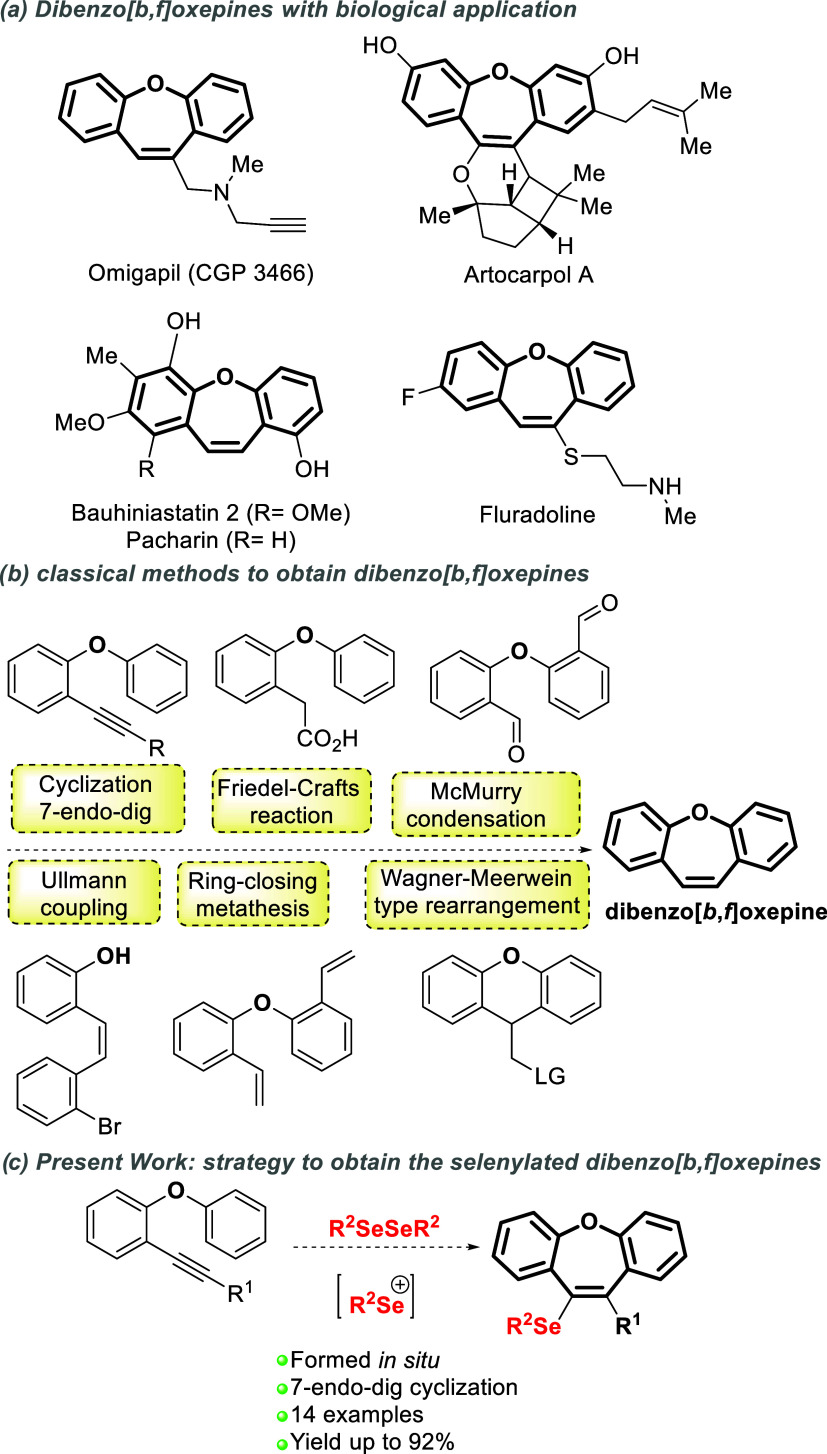
(a) Dibenzo­[*b*,*f*]­oxepines with biological applications. (b) Classical approaches to obtain dibenzo­[*b,f*]­oxepines. (c) Present work: strategy to obtain the selenylated dibenzo­[*b,f*]­oxepines **3**.

Organoselenium compounds have received remarkable attention due to their high reactivity and broad range of applications in organic synthesis^20,21^ and medicinal chemistry.^22–27^ Organoselenium reagents are powerful π-electrophiles broadly used in C–C and C–heteroatom bond formation, especially in oxidative alkyne functionalizations that yield diverse heterocycles.^28–30^ A wide spectrum of oxidants, including I_2_,^31,32^ Selectfluor,^33,34^ Oxone,^35,36^ trichloroisocyanuric acid (TCCA),^37–40^ FeCl_3_,^41–47^ CuI,^48^ and hypervalent iodine reagents,^49,50^ enables the formation of highly reactive electrophilic selenium species.

Despite significant progress in DBO synthesis and Se-promoted alkyne cyclizations, selenylated dibenzo­[*b,f*]­oxepines remain unknown. We now report the first FeCl_3_-mediated 7-*endo-dig* electrophilic selenocyclization of *o*-alkynyl diaryl ethers, providing direct access to *Se*-functionalized DBOs with a broad scope and high yields (Figure 1c).

## Results and Discussion

We began our studies by focusing on the synthesis of 10-phenyl-11-(phenylselanyl)­dibenzo­[*b,f*]­oxepine **3a**, obtained by reacting 1-phenoxy-2-(phenylethynyl)­benzene **1a** with diphenyl diselenide (**2a**), employing several protocols previously reported in the literature for the generation of electrophilic selenium species (Figure 2). The conditions described by Alves et al., in both protocols involving CuI,^48^ TCCA,^37^ were not efficient in synthesizing the target DBO **3a**. Similar results were obtained when potassium persulfate^51^ or a mixture of ^*m*^CPBA and KI^52^ was employed as an oxidative system (conditions reported by Zhao et al. and Yan et al.).

**Figure 2:**
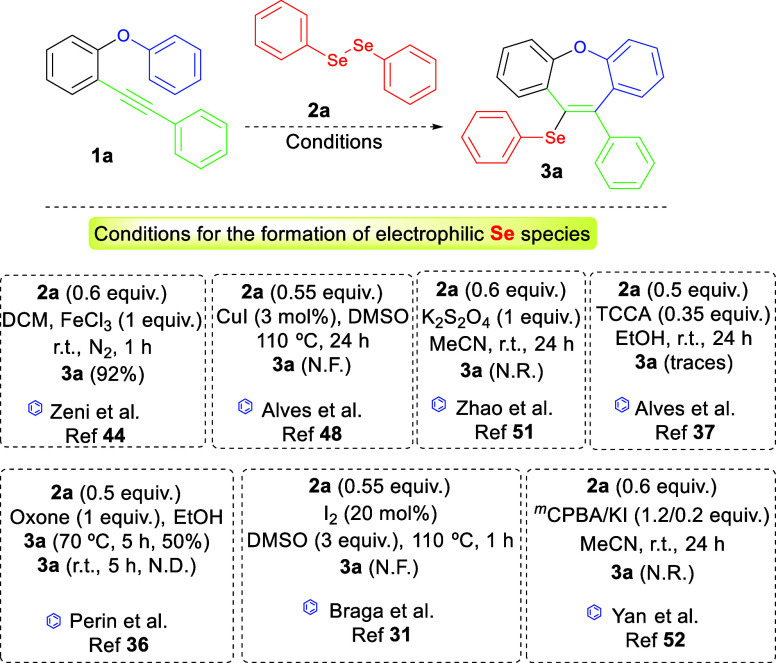
Evaluation of the reported protocol for obtaining selenylated dibenzo­[*b*,*f*]­oxepine **3a**.

The strategy reported by Braga et al. was also not efficient for obtaining the target seven-membered oxygen heterocycle (**3a**), where I_2_/DMSO^31^ was used as the catalytic system. The protocol reported by Perin et al. was also evaluated, in which Oxone was used as an oxidant.^36^ However, the compound (**3a**) was not formed when the reaction was performed at room temperature. Conversely, when the temperature was increased to 70 °C, the target DBO **3a** was obtained in 50% yield. In this case, some byproducts were also formed. Gratifyingly, the conditions previously reported by Zeni et al.^44^ proved to be efficient for this transformation, when the starting materials (**1a** and **2a**) were reacted in the presence of 1 equiv of FeCl_3_ at room temperature under a nitrogen atmosphere using DCM as the solvent for 1 h. Under these conditions, the target DBO **3a** was formed in excellent yield (92%) (Figure 2).

With these results in hand, we decided to optimize the FeCl_3_-mediated 7-*endo-dig* cyclization reaction of compound **1a** with **2a** to afford the target DBO (**3a**). Initially, we evaluated the amount of diphenyl diselenide **2a**, increasing its quantity from 0.6 equiv. to 1 equiv.; similar yields of **3a** were obtained (92% and 90%, respectively) (Table 1, entries 1–2). On the other hand, decreasing the amount of **2a** to 0.5 equiv. afforded the target DBO (**3a**) in a slightly lower yield (81%) (Table 1, entry 3). Based on these observations, 0.6 equiv of **2a** was selected as the optimal amount (Table 1, entry 1).

**Table 1:**
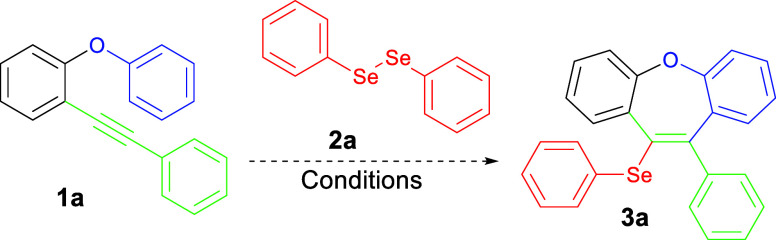
Optimization Conditions for the Synthesis of Selenylated Dibenzo­[*b*,*f*]­oxepine **3a**

entry^a^	**2a** (equiv)	solvent	cat. (equiv)	yield **3a** (%)^b^
1	0.6	DCM	FeCl_3_ (1)	92%
2	1.0	DCM	FeCl_3_ (1)	90%
3	0.5	DCM	FeCl_3_ (1)	81%
4	0.6	DCM	FeCl_3_ (0.5)	43%
5	0.6	DCE	FeCl_3_ (1)	60%
6	0.6	DCM	None	N.D.
7^c^	0.6	DCM	FeCl_3_ (1)	83^d^

a Reactions were carried out with starting material **1a** (0.125 mmol) and diphenyl diselenide **2a** (as indicated in the table) in solvent (1 mL). TLC followed the reaction progress.

b The yield of **3a** was determined after column chromatography.

c Reaction performed without a nitrogen atmosphere.

d Byproducts were formed in the reaction. N.D. not detected.

Subsequently, the influence of FeCl_3_ on the reaction was evaluated. When 0.5 equiv of FeCl_3_ was used instead of 1 equiv, the DBO **3a** yield decreased significantly (43%) (Table 1, entry 4), while when the reaction was performed in the absence of FeCl_3_, the DBO **3a** was not detected at all (Table 1, entry 5). On the basis of several reports using FeCl_3_ to promote cyclization reactions, some of which employ DCE as the solvent, we decided to replace DCM with DCE; however, the reaction was less efficient compared to that carried out in DCM (Table 1, entry 6).

The influence of the reaction atmosphere was evaluated. When the reaction was performed under an air atmosphere, compound **3a** was obtained in an 83% yield (Table 1, entry 7). Despite this good result, a small amount of side products was observed, which, due to their similar Rf values, made the purification process difficult. Therefore, we conducted subsequent experiments under a nitrogen atmosphere. It is important to note that no 6-exo-dig or chloroselenation products were obtained in sufficient quantities for complete isolation and characterization during the reaction optimization process.

The optimal conditions were established when compound **1a** was reacted with 0.6 equiv of diphenyl diselenide **2a** in the presence of 1 equiv of FeCl_3_, using DCM as the solvent, at room temperature for 1 h under nitrogen (Table 1, entry 1). With these optimized conditions in hand, we proceeded to evaluate the scope and limitations of the protocol. When the reaction was carried out on a 1 mmol scale to evaluate its scalability and robustness, the desired product **3a** was obtained in an isolated yield of 81%, demonstrating that the efficiency of the transformation is well-maintained upon scale-up. This result highlights the practical applicability of the developed protocol under slightly larger scale conditions without a significant loss in yield (Figure 3).

**Figure 3:**
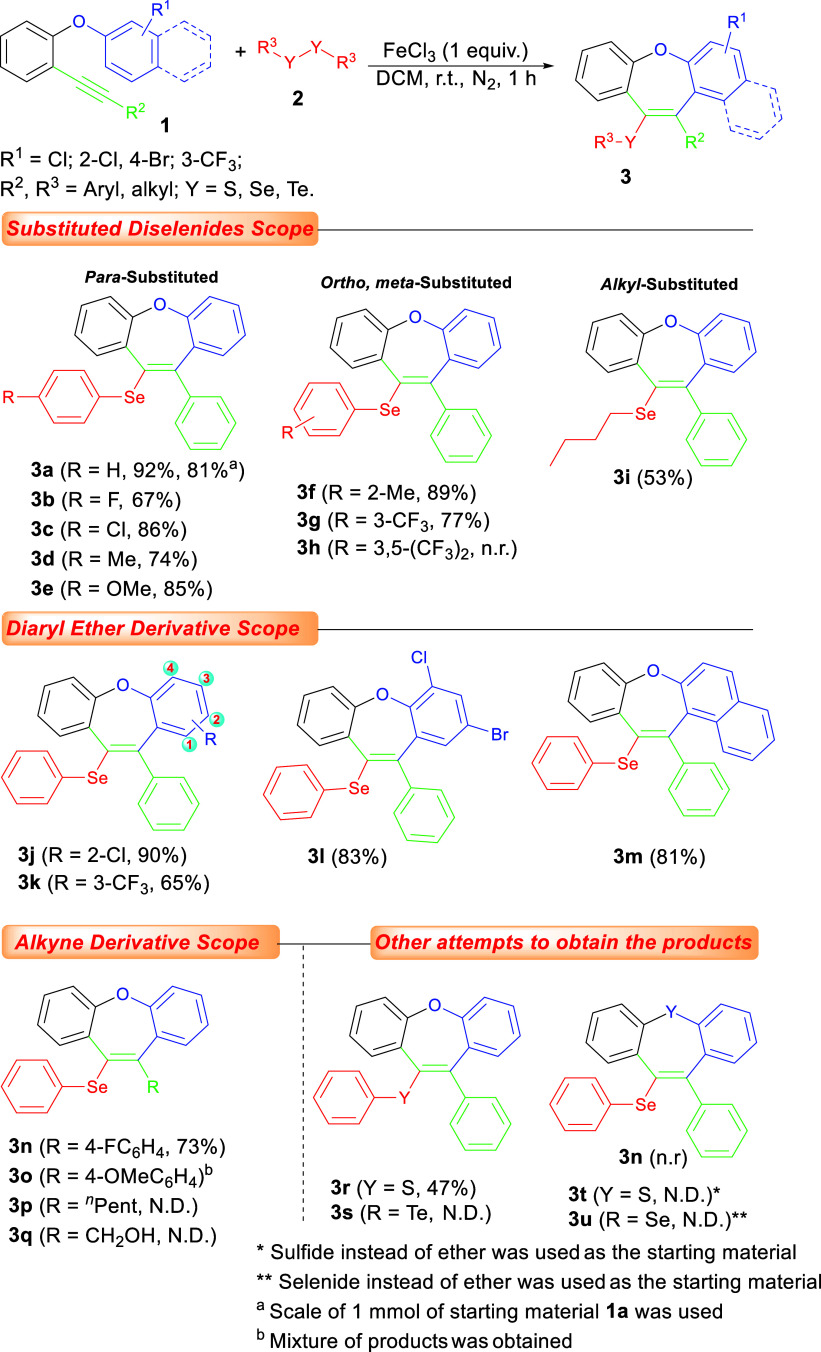
Evaluation of versatility and limitation of scope for the synthesis of substituted selenylated-dibenzo­[*b,f*]­oxepines.**3**, reactions were carried out with starting material **1** (0.125 mmol), diorganyl dichacogenide **2** (0.075 mmol, 0.6 equiv) in DCM (1 mL) at room temperature under nitrogen atmosphere for 1 h. N.D.: not detected.

Initially, we began our investigation by examining the influence of substituents attached to the aromatic ring of diaryl diselenides **2**, including both electron-donating and electron-withdrawing groups located at the *para* position of the aromatic ring. The target DBO-selenylated compounds **3b**–**e** were synthesized in yields ranging from moderate to good (67–86%) (Figure 3). Similar results were obtained when *ortho*-methyl- or *meta*-trifluoromethyl-substituted diselenides were evaluated, affording compounds **3f** and **3g** in 89 and 77% yield, respectively. Unfortunately, this protocol proved to be restricted when 1,2-bis­(3,5-bis­(trifluoromethyl)­phenyl)­diselenide was used as the starting material; in this case, the target product **3h** was not formed. This methodology also proved applicable to dialkyl diselenides, and when dibutyl diselenide was reacted under the standard conditions, the target DBO-selenylated compound **3i** was obtained in 53% yield (Figure 3).

Subsequently, we focused on evaluating the substituent effects on the *o*-alkynyl diaryl ether scaffold **1**. The phenol-substituted derivatives were examined, and in these cases, the monosubstituted compounds **3j** and **3k** were synthesized in 90 and 65% yield, respectively. The dihalogen-substituted DBO-selenylated derivative **3l** was obtained in good yield (83%), and a similar result was observed for the naphthyl derivative, which afforded the target product **3m** in 81% yield. The influence of substituents attached to the alkynyl unit was also evaluated. The presence of a fluorine atom at the *para*-position of the aromatic ring of **1** led to the target cyclization product **3n** in good yield (73%). In contrast, when the 4-OMe-substituted starting material **1** was used, the reaction produced the target product **3o** along with side products in an inseparable mixture. Unfortunately, when alkyl substituents such as *n*-pentyl or −CH_2_OH were present, the reaction did not proceed (compounds **3p** and **3q**, Figure 3).

Additionally, other diphenyl dichalcogenides were also evaluated, and when diphenyl disulfide was used as the starting material, the target sulfenylated DBO **3r** was formed in moderate yield (47%). Conversely, diphenyl ditelluride proved ineffective under the standard conditions, and no traces of the target product **3s** were detected. Furthermore, we changed the heteroatom of the starting material from the diaryl ether to the corresponding diaryl sulfide or diaryl selenide. In both cases, the target S- or Se-containing heterocyclic product **3t** or **3u** was not obtained, and the starting materials were largely recovered unchanged (90–95%) (Figure 3).

A series of optimized control experiments provides key evidence to support the mechanistic proposal. When TCCA or Oxone was employed as the external oxidant in EtOH, the product corresponded to selenoethoxylated and chlorinated derivatives being formed, strongly suggesting the formation of a seleniranium ion as a key intermediate under oxidative conditions (Figure 2). Consistently, the reaction carried out with PhSeCl **4a** predominantly furnished the desired cyclized product **3a**, albeit accompanied by side products (Scheme 1, eq 1). This observation indicates that PhSeCl is a competent selenium electrophile in the transformation but likely not the sole reactive species operating in the catalytic system.^41–47^

**Scheme 1:**
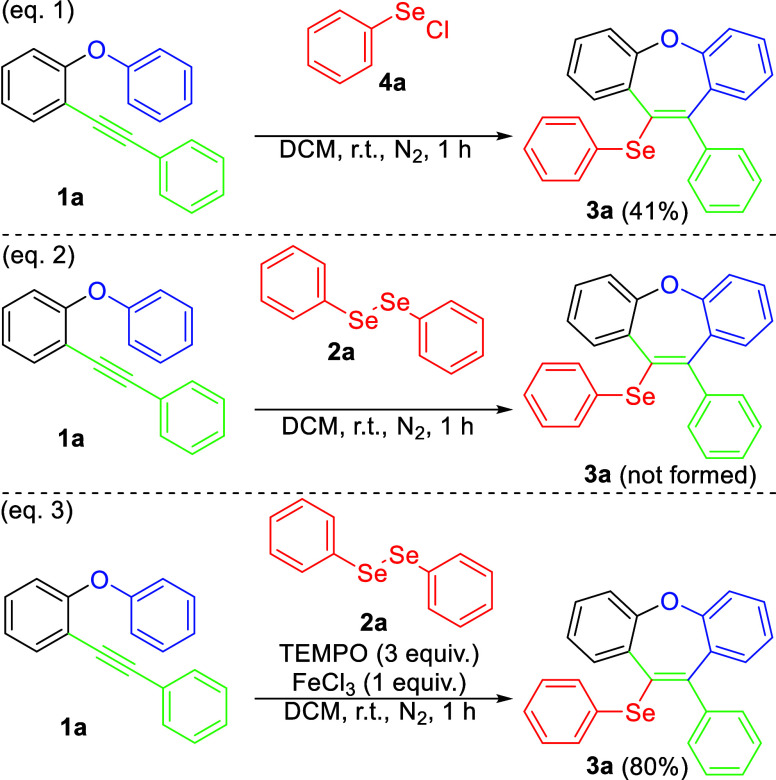
Mechanistic Studies to Synthesize the Selenylated Dibenzo­[*b,f*]­oxepine **3**

Additionally, the reaction in the absence of diphenyl diselenide **2a** gives the cyclic dibenzo­[*b,f*]­oxepine, as previously reported.^16^ Moreover, no conversion was observed in the absence of FeCl_3_ (Scheme 1, eq 2), in agreement with previous studies,^41–47^ which demonstrated the essential role of FeCl_3_ in promoting the activation of diorganyl diselenides **2** and enabling the selenocyclization manifold. The reaction carried out in the presence of TEMPO under the optimized conditions afforded the desired product **3a** in 80% yield, indicating that a radical pathway is unlikely involved in the transformation (Scheme 1, eq 3). Altogether, these results support a mechanistic scenario involving (i) FeCl_3_-mediated oxidation of the diselenide to generate electrophilic selenium species (including, but not limited to, PhSeCl), (ii) formation of a seleniranium intermediate upon addition to the alkyne, and (iii) subsequent intramolecular nucleophilic attack leading to the observed heterocycle. These data also suggest an alternative pathway in which the alkyne is activated directly by an electrophilic iron–selenium species generated *in situ*.

Based on these mechanistic observations and on well-established precedents reported by Zeni et al.,^41–47^ a plausible dual-pathway mechanism is proposed (Scheme 2). Two reaction pathways can be envisioned. The first pathway begins with the formation of the RSeCl species **I** through the reaction of diorganyl diselenide **2** with FeCl_3_. Subsequently, **I** reacts with the alkyne moiety of starting material **1**, generating the seleniranium ion **II**, which is intramolecularly attacked by the aromatic ring, assisted by anchimeric participation of the phenolic oxygen, to afford intermediate **III** via a 7-*endo-dig* cyclization. Next, rearomatization of **III** occurs through the elimination of a hydrogen atom, affording the target selenylated dibenzo­[*b,f*]­oxepine **3** (pathway A).

**Scheme 2:**
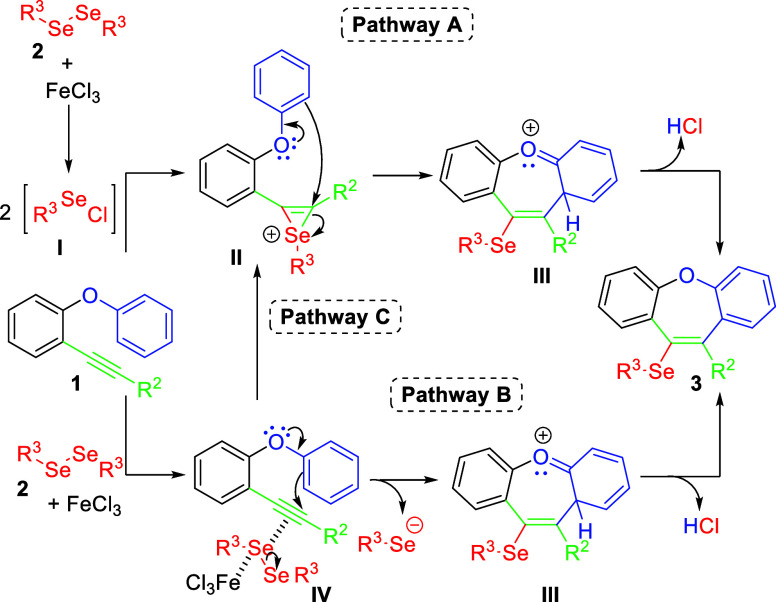
Proposed Mechanism to Synthesize the Selenylated Dibenzo­[*b,f*]­oxepine **3**

In the second pathway, the selenocyclization leading to the dibenzo­[*b,f*]­oxepine **3** framework may instead be initiated with activation of the alkyne by an electrophilic iron–selenium complex **IV**, generated *in situ* from FeCl_3_ and the diselenide. The phenolic oxygen stabilizes the developing cationic character and facilitates an intramolecular nucleophilic attack of the aromatic carbon onto the activated alkyne-derived center, triggering ring closure to provide the seven-membered oxepine intermediate **III** while incorporating the organoselenium moiety into the polycyclic structure. Finally, rearomatization of **III** furnishes the target product **3** (pathway B, Scheme 2). In addition, a third pathway (pathway C) cannot be ruled out. In this scenario, the electrophilic iron–selenium complex **IV** may serve as a precursor to the seleniranium ion **II** (pathway C). The resulting species could then participate in the cyclization sequence described in pathway A, suggesting that both mechanistic manifolds may operate simultaneously under the reaction conditions and converge toward the formation of product **3** (Scheme 2).

To further demonstrate the synthetic utility of the selenylated dibenzo­[*b,f*]­oxepine scaffold, compound **3a** was selected as a representative substrate for postfunctionalization studies. Initially, the oxidation of the organoselenium moiety was investigated using *m*-chloroperbenzoic acid (^*m*^CPBA), a well-established oxidant for the selective transformation of selenides into the corresponding oxidized selenium derivatives. Under these conditions, the compound **3a** underwent a clean oxidation process, affording product **5** in satisfactory yield (89%) (Scheme 2, eq 1). This result highlights the potential of the selenium-containing dibenzo­[*b,f*]­oxepine framework as a versatile synthetic intermediate, enabling further structural diversification through straightforward oxidative transformations. The use of *N*-bromosuccinimide (NBS) proved effective for this transformation, leading to the formation of product **6** in 81% isolated yield (Scheme 2, eq 1). These results further highlight the synthetic versatility of the selenium-containing dibenzo­[*b,f*]­oxepine scaffold. In addition to the oxidation reaction described above, treatment of compound **3a** with ^*n*^BuLi at −78 °C followed by quenching with water as an electrophile led to the formation of product **7** in 73% of conversion, which was detected by GC–MS analysis (Scheme 3, eq 3). These transformations demonstrate the potential of these organoselenium dibenzo­[*b,f*]­oxepines as useful synthetic intermediates for further functionalization.

**Scheme 3:**
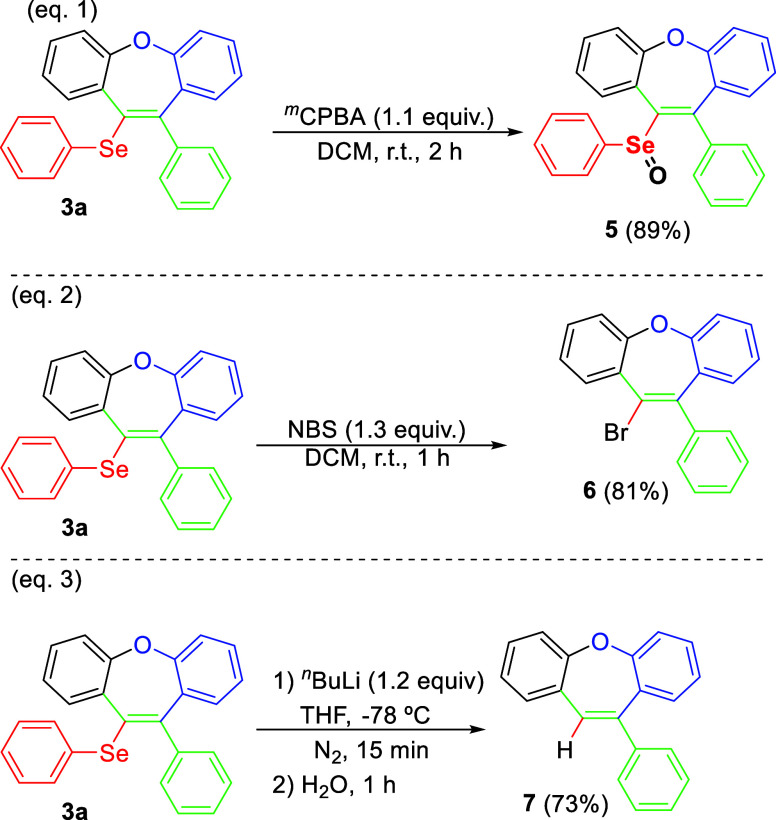
Synthetic Utility of the Selenylated Dibenzo­[*b,f*]­oxepine **3** Scaffold

To corroborate with the mechanistic study and the spectroscopic analyses of the selenylated dibenzo­[*b,f*]­oxepine derivatives presented in this work, the molecular structure of **3a** was determined using the single-crystal X-ray diffraction technique (SC-XRD). Therefore, clear light orange single crystals suitable for the analysis of the 10-phenyl-11-(phenylselanyl)­dibenzo­[*b,f*]­oxepine derivative were obtained from a solution in acetone by the slow evaporation of the solvent, at room temperature, within a week. The data collection was performed with a Rigaku XtaLAB Synergy-S diffractometer equipped with a Hypix area detector and a nitrogen-based cryostream open-flow cooler device (Oxford Cryosystems Cryostream 800 PLUS) at 123 K, using monochromatized Cu-*K*α radiation (λ = 1.54184 Å). The data collection, cell refinement, data reduction, and absorption correction were performed with the *CrisAlisPro* software package, ver. 1.171.44.149a.^53^ The crystal system and the noncentrosymmetric space group were identified as monoclinic and *P*12_1_1, which was confirmed by the Flack parameter of 0.070(8).^54–57^ The crystal structure was solved with *SHELXT*-2015 ^58,59^ and refined anisotropically using the full-matrix least-squares technique on *F* ^2^ for all non-hydrogen atoms with *SHELXL*-2019/3.^60,61^ The H atoms were positioned with idealized geometry and refined isotropically using a riding model (HFIX instruction). All hydrogen atoms were attached to *sp* ^2^ hybridized carbon atoms, with *U*_iso_(H) = 1.2 *U*_eq_(C), and the C–H bond lengths were set to 0.9300 Å. The molecular graphic was generated with the *DIAMOND* software, ver. 3.2e,^62^ and the material for publication was prepared with the *WinGX*, ver. 2023.1,^63^ *enCIFer*, ver. 2026.1.0,^64^ and *publCIF*, ver. 1.9.22_c,^65^ software. The database survey was performed with the *ConQuest* software, ver. 2025.2.0,^66^ and the Cambridge Structural Database via WebCSD, ver. 1.9.85.^67^ As a result of the SC-XRD analysis, the asymmetric unit of **3a** matches the molecular formula, with all atoms being located in general positions (Figure 4). The molecule is not planar due to the oxygen atom in the seven-membered ring, with the C1–O1–C26 bond angle of 111.5(2)°, and the observed dihedral angle between the mean planes through the C1/C2/C3/C4/C5/C6 and C21/C22/C23/C24/C25/C26 entities of 60.61(11)°. In addition, due to the free rotation of the peripheral aromatic rings around the internuclear axis of the C7–C8 and Se1–C15 single bonds, the dihedral angle between the C8/C9/C10/C11/C12/C13 and C15/C16/C17/C18/C19/C20 moieties amounts to 58.39(08)°, which contributes to the three-dimensional molecular geometry and the point group of *C*_1_ of this selenylated dibenzo­[*b,f*]­oxepine derivative. Crystal data, data collection, and structure refinement details for the **3a** analysis are summarized in Table 2.

**Figure 4:**
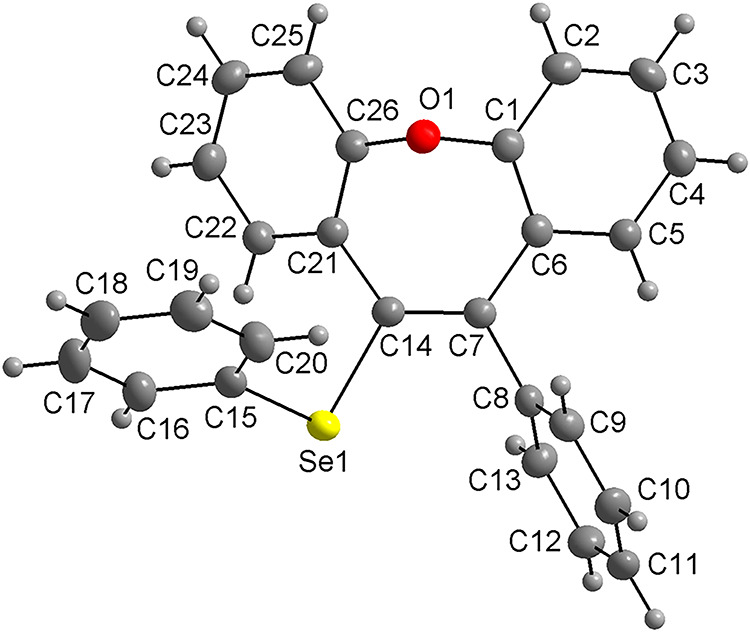
Molecular structure of **3a**, showing the atom labeling and displacement ellipsoids drawn at the 50% probability level for the non-H atoms.

**Table 2:** Crystal Data, Data Collection, and Structure Refinement Details for **3a**

chemical formula	C_26_H_18_OSe
*M*_r_	425.36
crystal system	monoclinic
space group (IT)	4
space group (H-M)	*P*2_1_ or *P*12_1_1
space group (hall symbol)	*P* 2yb
temperature (K)	123
*a*, *b*, *c* (Å)	9.0551 (2), 8.6134 (2), 12.9251 (3)
β (°)	105.340 (2)
*V* (Å^3^)	972.18 (4)
*Z*	2
radiation type	Cu-*K*α
μ (mm^–1^)	2.71
crystal size (mm)	0.24 × 0.13 × 0.07
diffractometer	Rigaku XtaLAB Synergy-S, Dualflex, Hypix area
	detector diffractometer
absorption correction	Gaussian [using the SCALE3 ABSPACK scaling algorithm embedded within the *CrysAlisPro* software package (^53^)]
*T*_min_, *T*_max_	0.495, 1.000
no. of measured, independent, and Observed [*I* > 2σ(*I*)] reflections	21,119, 4127, 4077
*R*_int_	0.034
(sin θ/λ)_max_ (Å^–1^)	0.638
*R*[*F* ^2^ > 2σ(*F* ^2^)], *w*R** (*F* ^2^), *S*	0.024, 0.062, 1.08
no. of reflections	4127
no. of parameters	253
no. of restraints	1
H atom treatment	H atom parameters constrained
Δρ_max_, Δρ_min_ (e Å^–3^)	0.22, −0.51
absolute structure	flack *x* determined using 1826 quotients [(I+)–(I−)]/[(I+)+(I−)] (^54^)
absolute structure parameter	0.070 (8)

To the best of our knowledge and from using database tools such as the *ConQuest* software, ver. 2025.2.0,^66^ (accessed on June 05, 2026) and the Cambridge Structural Database, ver. 1.9.85, (CSD, accessed *via WebCSD* on June 05, 2026),^67^ the crystal structure of **3a** is reported in the literature for the first time in this work.

## Conclusions

In summary, we have successfully developed an efficient FeCl_3_-mediated intramolecular cyclization of *o*-alkynyl diaryl ethers with diorganyl diselenides to afford the corresponding selenylated dibenzo­[*b,f*]­oxepines in good to excellent yields under mild conditions. The optimized protocol, employing 0.6 equiv of diselenide and 1 equiv of FeCl_3_ in DCM at room temperature under a nitrogen atmosphere, demonstrated a broad substrate scope and high functional group tolerance. Mechanistic investigations suggest that the transformation proceeds through the *in situ* generation of an electrophilic selenium species, followed by a 7-*endo-dig* cyclization pathway leading to the selenylated heterocycle. Despite these promising results, attempts to extend the methodology to diaryl sulfides and selenides did not afford the desired heterocyclic products, indicating a strong influence of the heteroatom on the cyclization outcome. Further studies are currently underway in our laboratory to expand the synthetic applicability of this methodology, including the use of disulfides and ditellurides as chalcogen sources, as well as to gain deeper mechanistic insights into the reaction pathway.

## Supplementary Material



## Notes

Copy of ^1^H, ^13^C­{^1^H}, and ^77^Se NMR spectra as well as spectral data of all compounds and the procedures; geometric parameters (Å, °) for **3a** from the SC-XRD analysis (PDF)
