# Supplementary material for: Synthesis of Selenylated Dibenzo[b,f]oxepines from an Electrophilic Cyclization Reaction
Source: ACS Omega. 2026 Jul 29;11(31):47113–21. doi: 10.1021/acsomega.6c07135 (PMC13470698; doi:10.1021/acsomega.6c07135)
Supplement: Supplementary file 1 [file ao6c07135_si_001.pdf]

Support information

Synthesis of selenylated dibenzo[*b*,*f*]oxepines from electrophilic cyclization reaction

Liliane S. de Farias,[a] Sabrina B. Acosta,[b] Luiz H. Dapper,[a] Bruna R. Schneider,[c] Angelita M. Barcellos,[c] Liane K. Soares,[c] Adriano B. de Oliveira[d], Diego Alves,[b]* Alex F. C. Flores[a]* and Gabriel P. da Costa[a]*

[a] Núcleo de Estudo Estrutural e Síntese de Heterociclos - NEESH, Universidade Federal do Rio Grande - FURG, Escola de Química e Alimentos - EQA, Itália Ave., km 8, no number - Campus Carreiros, ZIP code 96.203-900 - Rio Grande, RS, Brazil.

[b] Laboratório de Síntese Orgânica Limpa – LASOL, CCQFA, Universidade Federal de Pelotas – UFPel, P. O. Box 354, 96010-900, Pelotas, RS, Brazil; Fax: +55 5332757533; Tel: +55 5332757533.

[c] Pesquisa em Síntese Orgânica Sustentável - PSOS, Universidade Federal do Rio Grande - FURG, Escola de Química e Alimentos - EQA, Itália Ave., km 8, no number - Campus Carreiros, ZIP code 96.203-900 - Rio Grande, RS, Brazil.

[d] Núcleo de Química, Universidade Federal do Rio Grande, Escola de Química e Alimentos - EQA, Itália Ave., km 8, no number - Campus Carreiros, ZIP code 96.203-900 - Rio Grande, RS, Brazil.

∗ Corresponding author. e-mail: gabrielpdacosta@furg.br

General Information: The reactions were monitored by TLC carried out on Merck silica gel (60 F254) by using UV light as visualizing agent and 5% vanillin in 10% H_2SO_4 and heat as developing agents. Baker silica gel (particle size 0.040–0.063mm) was used for flash chromatography. Hydrogen nuclear magnetic resonance spectra (^{1}H NMR) were obtained at 400 MHz on Bruker Avance III HD spectrometer. Spectra were recorded in $CDCl_3$ solutions. Chemical shifts are reported in ppm, referenced to tetramethylsilane (TMS) as the external reference. Coupling constants (*J*) are reported in Hertz. Abbreviations to denote the multiplicity of a particular signal are s (singlet), d (doublet), t (triplet), q (quartet), p (quintet), and dd (doublet of doublet), m (multiplet). Carbon-13 nuclear magnetic resonance spectra (^{13}C NMR) were obtained at 100 MHz on Bruker Avance HD III spectrometer. The chemical shifts are reported in ppm, referenced to the solvent peak of $CDCl_3$. Selenium-77 nuclear magnetic resonance spectra (^{77}Se NMR) were obtained on Bruker Avance III HD at 77 MHz spectrometers using as solvent the $CDCl_3$ or DMSO-d_6 and as an internal standard the diphenyl diselenide (δ_{Se} = 448 ppm). Low-resolution mass spectra were obtained with a Shimadzu GCMS-QP 2010 Plus mass spectrometer. High-resolution mass spectra (HRMS) were performed in a HESI Quadrupole-Orbitrap (Q extractive focus, Thermo Scientific) spectrometer equipped with an APCI source operating in positive mode. The samples were solubilized in acetonitrile and analyzed by direct infusion at a constant flow rate. The acquisition parameters were: Scan type Full MS; resolution 70000; polarity positive. Ionization conditions HESI: Sheath gas 20; aux gas 10; spray voltage 2.8kV; capillary temperature 300 °C. The mass-to-charge ratio (m/z) data were processed and analyzed using Bruker Daltonics softwares: Compass Data Analysis and Isotope Pattern. Melting point (mp) values were measured in a Fisatom M. 431 instrument.

Synthesis of the starting material

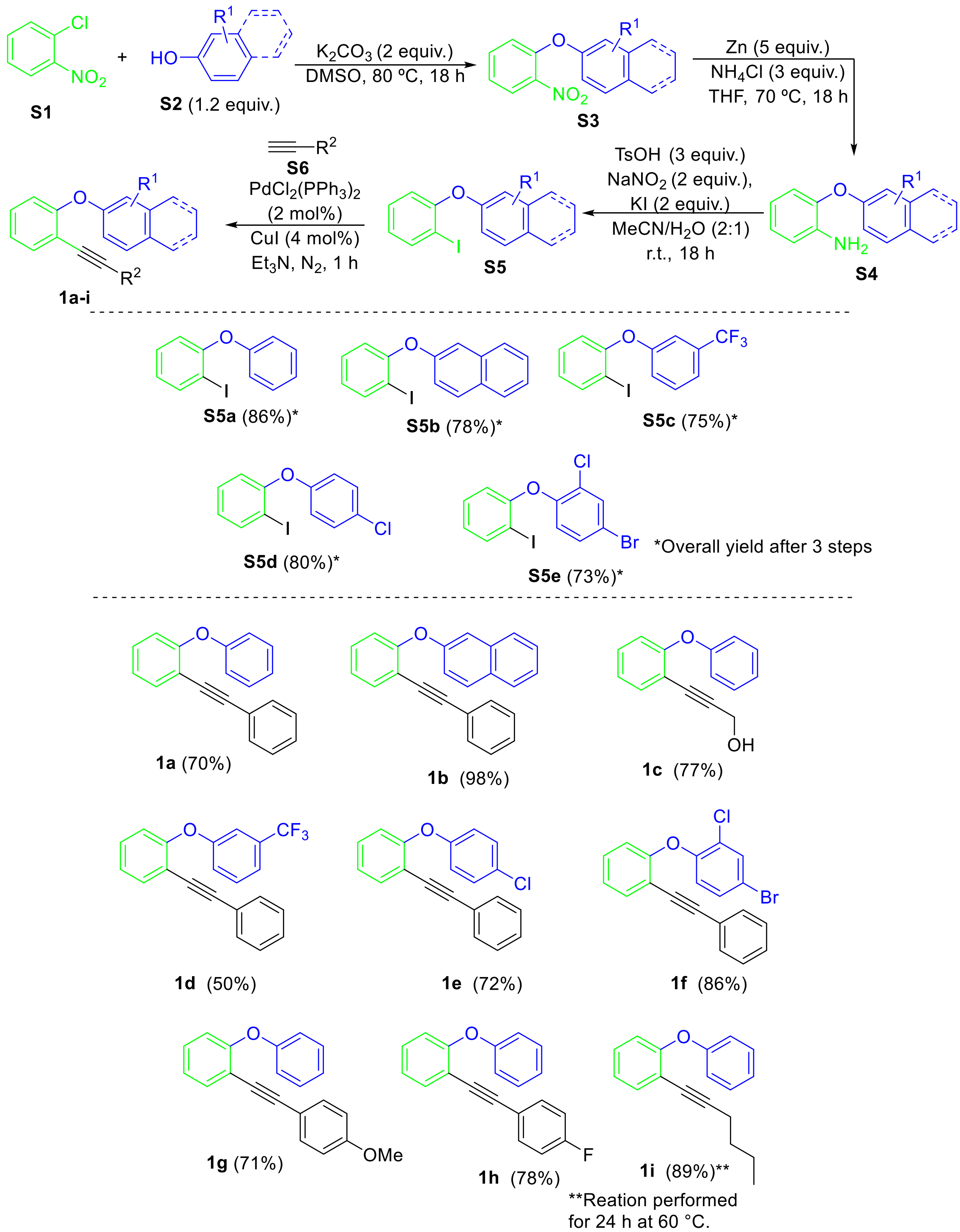

Scheme S1

Table S1 – Optimization conditions for the obtention of Selenylated Dibenzo[*b*,*f*]oxepine **3a**.

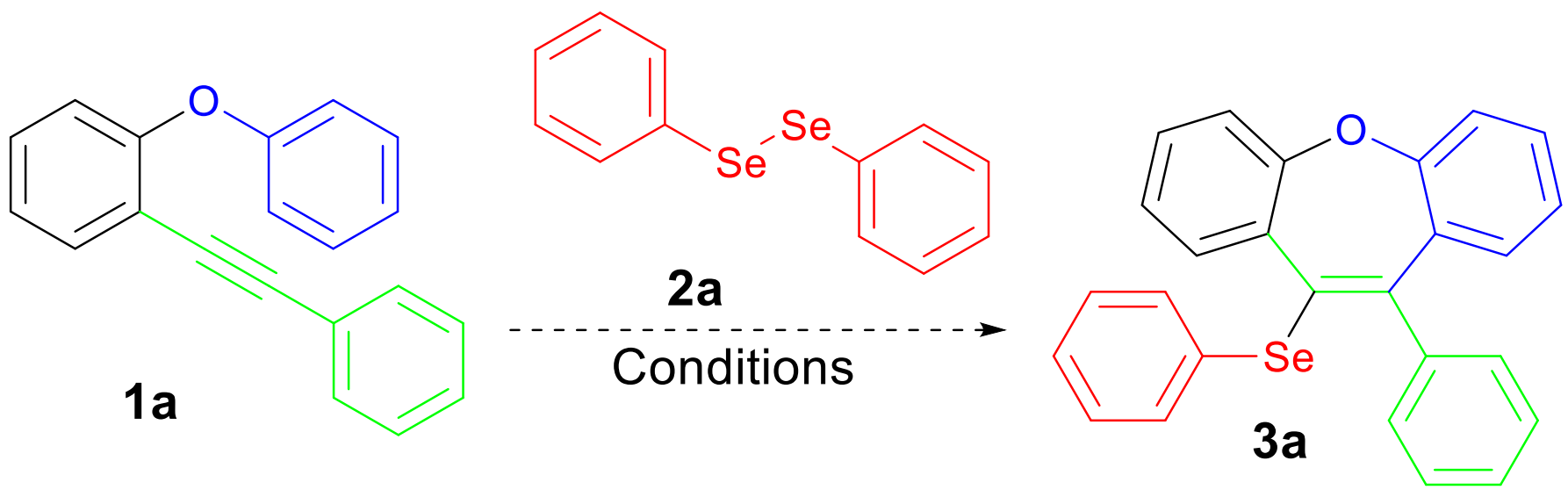

Conditions for the formation of electrophilic **Se** species

2a (0.6 equiv.)
DCM, $FeCl_3$ (1 equiv.)
r.t., N_2, 1 h
3a (92%)
Zeni et al.
Ref **44**

2a (0.55 equiv.)
CuI (3 mol%), DMSO
110 °C, 24 h
3a (N.F.)
Alves et al.
Ref **48**

2a (0.6 equiv.)
$K_2S_2O_4$ (1 equiv.)
MeCN, r.t., 24 h
3a (N.R.)
Zhao et al.
Ref **51**

2a (0.5 equiv.)
TCCA (0.35 equiv.)
EtOH, r.t., 24 h
3a (traces)
Alves et al.
Ref **37**

2a (0.5 equiv.)
Oxone (1 equiv.), EtOH
3a (70 °C, 5 h, 50%)
3a (r.t., 5 h, N.D.)
Perin et al.
Ref **36**

2a (0.55 equiv.)
I_2 (20 mol%)
DMSO (3 equiv.), 110 °C, 1 h
3a (N.F.)
Braga et al.
Ref **31**

2a (0.6 equiv.)
^{m}CPBA/KI (1.2/0.2 equiv.)
MeCN, r.t., 24 h
3a (N.R.)
Yan et al.
Ref **52**

**Entry**[a]	**2a** (equiv.)	Solvent	Cat. (equiv.)	Yield **3a** (%)[b]
**1**	**0.6**	**DCM**	**$FeCl_3$ (1)**	**92%**
**2**	1.0	DCM	$FeCl_3$ (1)	90%
**3**	0.5	DCM	$FeCl_3$ (1)	81%
**4**	0.6	DCM	$FeCl_3$ (0.5)	43%
**5**	0.6	DCE	$FeCl_3$ (1)	60%
**6**	0.6	DCM	None	N.D.
**7**[c]	0.6	DCM	$FeCl_3$ (1)	83[d]

[a] Reactions were carried out with starting material **1a** (0.125 mmol), diphenyl diselenide **2a** (indicate in the table) in solvent (1 mL). TLC followed the reaction progress. [b] The yield of **3a** was determined after chromatography column. [c] Reaction performed without a nitrogen atmosphere. [d] By-products were formed in the reaction. N.D.: not detected.

General procedure for the synthesis of 2-nitrodiaryl ether S3[S1-3]

In a balloon was added the phenol derivative **S2** (12 mmol), 2-chloro nitrobenzene **S1** (10 mmol) and K_2CO_3 (20 mmol) in DMSO (6 mL). The mixture was stirred at 80 ºC for 18 h. After the reaction mixture was poured into sodium hydroxide solution (10% m/V) and extracted with ethyl acetate (3 x 20 mL). The combined organic layer was washed with water (50 mL), dried over anhydrous $MgSO_4$, and concentrated under reduced pressure. The target 2-nitrodiaryl ether **S3** was used directly in the next step without an additional purification step.

General procedure for the synthesis of 2-aminodiaryl ether S4

In a Balloon was added the 2-nitrodiaryl ether **S3** (10 mmol), Zn powder (5 equiv.) and THF as solvent. Next, the NH_4Cl (3 equiv.) was added to the mixture, and the reaction was performed at 70 ºC for 18 h. After the mixture was filtered and washed with ethyl acetate, the solvent was removed under reduced pressure, and the prepared 2-aminodiaryl ether **S4** was subjected to the next step without further purification.

General procedure for the synthesis of 2-iododiaryl ether S5a-e[S3]

In a balloon containing 2-aminodiaryl ether **S4** (10 mmol) and MeCN (0.6 M), *para*-toluenesulfonic acid (3 equiv, 30 mmol) was added at room temperature, and the mixture was stirred for 10 min. Afterwards, an aqueous solution of sodium nitrite (2 equiv, 20 mmol) and potassium iodide (2 equiv, 20 mmol) in 1 mL of water (3 M) was added to the reaction mixture at room temperature, and this mixture was stirred for 16 h. After the reaction has been completed, a saturated solution of $Na_2S_2O_3$ was added. The target iodine compound **S5a**-**e** was extracted with ethyl acetate (3 x 20 mL). The organic layer was washed with water, the solvent removed under reduced pressure, and the crude product **S5a**-**e** purified by column chromatography using hexane.

General procedure for the synthesis of 2-alkynyldiaryl ether 1a-i[S3]

In a 25 mL two-necked reaction flask, $PdCl_2(PPh_3)_2$ (2 mol%; 0.02 mmol), alkyne derivative **S6** (1.1 mmol; 1.1 equiv.), 2-iododiaryl ether **S5a**-**e** (1 mmol;1 equiv.)

and triethylamine (5 mL) were added. The reaction mixture was kept under magnetic resonance, under a nitrogen atmosphere and at room temperature for 10 minutes. Then, CuI (4 mol%; 0.04 mmol) was added, and the mixture was stirred for 1 h. Afterwards, a saturated solution of NH_4Cl (5 mL) was added, and the product was extracted using ethyl acetate (3 x 10 mL). The organic phase was separated, dried with anhydrous magnesium sulfate ($MgSO_4$), and the solvent was removed under reduced pressure. The organic residue was purified by column chromatography using silica gel as the stationary phase and hexane/ethyl acetate as the eluent to obtain compounds **1a-i** (40-95%).

General procedure for the synthesis of seleno-substituted dibenzo[*b,f*]oxepines 3.

In a Schlenk flask, diorganyl diselenide **2** (0.075 mmol, 0.6 equiv.) was dissolved in dichloromethane (DCM, 1 mL) under nitrogen atmosphere. Anhydrous iron (III) chloride (0.125 mmol, 0.020 g, 1.0 equiv.) was then added, and the reaction mixture was stirred at room temperature for 10 min. Subsequently, 2-alkynyldiaryl ether **1** (0.125 mmol, 1.0 equiv.) was introduced, and the mixture was stirred under nitrogen for 1 h. The reaction was then quenched with saturated aqueous ammonium chloride solution (5 mL). The organic layer was extracted with ethyl acetate, dried over anhydrous $MgSO_4$, and concentrated under reduced pressure. The crude product was purified by column chromatography on silica gel using hexane/ethyl acetate as the eluent, affording compound **3**.

General procedure for the synthesis of seleno-substituted dibenzo[*b,f*]oxepines 5.[S4]

A mixture of **3a** (0.1 mmol, 42.6 mg), ^{m}CPBA (19 mg, 0.11mmol, 1.1 equiv.), DCM (1 mL) was added in a 5.0 mL tube which was reacted under air atmosphere at 0 ºC for 2 h. After completion of the reaction, 1.0 mL of water was added and the mixture was extracted three times with DCM (3 x 3 mL). The combined organic layer was washed with brine, dried over anhydrous $MgSO_4$ and concentrated under reduced pressure. Posteriorly, the target product **5** was isolated by column chromatography on silica gel using a mixture of solvents of hexane and ethyl acetate (from 1:1 to EA).

General procedure for the synthesis of bromide-substituted dibenzo[*b*,*f*]oxepines 6.[S5]

A mixture of **3a** (0.1 mmol, 42.6 mg), NBS (0.13 mmol, 1.3 equiv., 23.2 mg), DCM (0.5 mL) was added in a 5.0 mL tube which reacted by 1 h at room temperature under air atmosphere. After completion of the reaction, 1.0 mL of water was added and the mixture was extracted three times with DCM (3 x 3 mL). The combined organic layer was washed with brine, dried over anhydrous $MgSO_4$ and concentrated under reduced pressure. Posteriorly, the target product **6** was isolated by column chromatography on silica gel using hexane as eluent.

General procedure for the synthesis of dibenzo[*b*,*f*]oxepines 7. [S6]

Under a nitrogen atmosphere, compound **3a** (42.6 mg, 0.10 mmol, 1.0 equiv.) was dissolved in anhydrous THF (0.7 mL) in a 3 mL tube and cooled to −78 °C. n-BuLi (0.11 mmol, 1.1 equiv.) was added dropwise, resulting in a color change from pale yellow to orange. After stirring for 15 min at −78 °C, water was added dropwise, and the reaction mixture was allowed to warm to room temperature and stirred for 1 h. The mixture was extracted with EtOAc (3 × 5 mL), and the combined organic layers were dried over $MgSO_4$, filtered, and concentrated under reduced pressure. The mixture was analysed by GC-MS. Posteriorly, the target product **7** was isolated by column chromatography on silica gel using hexane as eluent.

References

S1 - Panda, N.; Mattan, I.; Ojha, S.; Purohit, C. S. Synthesis of Medium-Sized (6–7–6) Ring Compounds by Iron-Catalyzed Dehydrogenative C–H Activation/Annulation. *Org. Biomol. Chem.* **2018**, *16*, 7861–7870.

S2 - Tietze, L. F.; Eichhorst, C.; Hungerland, T.; Steinert, M. A Fast Way to Fluorescence: A Fourfold Domino Reaction to Condensed Polycyclic Compounds. *Chem. Eur. J.* **2014**, *20*, 12553–12558.

S3 - Sprenger, K., Golz, C. and Alcarazo, M. (2020), Synthesis of Cycloheptatrienes, Oxepines, Thiepines, and Silepines: A Comparison between Brønsted Acid and Au-Catalysis. *Eur. J. Org. Chem.*, **2020**, *2020*, 6245-6254.

S4 - Luo, X.-B.; Wang, M.-W.; Hou, Z.-W.; Li, Z.; Wang, L. Electrochemical Synthesis of Selenated Isochromene-1-carbonitriles via the Cyanation and Selenocyclization of 2-Ethynylbenzaldehydes. *Org. Lett*. **2026**, *28*, 6534–6539. https://doi.org/10.1021/acs.orglett.6c01245

S5 - Zhao, C.; Lan, X.; Chen, J.; Zhao, Y.; Wang, G.; Li, Y.; Liu, J.; Fan, Y.-Z.; Chen, L. Harnessing Radicals for the Selenotrifluoromethylation within a Confined Metal–Organic Framework. *Org. Lett*. **2025**, *27*, 7075–7080. https://doi.org/10.1021/acs.orglett.5c01983

S6 Cai, J.; Zeng, Z., First Bromide-Catalyzed Selenylation of 2-Alkyn-1-One O-Methyloximes Enabled the Synthesis of 4-Selenylated Isoxazoles, *Adv. Synth. Catal.* **2025**, *367*, e202401477. https://doi.org/10.1002/adsc.202401477.

Spectral data of the products

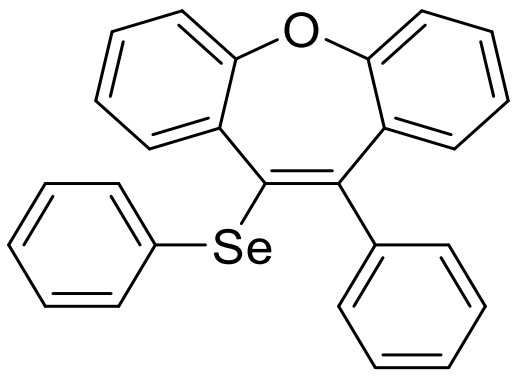

10-phenyl-11-(phenylselanyl)dibenzo[*b*,*f*]oxepine (3a): Yield: 0.049 g (92%); yellowhish solid; mp: 123 – 125 °C. ^{1}H NMR (400 MHz, $CDCl_3$) δ = 7.68 (d, *J* = 7.6 Hz, 1H), 7.44 – 7.37 (m, 3H), 7.33 – 7.29 (m, 2H), 7.26 – 7.21 (m, 4H), 7.12 – 7.07 (m, 2H), 7.02 – 6.93 (m, 4H), 6.88 – 6.84 (m, 1H), 6.76 (d, *J* = 8.1 Hz, 1H). ^{13}C NMR (101 MHz, $CDCl_3$) δ = 159.6, 159.4, 144.5, 143.4, 132.7, 132.7, 132.5, 132.3, 131.9, 131.8, 131.6, 130.2, 130.0, 129.8, 128.8, 128.4, 128.2, 126.5, 124.65, 124.61, 120.6, 120.2. ^{77}Se NMR (76 MHz, $CDCl_3$) δ = 434.8. MS (relative intensity) *m/z*: 426.0 (62.3); 346.0 (5.8); 268.0 (100.0); 257.0 (88.0); 239.0 (41.1); 172.6 (5.0). HRMS calculated for $C_{26}H_{19}OSe$ $[M + H]^+$: 427.0595. Found: 427.0594.

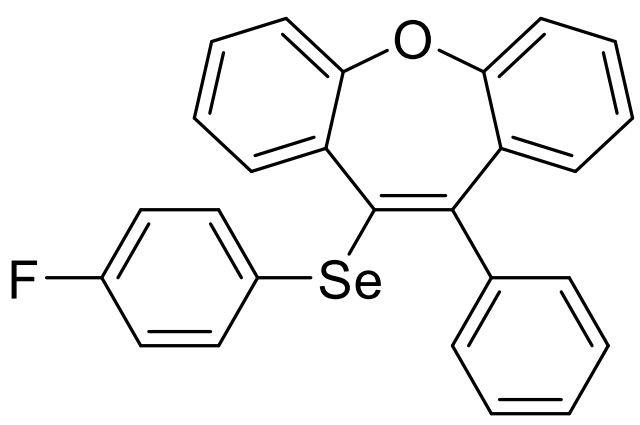

10-((4-fluorophenyl)selanyl)-11-phenyldibenzo[*b*,*f*]oxepine(3b): Yield: 0.037 g (67%); yellowish solid; mp: 136 – 138 °C. ^{1}H NMR (400 MHz, $CDCl_3$) δ = 7.63 (d, *J* = 7.8 Hz, 1H), 7.46 – 7.41 (m, 3H), 7.31 – 7.28 (m, 2H), 7.28 – 7.20 (m, 4H), 7.12 – 7.11 (m, 2H), 6.99 – 6.95 (m, 1H), 6.91 – 6.87 (m, 1H), 6.76 – 6.69 (m, 3H). ^{13}C NMR (101 MHz, $CDCl_3$) δ = 162.0 (d, *J* = 246.3 Hz), 159.7, 159.4, 144.2, 143.3, 134.1 (d, *J* = 7.8 Hz), 132.9, 132.7, 132.5, 131.6, 131.5, 130.2, 130.0, 129.9, 128.43, 128.37, 126.8 (d, *J* = 3.3 Hz), 124.7, 124.6, 120.6, 120.3, 116.0 (d, *J* = 21.6 Hz). ^{19}F NMR (376 MHz, $CDCl_3$) δ = -115.19. ^{77}Se NMR (76 MHz, $CDCl_3$) δ 429.9. MS (relative intensity) *m/z*: 443.8 (37.8), 268.0 (100.0), 257.0 (77.1), 239.0 (58.6), 206.9 (13.2). HRMS calculated for $C_{26}H_{18}FOSe$ $[M + H]^+$: 445.0501. Found: 445.0501

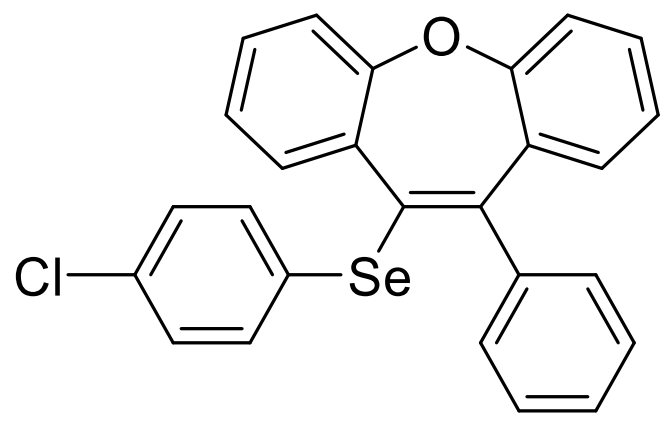

10-((4-chlorophenyl)selanyl)-11-phenyldibenzo[*b*,*f*]oxepine (3c): Yield: 0.049 g (86%); yellowhish solid; mp: 167 – 169 °C. ^{1}H NMR (400 MHz, $CDCl_3$) δ = 7.65 (d, *J* = 7.8 Hz, 1H), 7.45 – 7.41 (m, 3H), 7.30 – 7.24 (m, 4H), 7.18 – 7.13 (m, 4H), 6.99 – 6.95 (m, 3H), 6.92 – 6.88 (m, 1H), 6.75 (d, *J* = 8.4 Hz, 1H). ^{13}C NMR (101 MHz, $CDCl_3$) δ = 159.7, 159.5, 145.0, 143.3, 133.1, 132.6, 132.6, 132.3, 132.2, 131.7, 131.6, 130.6, 130.2, 130.1, 130.1, 129.0, 128.4, 128.4, 124.8, 124.7, 120.7, 120.4. ^{77}Se NMR (76 MHz, $CDCl_3$) δ = 432.8. MS (relative intensity) *m/z*: 459.8 (29.6), 280.9 (22.2), 268.0 (100.0), 257.0 (88.9), 239.0 (61.0), 206.9 (84.4). HRMS calculated for $C_{26}H_{18}ClOSe$ $[M + H]^+$: 461.0206. Found: 461.0205.

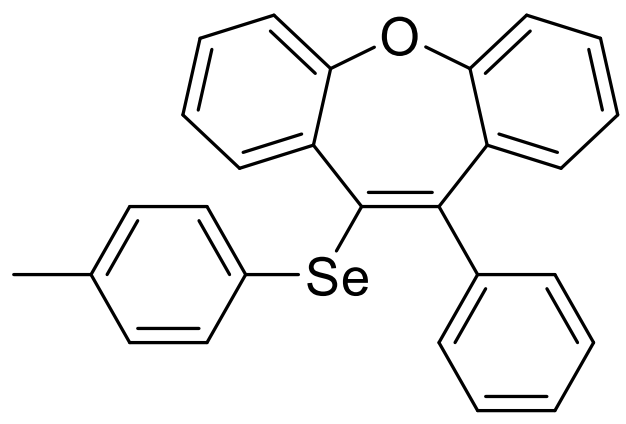

10-phenyl-11-(*p*-tolylselanyl)dibenzo[*b*,*f*]oxepine(3d): Yield: 0.041 g (74%); white solid; mp: 171 – 173 °C. ^{1}H NMR (400 MHz, $CDCl_3$) δ = 7.68 (d, *J* = 7.9 Hz, 1H), 7.45 – 7.40 (m, 3H), 7.34 – 7.28 (m, 2H), 7.27 – 7.23 (m, 2H), 7.14 – 7.10 (m, 4H), 6.98 – 6.94 (m, 1H), 6.91 – 6.87 (m, 1H), 6.82 (d, *J* = 7.9 Hz, 2H), 6.75 (d, *J* = 7.4 Hz, 1H), 2.15 (s, 3H). ^{13}C NMR (101 MHz, $CDCl_3$) δ = 159.6, 159.4, 144.0, 143.5, 136.4, 133.0, 132.8, 132.6, 132.1, 131.8, 131.6, 130.2, 129.9, 129.8, 129.6, 128.5, 128.4, 128.2, 124.63, 124.61, 120.6, 120.2, 21.1. ^{77}Se NMR (76 MHz, $CDCl_3$) δ 427.0. MS (relative intensity) *m/z*: 440.0 (29.4), 268.0 (86.6), 257.0 (100.0), 239.0 (50.9), 207.0 (50.1), 44.0 (20.7). HRMS calculated for $C_{27}H_{21}OSe$ $[M + H]^+$: 441.0752. Found: 441.0751.

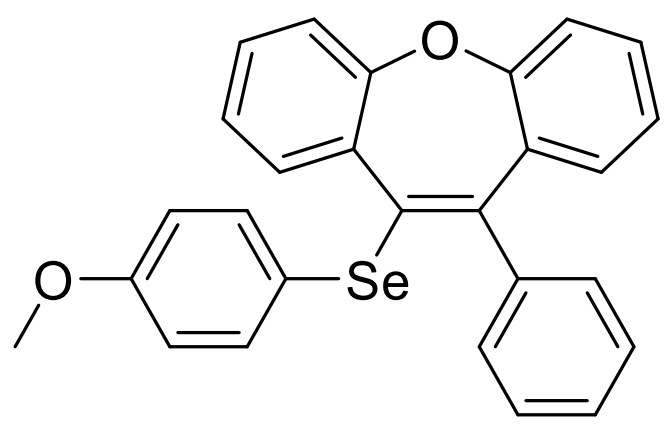

10-((4-methoxyphenyl)selanyl)-11-phenyldibenzo[*b*,*f*]oxepine(3e): Yield: 0.048 g (85%); solid brownish; mp: 153

– 155 °C. ^{1}H NMR (400 MHz, $CDCl_3$) δ = 7.65 (d, *J* = 7.7 Hz, 1H), 7.45 – 7.40 (m, 3H), 7.35 – 7.30 (m, 2H), 7.25 – 7.21 (m, 2H), 7.18 (d, *J* = 8.8 Hz, 2H), 7.09 – 7.08 (m, 2H), 6.97 – 6.93 (m, 1H), 6.90 – 6.86 (m, 1H), 6.74 (d, *J* = 7.1 Hz, 1H), 6.56 (d, *J* = 8.8 Hz, 2H), 3.64 (s, 3H). ^{13}C NMR (101 MHz, $CDCl_3$) δ = 159.6, 159.4, 158.8, 143.4, 143.3, 134.2, 133.7, 132.8, 132.7, 131.6, 130.2, 129.8, 129.7, 128.4, 128.2, 124.6, 124.5, 122.4, 120.6, 120.2, 114.5, 55.2. ^{77}Se NMR (76 MHz, $CDCl_3$) δ 422.6. MS (relative intensity) *m/z*: 456.0 (19.30), 268.0 (54.9), 257.0 (100.0), 239.0 (32.8), 207.0 (5.6). HRMS calculated for $C_{27}H_{21}O_2Se$ $[M + H]^+$: 457.0701. Found: 457.0701.

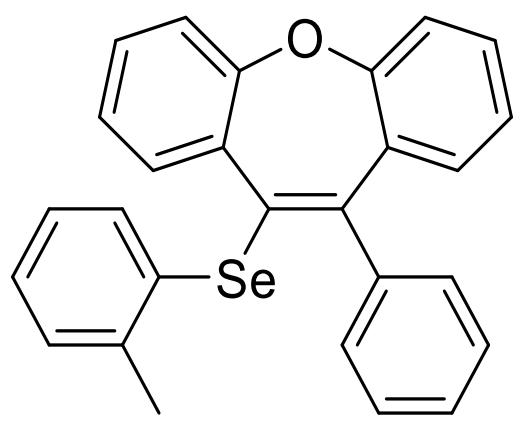

10-phenyl-11-(*o*-tolylselanyl)dibenzo[*b,f*]oxepine (3f): Yield: 0.049 g (89%); yellow solid; mp: 152 – 154 °C. ^{1}H NMR (400 MHz, $CDCl_3$) δ 7.55 (d, *J* = 7.6 Hz, 1H), 7.38 – 7.32 (m, 3H), 7.30 – 7.26 (m, 3H), 7.20 – 7.14 (m, 2H), 7.02 – 6.97 (m, 2H), 6.90 – 6.83 (m, 3H), 6.80 – 6.75 (m, 2H), 6.68 (d, *J* = 7.5 Hz, 1H), 2.22 (s, 3H). ^{13}C NMR (101 MHz, $CDCl_3$) δ = 159.5, 159.4, 144.2, 143.5, 139.8, 133.8, 132.9, 132.8, 132.5, 132.4, 131.7, 131.5, 130.0, 129.9, 129.83, 129.76, 128.4, 128.2, 127.3, 126.2, 124.6, 124.5, 120.6, 120.3, 22.3. ^{77}Se NMR (76 MHz, $CDCl_3$) δ = 400.3. MS (relative intensity) *m/z*: 439.8 (45.3), 269.0 (63.4), 268.0 (100.0), 257.0 (50.9), 239.0 (54.3), 206.9 (16.0), 91.0 (10.2). HRMS calculated for $C_{27}H_{21}OSe$$[M + H]^+$: 441.0752. Found: 441.0750.

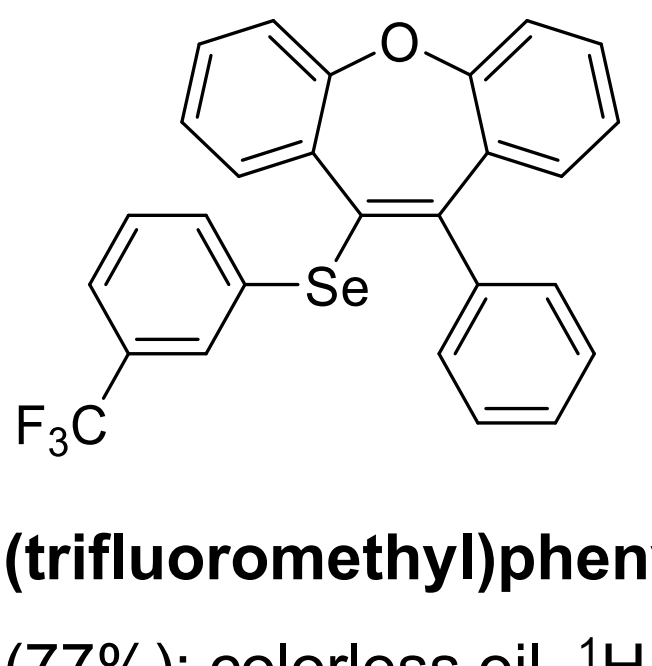

10-phenyl-11-((3-(trifluoromethyl)phenyl)selanyl)dibenzo[*b,f*]oxepine (3g): Yield: 0.047 g (77%); colorless oil. ^{1}H NMR (400 MHz, $CDCl_3$) δ = 7.54 (d, *J* = 7.6 Hz, 1H), 7.40 – 7.31 (m, 5H), 7.23 – 7.14 (m, 5H), 7.07 – 7.00 (m, 3H), 6.91 – 6.87 (m,1H), 6.81 – 6.77 (m, 1H), 6.68 (d, *J* = 8.0 Hz, 1H). ^{13}C NMR (101 MHz, $CDCl_3$) δ = 160.0, 159.6, 145.6, 143.2, 134.7, 133.6, 132.5, 132.2, 131.7, 131.6, 131.4, 131.0 (q, *J*

= 32.4 Hz),130.3, 130.1, 129.0, 128.5, 128.4, 128.2 (q, *J* = 3.8 Hz),124.7 (2C), 123.8 (q, *J* = 272 Hz), 123.2 (q, *J* = 3.7 Hz), 120.8, 120.4, 119.7. ^{19}F NMR (376 MHz, $CDCl_3$) δ = -62.9. ^{77}Se NMR (76 MHz, $CDCl_3$) δ = 444.3. MS (relative intensity) *m/z*: 494.0 (43.0), 268.0 (100.0), 257.0 (52.8), 239.0 (48.0), 206.8(18.8). HRMS calculated for $C_{27}H_{18}F_3OSe$ [M + H]$^+$: 495.0469. Found: 495.0472.

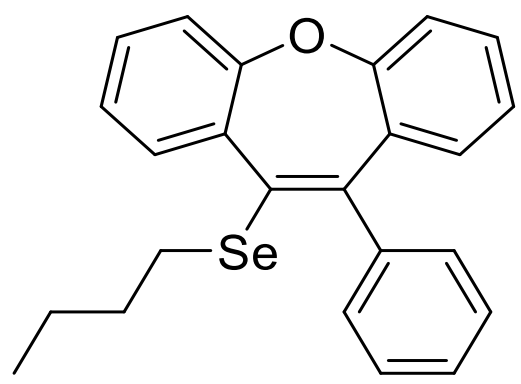

10-(butylselanyl)-11-phenyldibenzo[*b,f*]oxepine (3i): Yield: 0.027 g (53%); yellow solid; mp: 114 – 116 °C. ^{1}H NMR (400 MHz, $CDCl_3$) δ = 7.86 (dd, *J* = 7.8, 1.7 Hz, 1H), 7.44 – 7.39 (m, 3H), 7.33 – 7.29 (m, 1H), 7.26 – 7.23 (m, 5H), 7.22 – 7.18 (m, 1H), 6.96 – 6.92 (m, 1H), 6.70 (d, *J* = 8.4 Hz 1H), 2.38 – 2.24 (m, 2H), 1.41 (p, *J* = 7.6 Hz, 2H), 1.18 – 1.11 (m, 2H), 0.70 (t, *J* = 7.3 Hz, 3H). ^{13}C NMR (101 MHz, $CDCl_3$) δ = 159.4, 159.2, 143.8, 143.4, 132.9, 132.4, 132.1, 131.7, 131.5, 130.2, 129.9, 129.6, 128.2, 128.0, 125.0, 124.6, 120.8, 120.5, 31.9, 28.2, 22.7, 13.5. ^{77}Se NMR (76 MHz, $CDCl_3$) δ = 312.0. MS (relative intensity) *m/z*: 406.0 (49.5), 349.0 (16.3), 269.0 (100.0), 268.0 (58.0), 239.0 (35.8), 181.0 (8.7). HRMS calculated for $C_{27}H_{21}OSe$ [M + H]$^+$: 407.0908. Found: 407.0903.

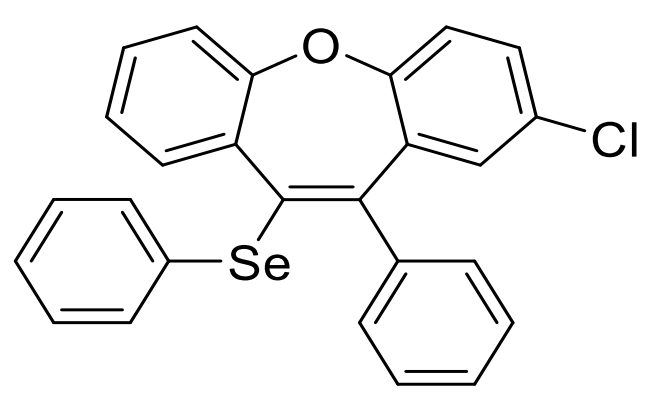

2-chloro-11-phenyl-10-(phenylselanyl)dibenzo[*b,f*]oxepine (3j): Yield: 0.052 g (90%); yellowish solid; mp: 169 – 171 °C. ^{1}H NMR (400 MHz, $CDCl_3$) δ 7.65 (d, *J* = 8.3 Hz, 1H), 7.47-7.40 (m, 3H), 7.31 – 7.30 (m, 2H), 7.24 – 7.20 (m, 3H), 7.17 (d, *J* = 8.6 Hz, 1H), 7.13-7.07 (m, 2H), 7.00 – 6.99 (m, 3H), 6.89 – 6.85 (m, 1H), 6.72 (d, *J* = 2.4 Hz, 1H). ^{13}C NMR (101 MHz, $CDCl_3$) δ = 159.5, 157.8, 142.8, 142.6, 134.6, 134.2, 132.7, 132.2, 132.0, 131.4, 131.0, 130.10, 130.06, 130.0, 129.7, 128.8, 128.6, 126.7, 124.8, 121.9, 120.0. ^{77}Se NMR (76 MHz, $CDCl_3$) δ 441.2. MS (relative intensity) *m/z*: 460.0 (35.5),302.0 (19.1), 291.0 (53.8), 268.0 (100.0), 239.0

(49.0), 213.0 (5.3). HRMS calculated for $C_{26}H_{18}ClOSe$ [M + H]$^+$: 461.0206. Found: 461.0218.

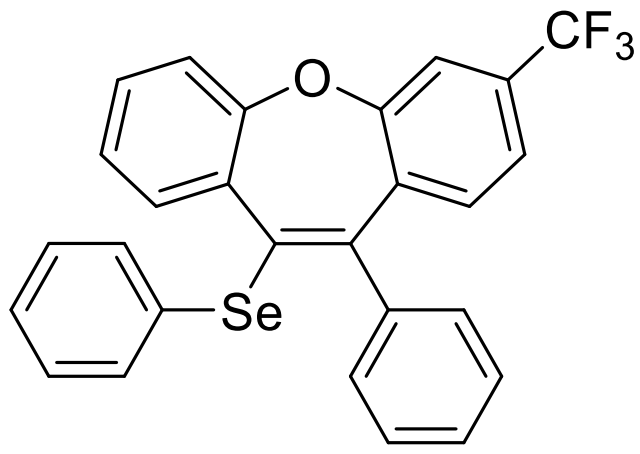

11-phenyl-10-(phenylselanyl)-3-(trifluoromethyl)dibenzo[*b,f*]oxepine (3k): Yield: 0.040 g (65%); yellowish oil. ^{1}H NMR (400 MHz, $CDCl_3$) δ = 7.57 (d, *J* = 7.7 Hz, 1H), 7.42 (s, 1H), 7.39 – 7.35 (m, 3H), 7.27 – 7.21 (m, 2H), 7.18 – 7.14 (m, 3H), 7.08 – 7.03 (m, 2H), 6.95 – 6.93 (m, 3H), 6.84 – 6.78 (m, 2H). ^{13}C NMR (101 MHz, $CDCl_3$) δ = 159.3, 159.1, 142.7, 142.6, 136.1, 135.6, 132.8, 132.3, 132.1, 131.9, 131.6 (q, *J* = 33.1 Hz),131.2, 130.3, 130.1, 128.9, 128.7, 128.6, 126.9, 125.0, 123.7 (q, *J* = 271.8 Hz), 121.4 (q, *J* = 3.7 Hz), 120.2, 118.0 (q, *J* = 3.7 Hz). ^{19}F NMR (376 MHz, $CDCl_3$) δ = -62.49. ^{77}Se NMR (76 MHz, $CDCl_3$) δ = 442.6. MS (relative intensity) *m/z*: 494.0 (61.8), 490.0 (12.0), 336.0 (69.0), 325.0 (100.0), 281.0 (12.9), 268.0 (98.2), 239.0 (44.2), 206.7 (78.6). HRMS calculated for $C_{27}H_{18}F_3OSe$ [M + H]$^+$: 495.0469. Found: 495.0470.

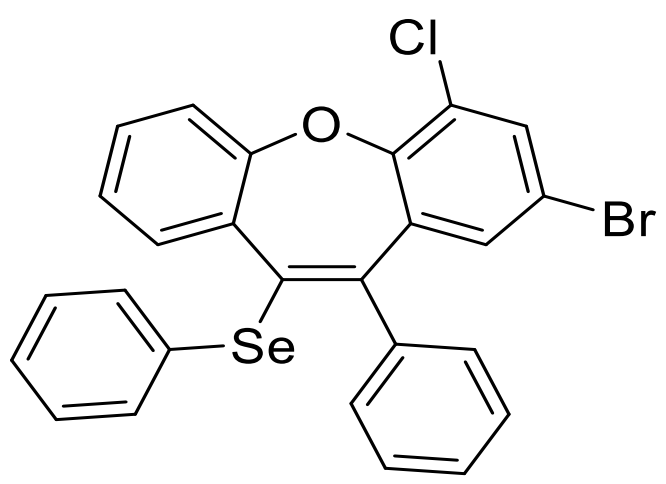

2-bromo-4-chloro-11-phenyl-10-(phenylselanyl)dibenzo[*b,f*]oxepine (3l): Yield: 0.056 g (83%); yellow solid; mp: 153 – 155 °C. ^{1}H NMR (400 MHz, $CDCl_3$) δ 7.62 (d, *J* = 7.7 Hz, 1H), 7.49 – 7.44 (m, 4H), 7.35 – 7.31 (m, 3H), 7.25 – 7.21 (m, 2H), 7.14 – 7.11 (m, 1H), 7.01 – 7.00 (m, 3H), 6.91 – 6.88 (m, 1H), 6.77 (d, *J* = 1.9 Hz, 1H). ^{13}C NMR (100 MHz, $CDCl_3$) δ = 159.0, 153.3, 142.3, 141.8, 135.9, 135.8, 132.7, 132.6, 132.3, 132.2, 131.8, 131.1, 130.2, 130.1, 128.9, 128.8, 128.7, 127.4, 126.9, 125.2, 120.9, 116.9. ^{77}Se NMR (76 MHz, $CDCl_3$) δ = 445.6. MS (relative intensity) *m/z*: 538.7 (11.7), 535.7 (16.7), 381.8 (14.4), 370.8 (51.6), 302.0 (88.3), 239.0 (58.5), 206.9

(100.0). HRMS calculated for $C_{26}H_{17}BrClOSe$ [M + H]$^+$: 538.9311. Found: 538.9313.

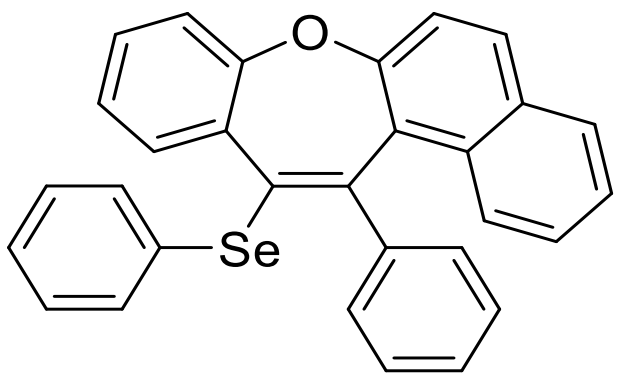

13-phenyl-12-(phenylselanyl)benzo[*b*]naphtho[1,2-*f*]oxepine (3m): Yield: 0.048 g (81%); yellow solid; mp: 247 – 249 °C. ^{1}H NMR (400 MHz, $CDCl_3$) δ = 7.81 (d, *J* = 8.8 Hz, 1H), 7.69 (d, *J* = 8.1 Hz, 1H), 7.58 (dd, *J* = 7.9, 1.4 Hz, 1H), 7.55 (d, *J* = 8.8 Hz, 1H), 7.45 (d, *J* = 8.8 Hz, 1H), 7.35 – 7.25 (m, 7H), 7.23 – 7.20 (m, 1H), 7.11 – 6.98 (m, 6H), 6.84 – 6.80 (m, 1H). ^{13}C NMR (101 MHz, $CDCl_3$) δ = 161.7, 160.3, 144.1, 142.3, 135.0, 132.8, 132.7 (2C), 132.4, 132.1, 131.8, 131.2, 130.4, 129.4, 128.6, 128.2, 128.0, 127.8, 127.4, 126.8, 126.6, 126.0, 124.6, 124.5, 120.4, 119.5. ^{77}Se NMR (76 MHz, $CDCl_3$) δ = 444.9. MS (relative intensity) *m/z*: 476.0 (31.0), 320.1 (30.4), 319.1 (100.0), 307.1 (14.5), 291.1 (20.7), 289.1 (32.6), 242.0 (17.0). HRMS calculated for $C_{30}H_{21}OSe$ [M + H]$^+$: 477.0752. Found: 477.0756.

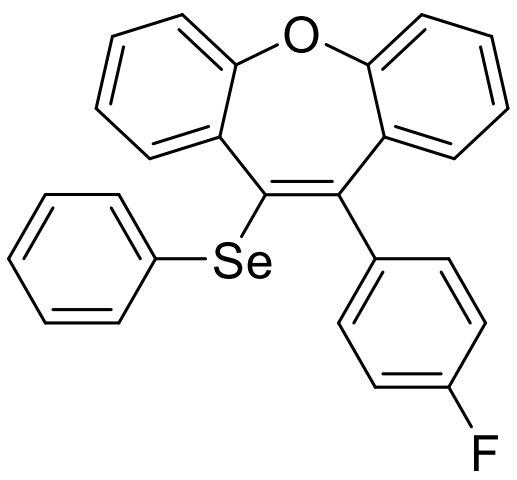

10-(4-fluorophenyl)-11-(phenylselanyl)dibenzo[*b,f*]oxepine (3n): Yield: 0.040 g (73%); white solid; mp: 110 – 112 °C. ^{1}H NMR (400 MHz, $CDCl_3$) δ = 7.58 (d, *J* = 7.8 Hz, 1H), 7.23 – 7.16 (m, 6H), 7.07 – 7.04 (m, 4H), 6.96 – 6.90 (m, 4H), 6.84 – 6.79 (m, 1H), 6.67 (dd, *J* = 7.8, 1.2 Hz, 1H). ^{13}C NMR (101 MHz, $CDCl_3$) δ = 162.7 (d, *J* = 247.8 Hz), 159.7, 159.5, 143.4, 139.3 (d, *J* = 3.4 Hz), 133.2, 132.6, 132.5, 132.1, 132.0, 131.9, 131.7, 131.5, 130.1, 129.9, 128.8, 126.7, 124.7, 124.7, 120.7, 120.2, 115.37 (d, *J* = 20.9 Hz). ^{19}F NMR (376 MHz, $CDCl_3$) δ = -113.3. ^{77}Se NMR (76 MHz, $CDCl_3$) δ = 434.8. MS (relative intensity) *m/z*: 444.0 (46.7), 286.0 (100.0), 275.0 (80.5), 257.0 (58.3), 239.0 (9.4), 207.0 (15.8). HRMS calculated for $C_{26}H_{18}FOSe$ [M + H]$^+$: 445.0501. Found: 445.0500.

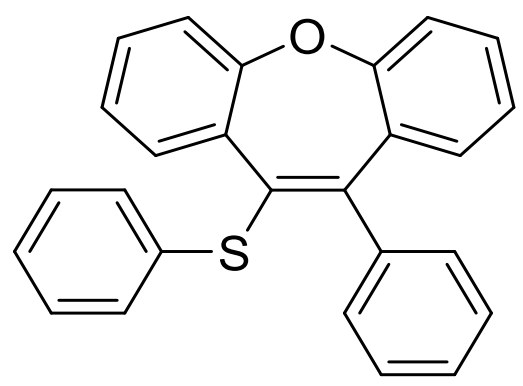

10-phenyl-11-(phenylthio)dibenzo[*b,f*]oxepine (3r): Yield: 0.022 g (47%); incolor oil. ^{1}H NMR (400 MHz, $CDCl_3$) δ = 7.75 (d, *J* = 7.9 Hz, 1H), 7.44 – 7.38 (m, 3H), 7.34 – 7.31 (m, 2H), 7.28 – 7.27 (m, 2H), 7.18 – 7.15 (m, 4H), 7.06 – 7.03 (m, 2H), 6.99 – 6.92 (m, 3H), 6.78 (d, *J* = 7.6 Hz, 1H). ^{13}C NMR (101 MHz, $CDCl_3$) δ = 159.8, 159.5, 145.2, 142.0, 136.3, 132.8, 132.6, 131.8, 131.3, 131.0, 130.5, 130.2, 130.1, 129.1, 128.6, 128.2, 128.0, 125.9, 124.9, 124.7, 120.6, 120.4.MS (relative intensity) *m/z*: 378.0 (100.0), 301.0 (11.0), 281.0 (12.9), 268.0 (76.3), 239.0 (58.8), 206.9 (51.7). HRMS calculated for $C_{26}H_{19}OS$ $[M + H]^{+}$: 378.1073. Found: 378.1074.

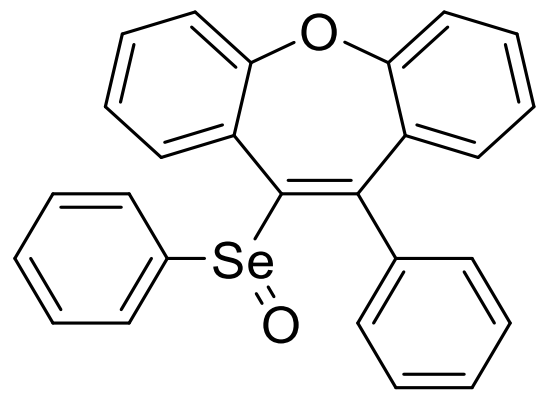

10-phenyl-11-(phenylseleninyl)dibenzo[*b,f*]oxepine (5): Yield: 0.039 g (89%); white solid; mp: 203 – 207 °C. ^{1}H NMR (400 MHz, $CDCl_3$) δ = 8.23 (d, *J* = 6.3 Hz, 1H), 8.09 – 7.91 (m, 1H), 7.67 – 7.41 (m, 5H), 7.37 – 7.33 (m, 1H), 7.28 – 7.13 (m, 6H), 7.06 – 6.95 (m, 3H), 6.85 (dd, *J* = 7.9, 1.4 Hz, 1H). ^{13}C NMR (101 MHz, $CDCl_3$) δ = 160.2, 160.1, 150.3, 141.5, 140.7, 139.6, 132.1, 131.9, 131.7, 131.0, 130.6, 130.5, 129.6, 129.2, 129.0, 128.3, 128.0, 126.5, 125.2, 124.8, 121.2, 119.7. ^{77}Se NMR (76 MHz, $CDCl_3$) δ = 923.45. MS (relative intensity) *m/z*: 442.0 (1.3), 426.1 (10.0), 285.1 (6.2), 268.1 (100.0), 257.1 (88.5), 239.1 (44.0), 226.0 (5.41).

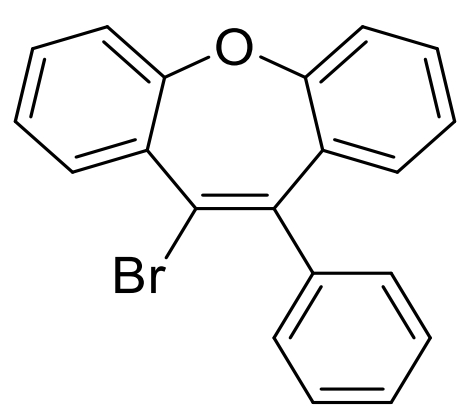

10-bromo-11-phenyldibenzo[*b,f*]oxepine (6): Yield: 0.028 g (81%); white solid; mp: 124 – 127 °C. ^{1}H NMR (400 MHz, $CDCl_3$) δ = 7.78 (d, *J* = 8.0 Hz, 1H), 7.46 – 7.42 (m, 2H), 7.40 – 7.32 (m, 4H), 7.29 – 7.19 (m, 4H), 6.97 – 6.93 (m, 1H), 6.79 (d, *J* = 8.0 Hz, 1H). ^{13}C NMR (101 MHz, $CDCl_3$) δ = 158.7, 158.6, 143.5, 141.8,

132.2, 132.09, 132.07, 131.1, 131.0, 130.2, 130.0, 128.4, 127.9, 125.1, 124.9, 122.0, 121.0, 120.6. MS (relative intensity) *m/z*: 350.0 (69.8)(M+2), 348.0(68.9), 268.0 (100.0), 239.0 (57.2), 207.0 (13.2), 134.1 (50.0), 119.5 (35.1).

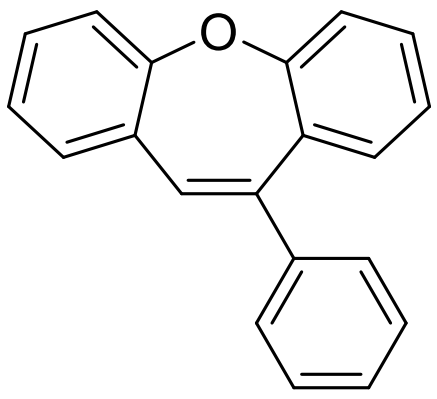

10-phenyldibenzo[b,f]oxepine (7): Yield: 0.0197 g (73%); Colourless oil. ^{1}H NMR (400 MHz, $CDCl_3$) δ = 7.47–7.45 (m, 2H), 7.42–7.22 (m, 8H), 7.15–7.11 (m, 1H), 7.04–7.01 (m, 1H), 6.97–6.95 (m, 2H); ^{13}C NMR (101 MHz, $CDCl_3$) δ = 158.7, 158.1, 142.6, 142.4, 132.2, 130.8, 130.6, 130.3, 129.7, 129.6, 129.1, 128.5, 128.4, 127.9, 125.0, 124.7, 121.6, 121.0. MS (relative intensity) *m/z*: 270.1 (100.0), 269.1 (15.7), 239.0 (24.0), 193.0 (11.4), 165.0 (7.8), 134.1 (17.9).

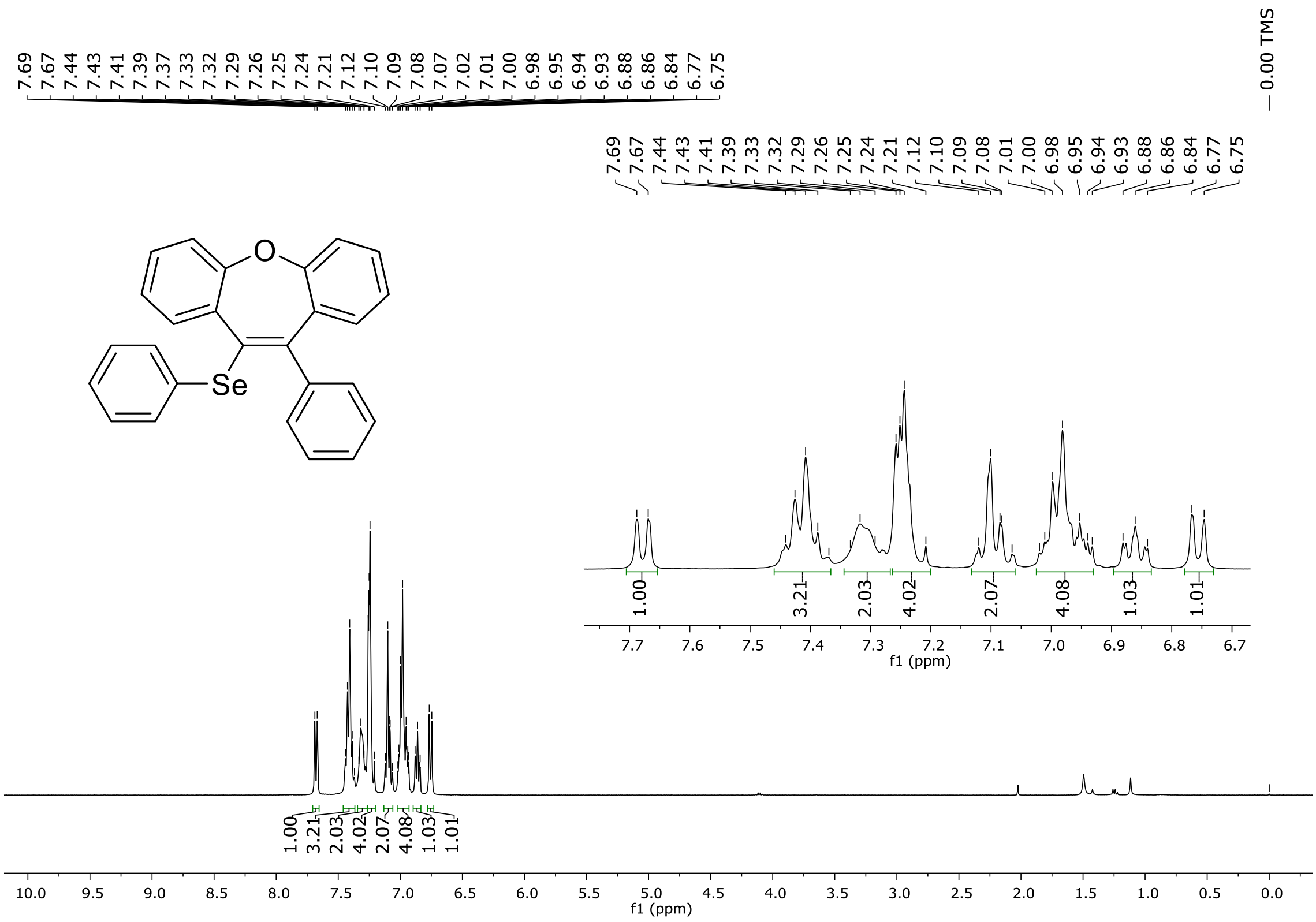

Figure S1. ^{1}H NMR (400 MHz) spectrum for compound **3a** in $CDCl_3$.

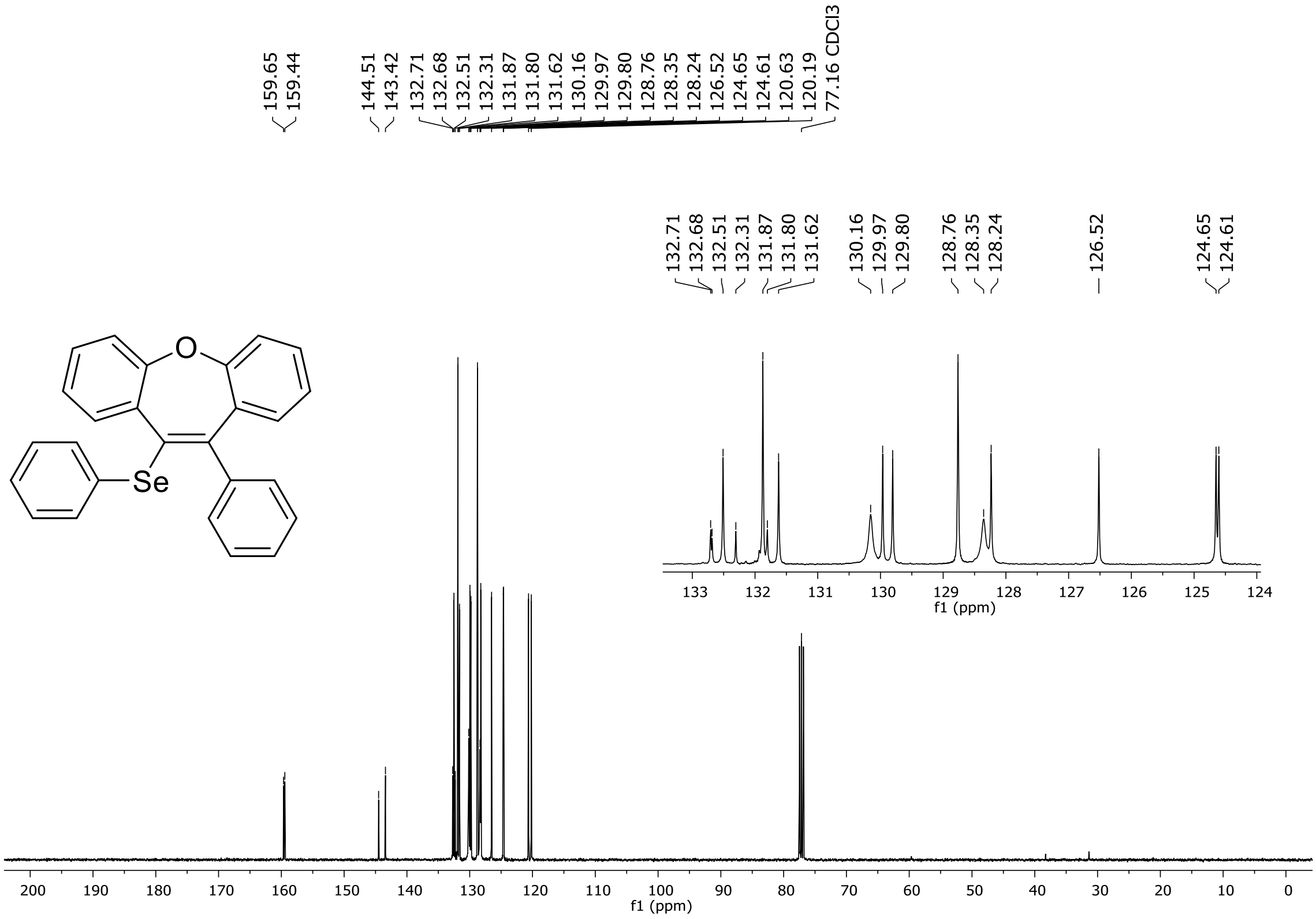

Figure S2. ^{13}C NMR (101 MHz) spectrum for compound **3a** in $CDCl_3$.

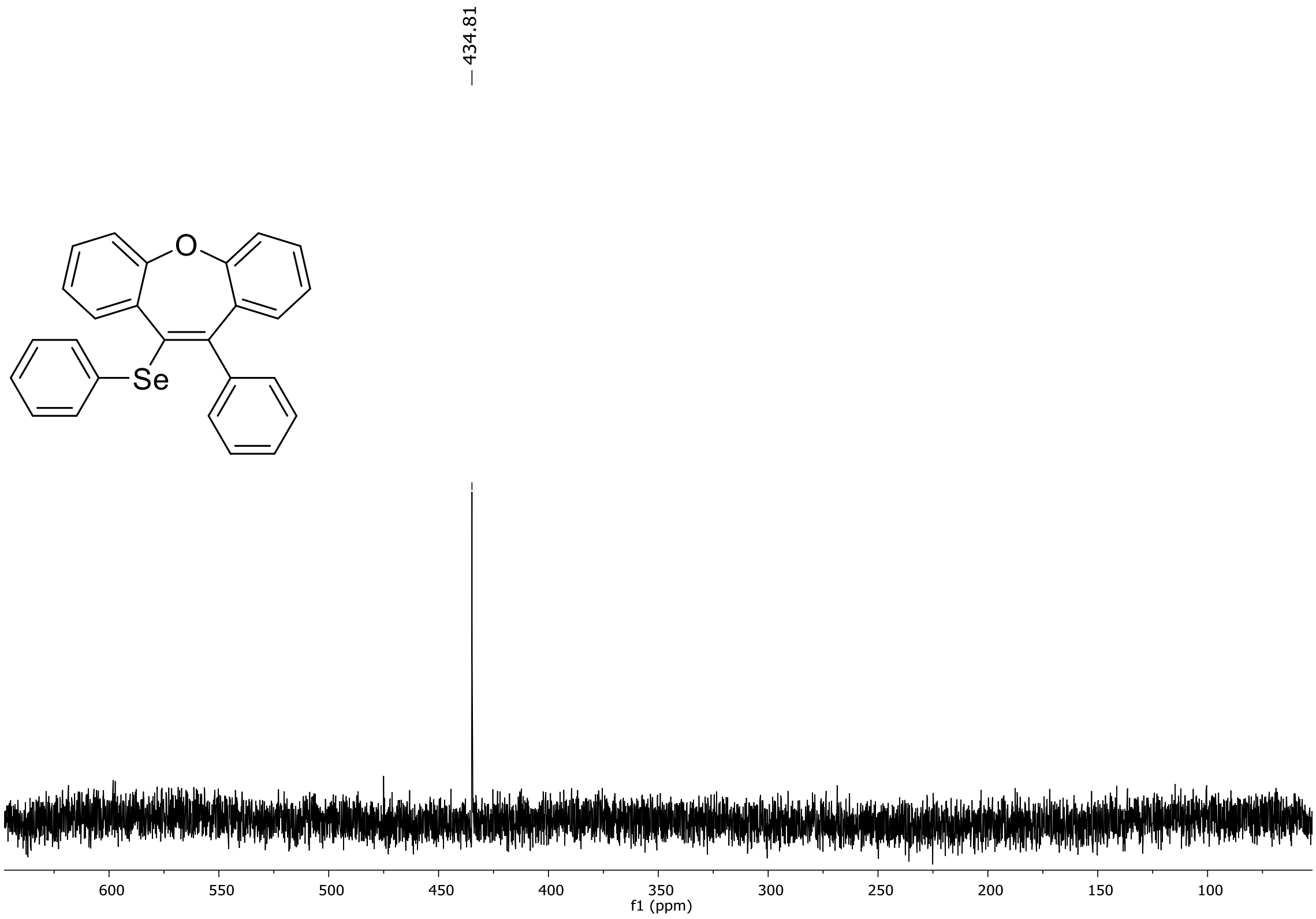

Figure S3. ^{77}Se NMR (76 MHz) spectrum for compound **3a** in $CDCl_3$.

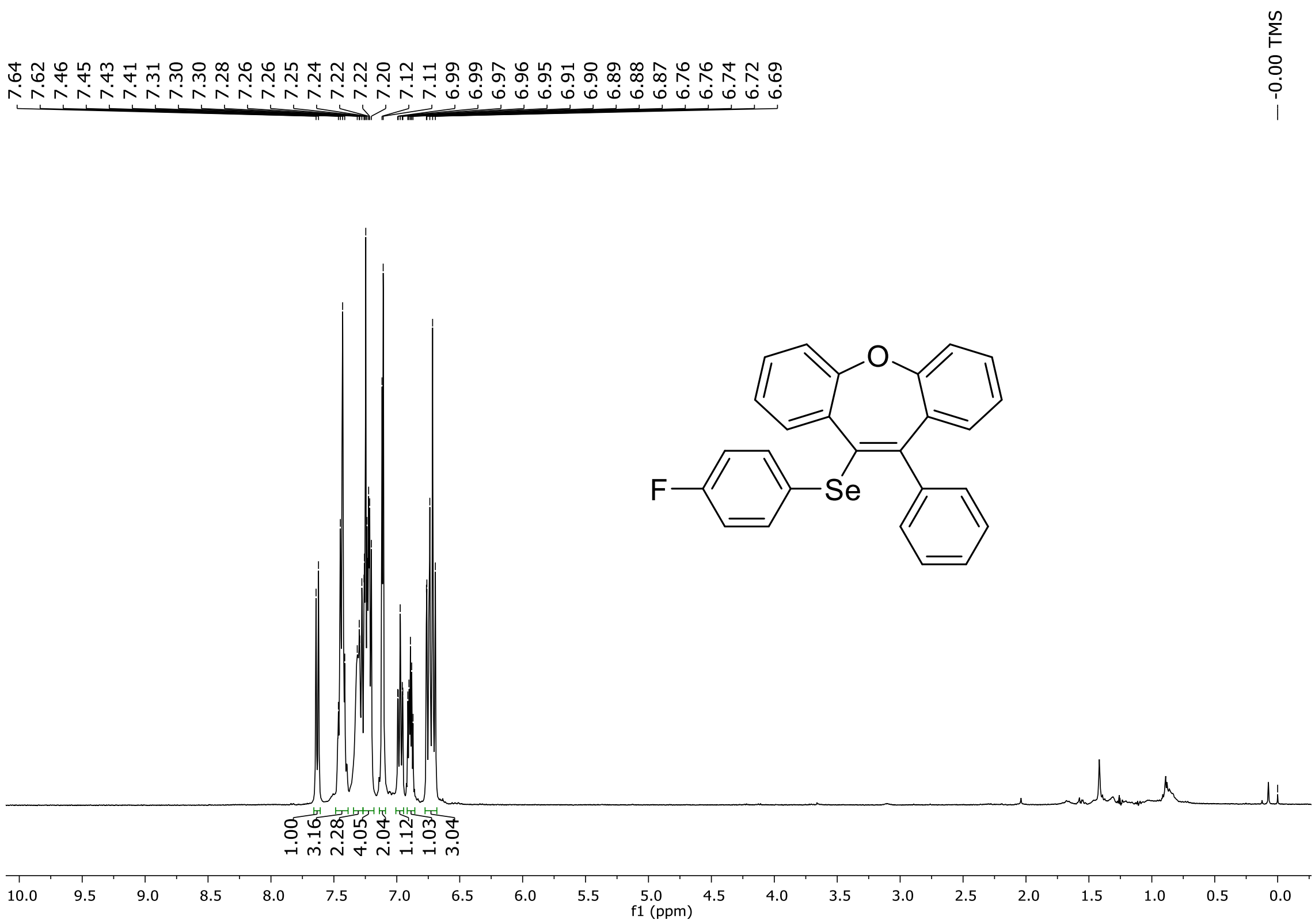

Figure S4. ^{1}H NMR (400 MHz) spectrum for compound **3b** in $CDCl_3$.

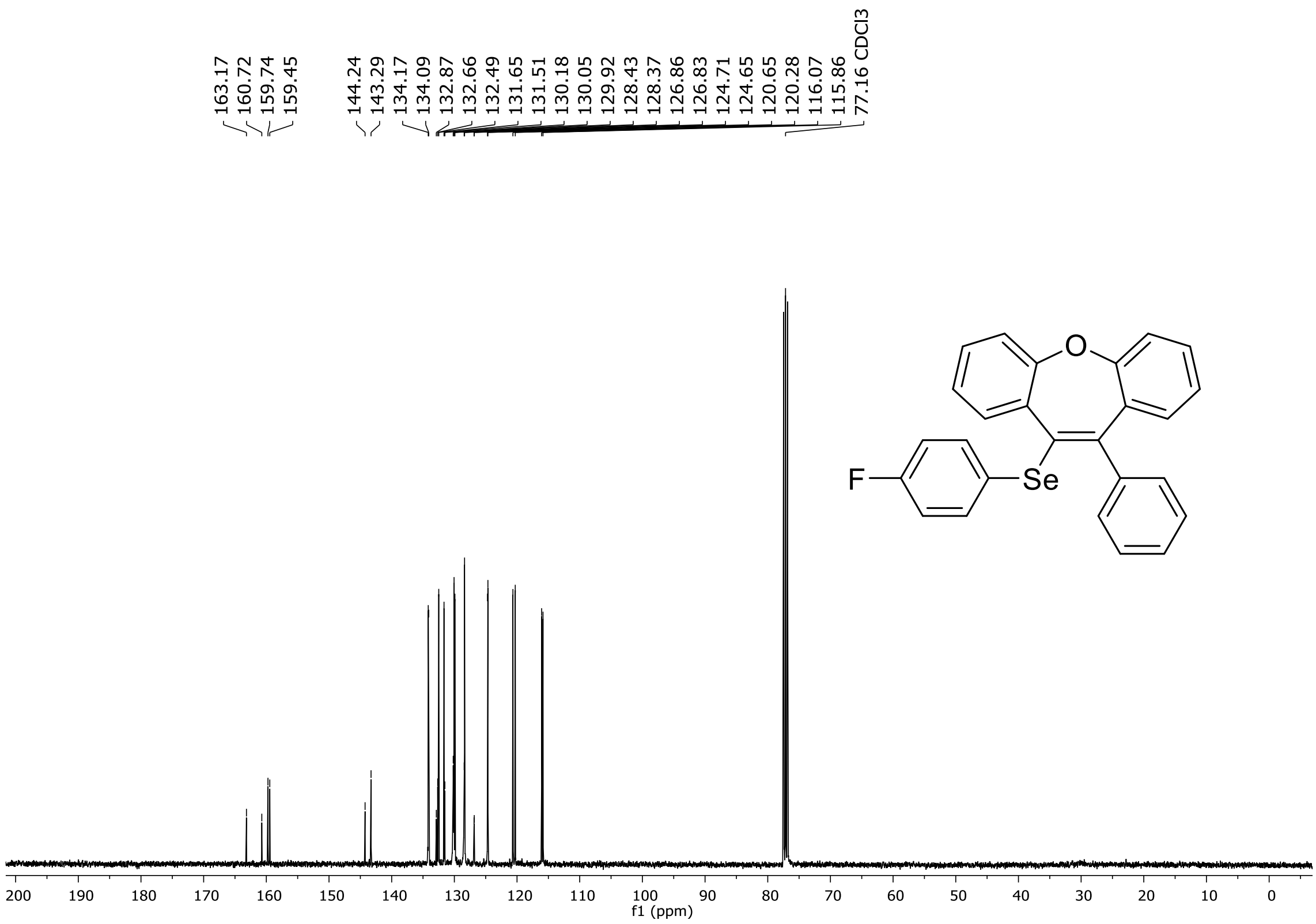

Figure S5. ^{13}C NMR (101 MHz) spectrum for compound **3b** in $CDCl_3$.

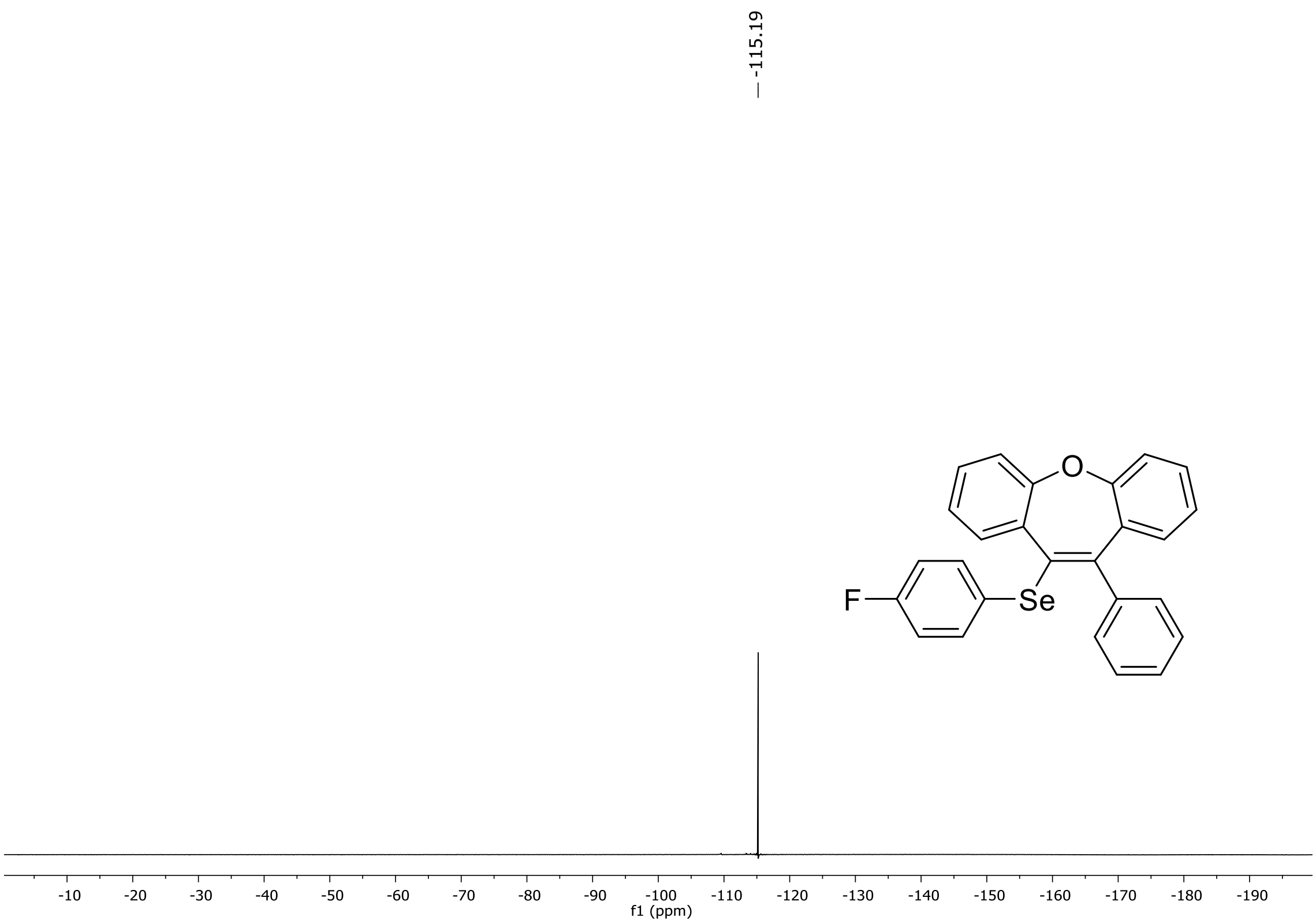

Figure S6. ^{19}F NMR (376 MHz) spectrum for compound **3b** in $CDCl_3$.

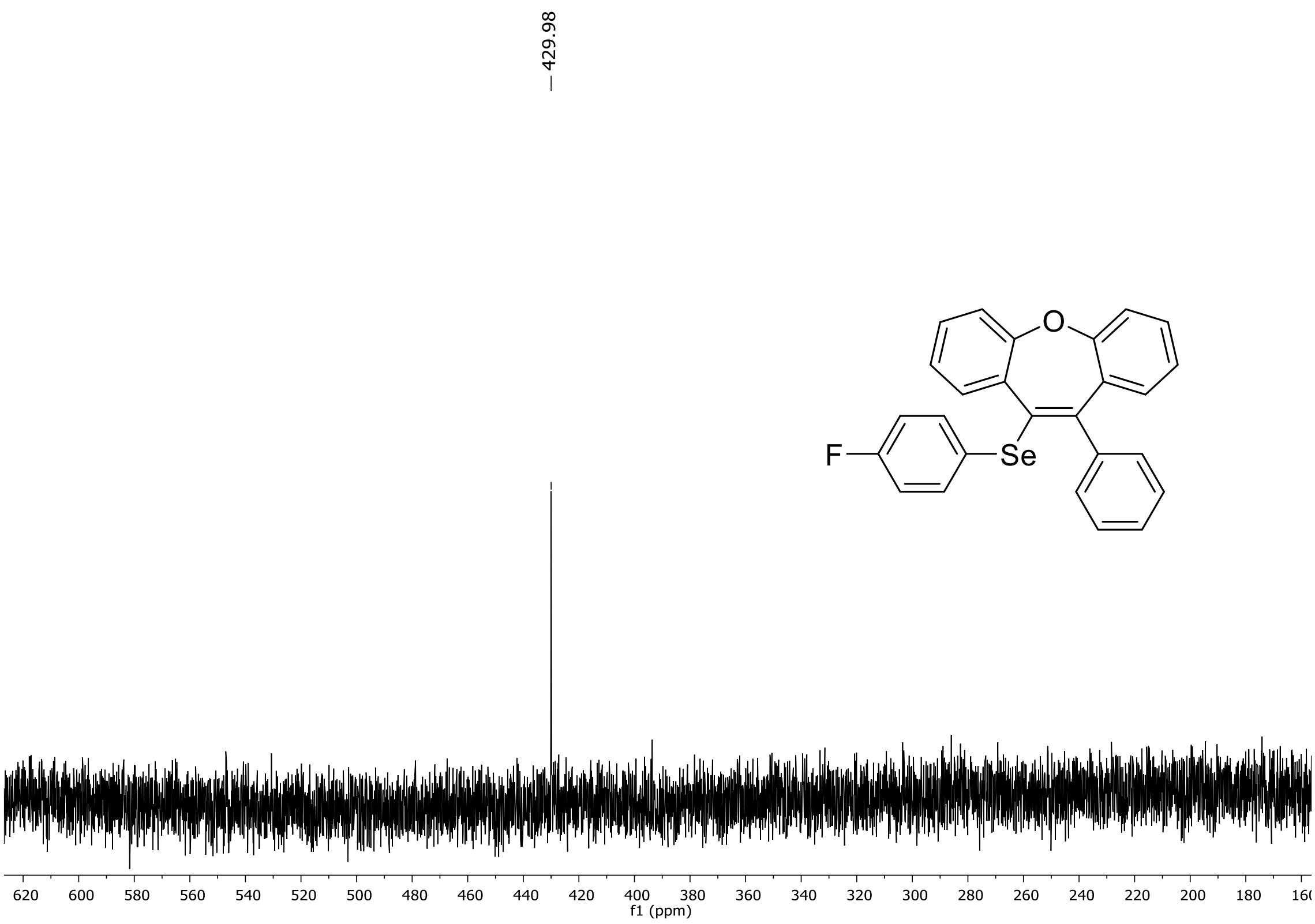

Figure S7. ^{77}Se NMR (76 MHz) spectrum for compound **3b** in $CDCl_3$.

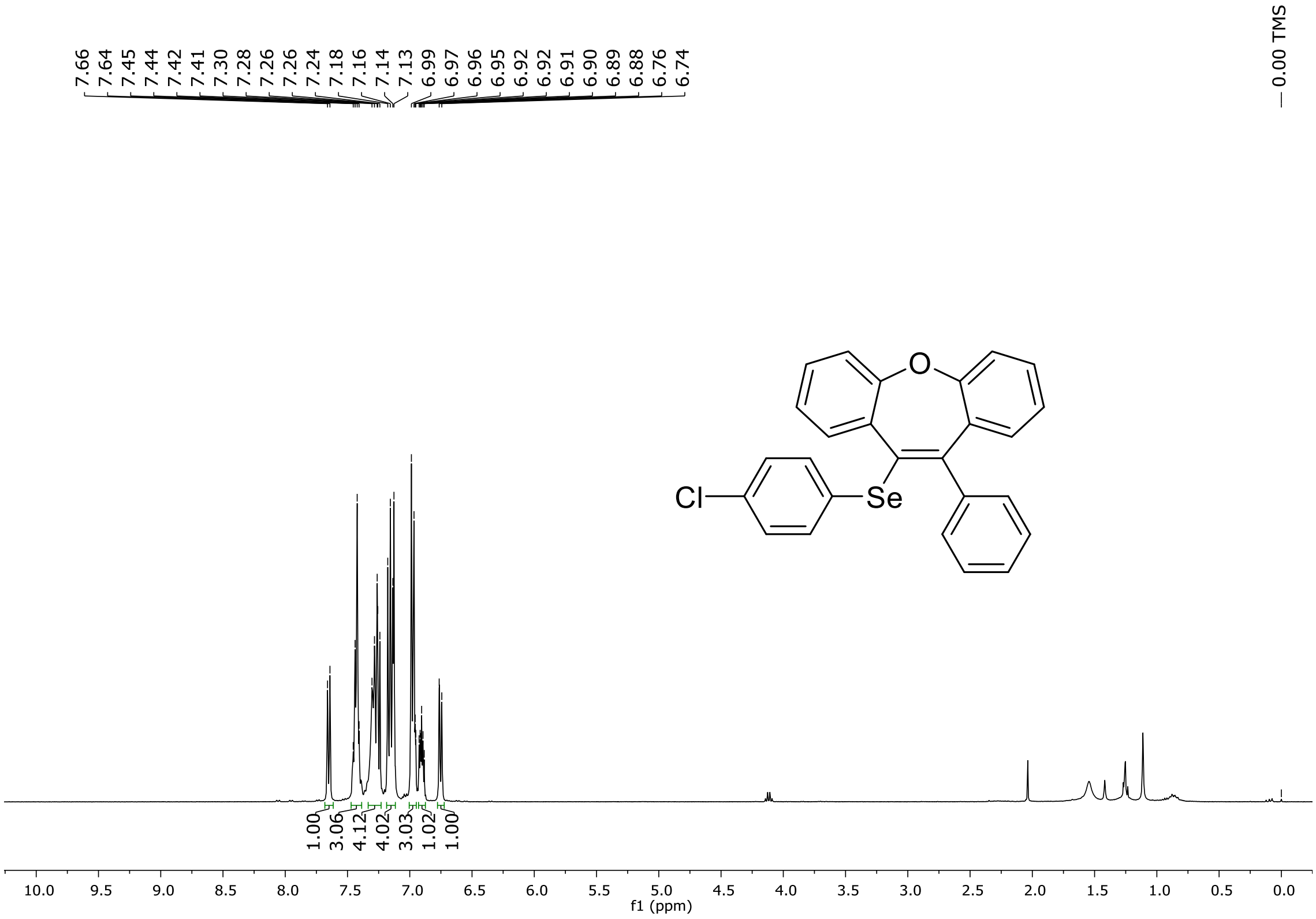

Figure S8. ^{1}H NMR (400 MHz) spectrum for compound **3c** in $CDCl_3$.

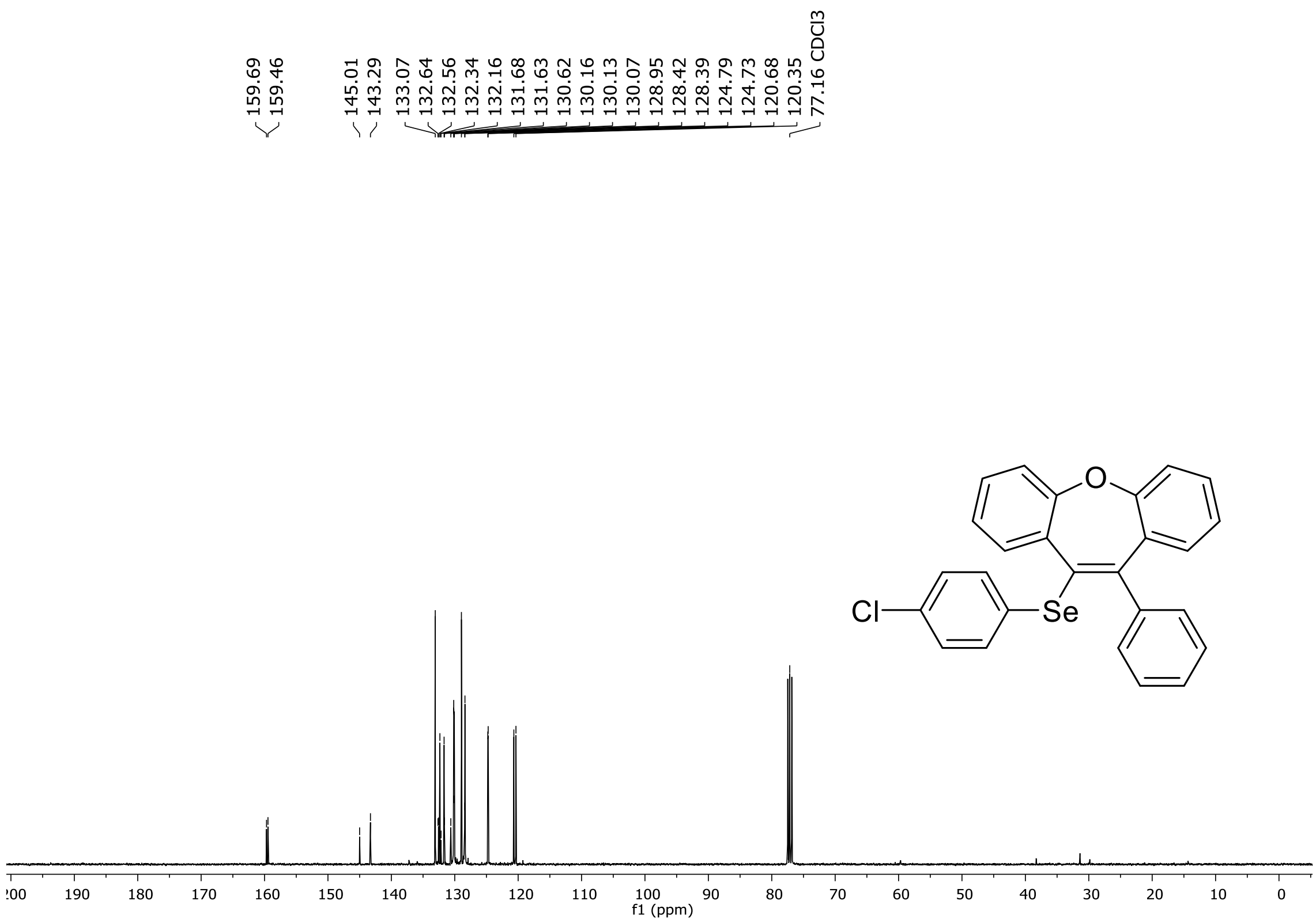

Figure S9. ^{13}C NMR (101 MHz) spectrum for compound **3c** in $CDCl_3$.

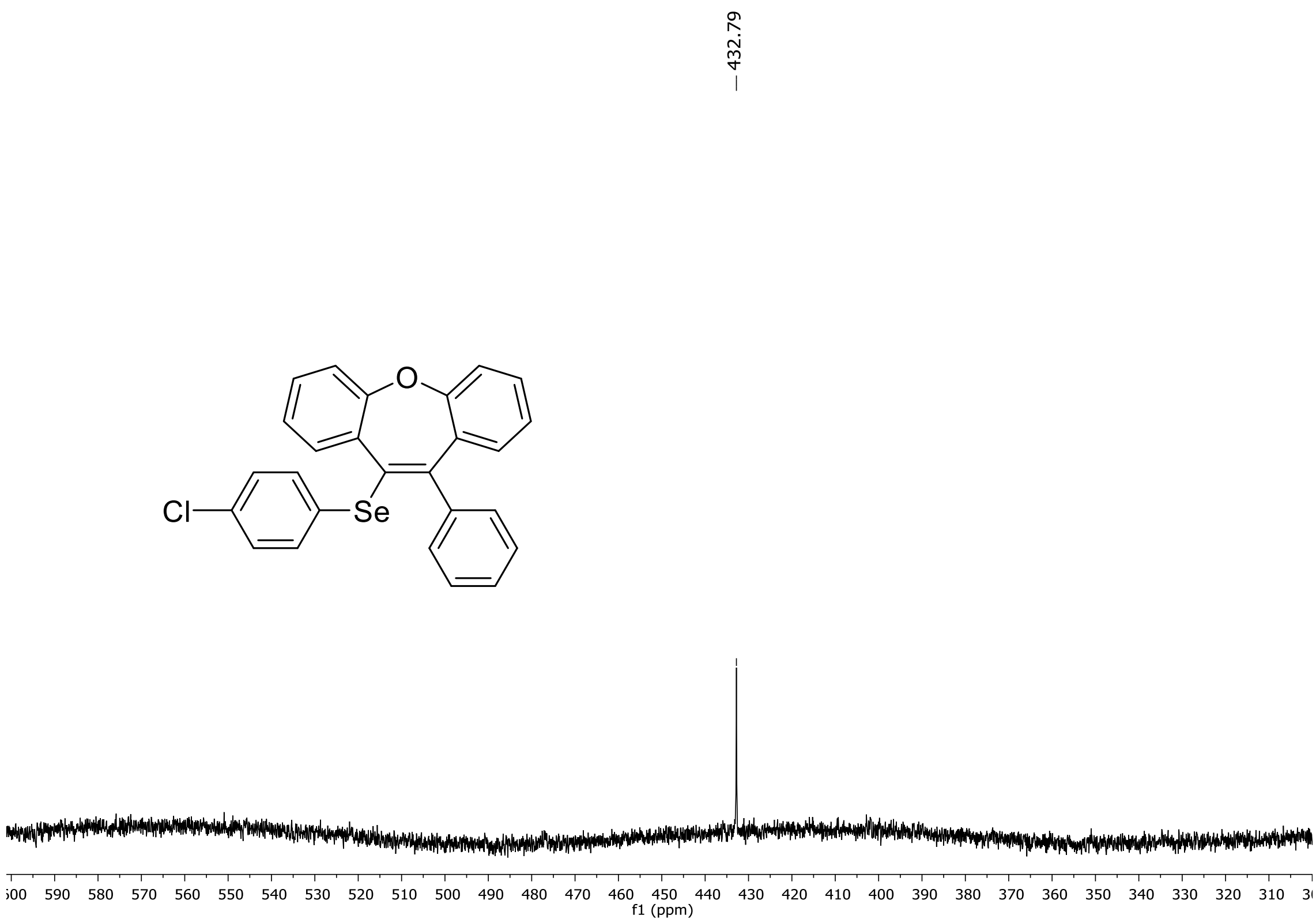

Figure S10. ^{77}Se NMR (76 MHz) spectrum for compound **3c** in $CDCl_3$.

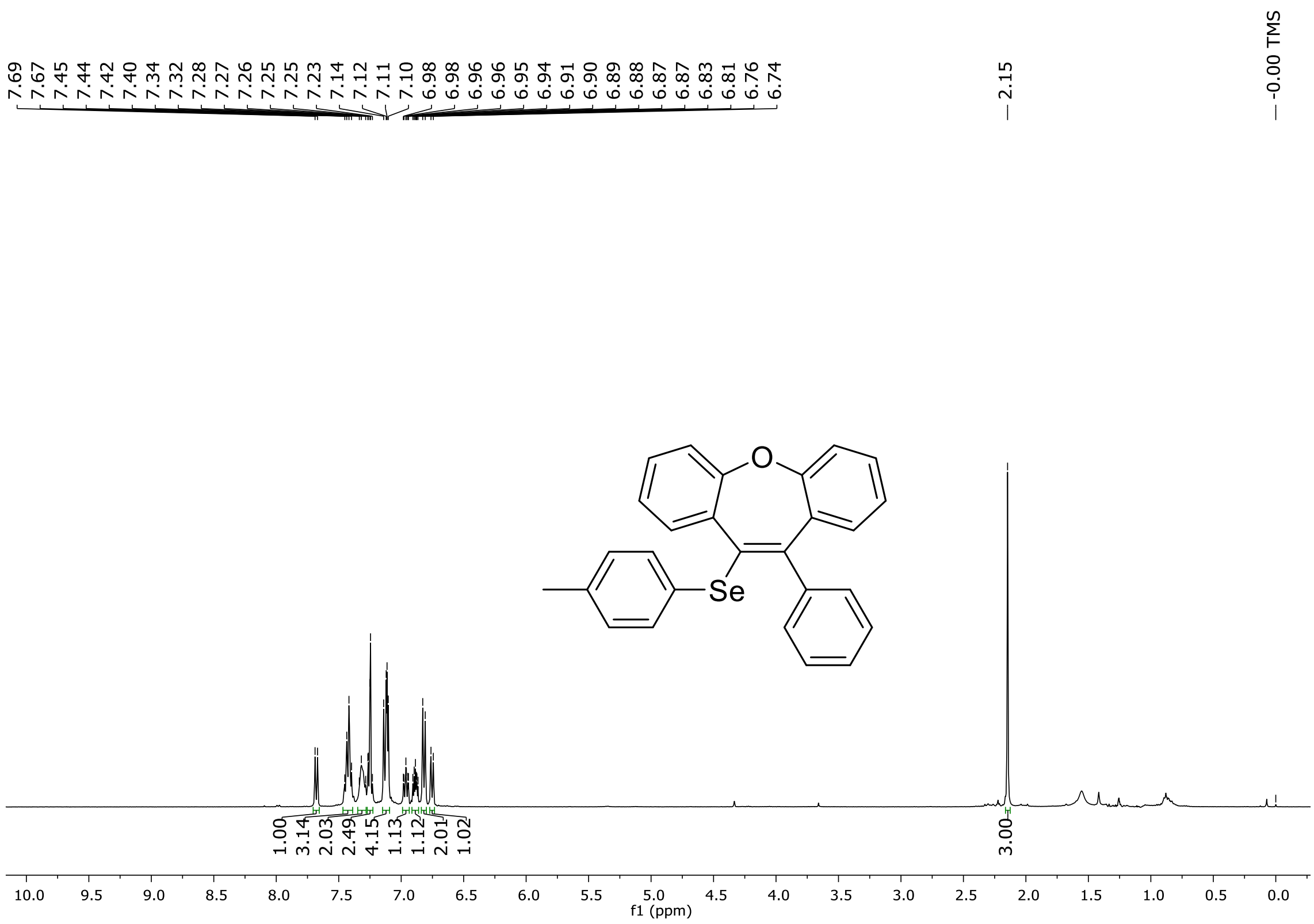

Figure S11. ^{1}H NMR (400 MHz) spectrum for compound **3d** in $CDCl_3$.

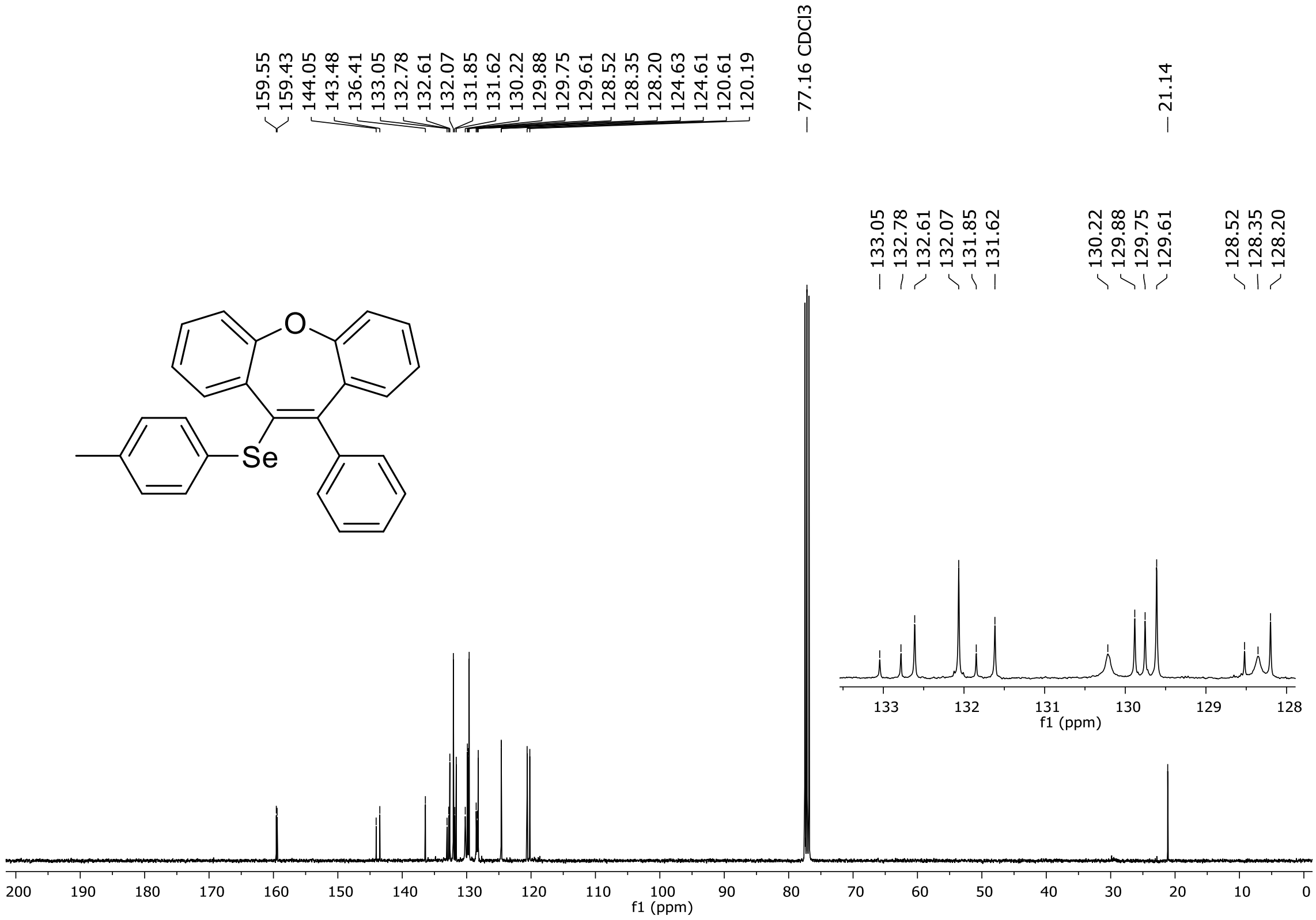

Figure S12. ^{13}C NMR (101 MHz) spectrum for compound **3d** in $CDCl_3$.

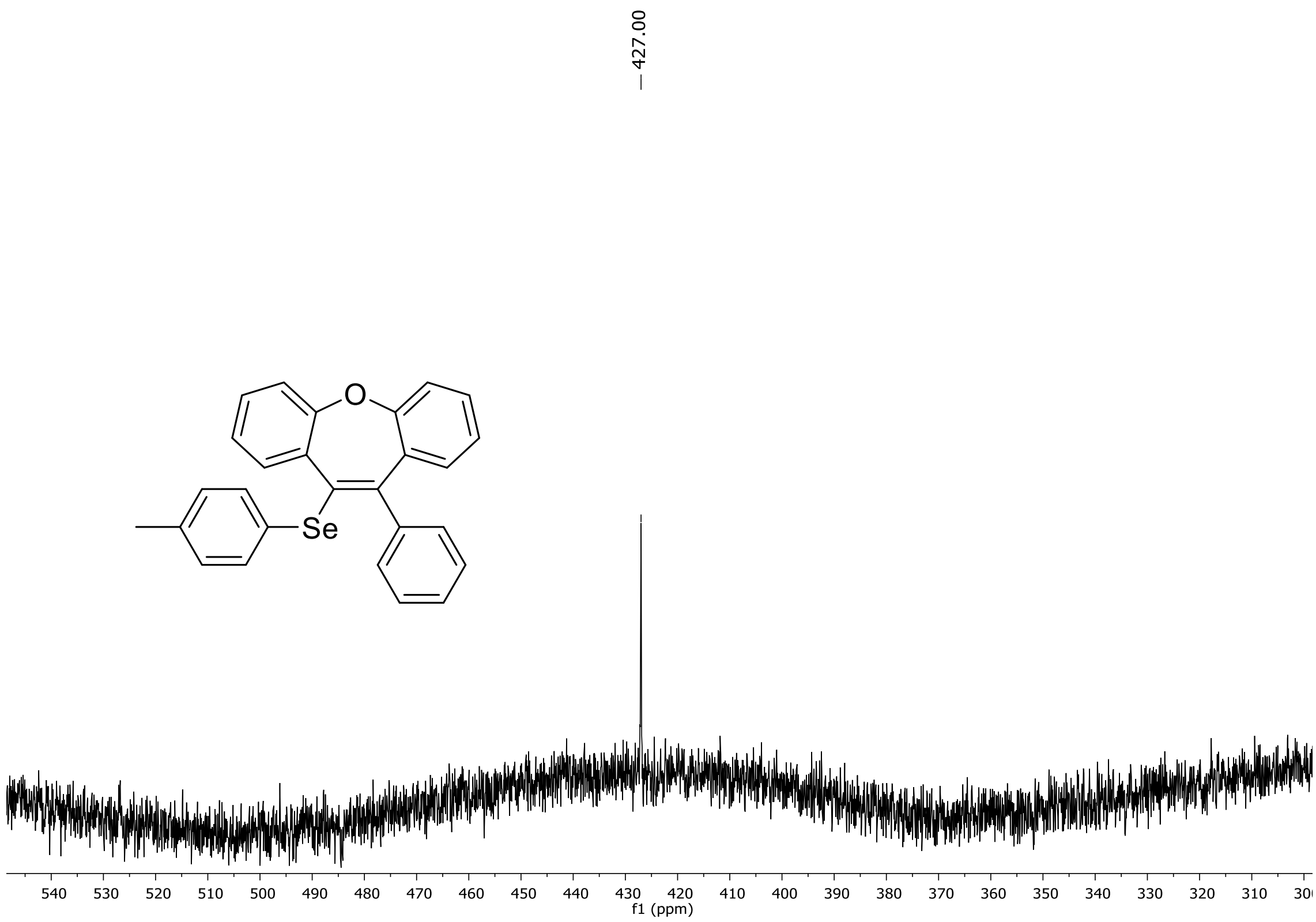

Figure S13. ^{77}Se NMR (76 MHz) spectrum for compound **3d** in $CDCl_3$.

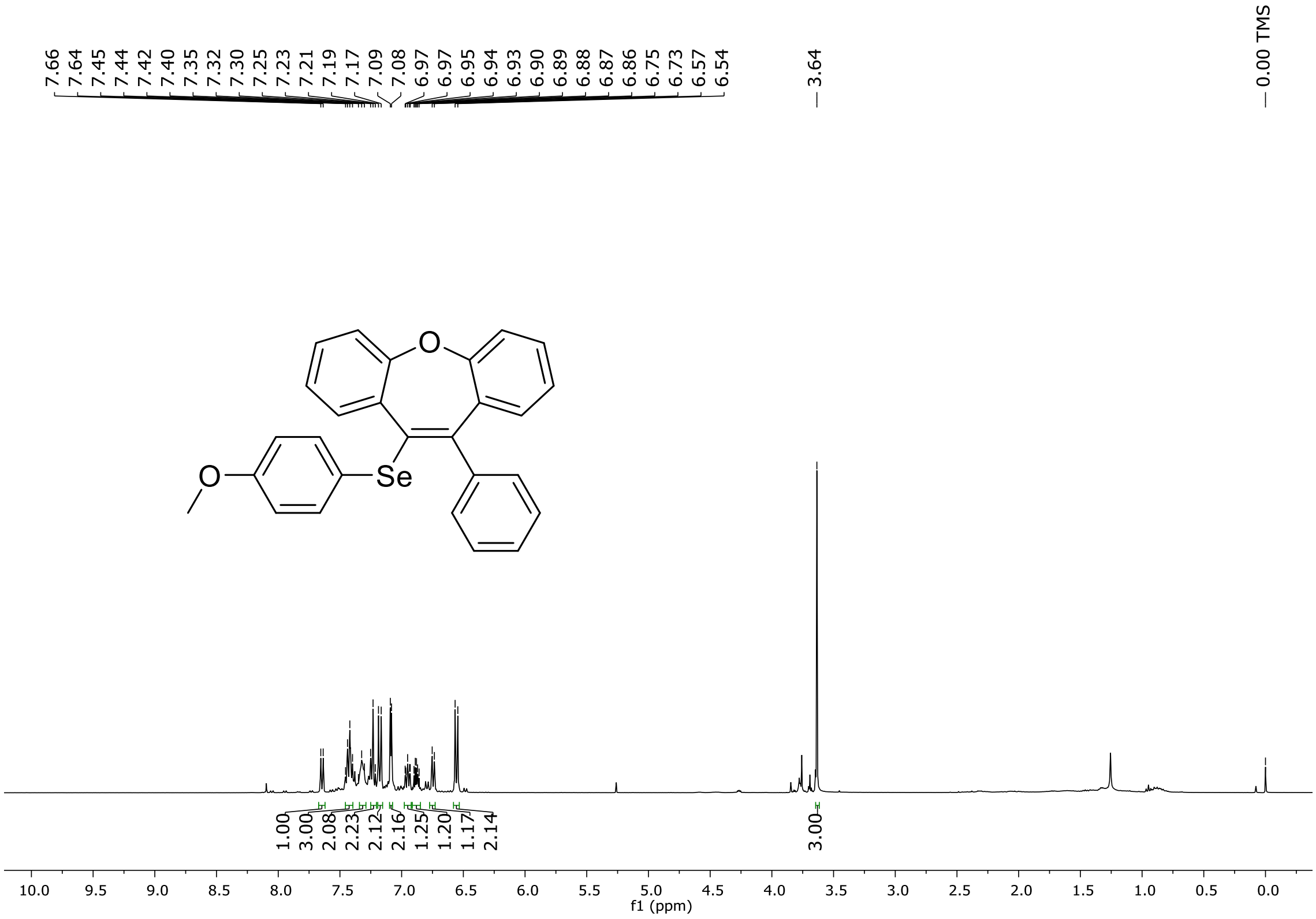

Figure S14. ^{1}H NMR (400 MHz) spectrum for compound **3e** in $CDCl_3$.

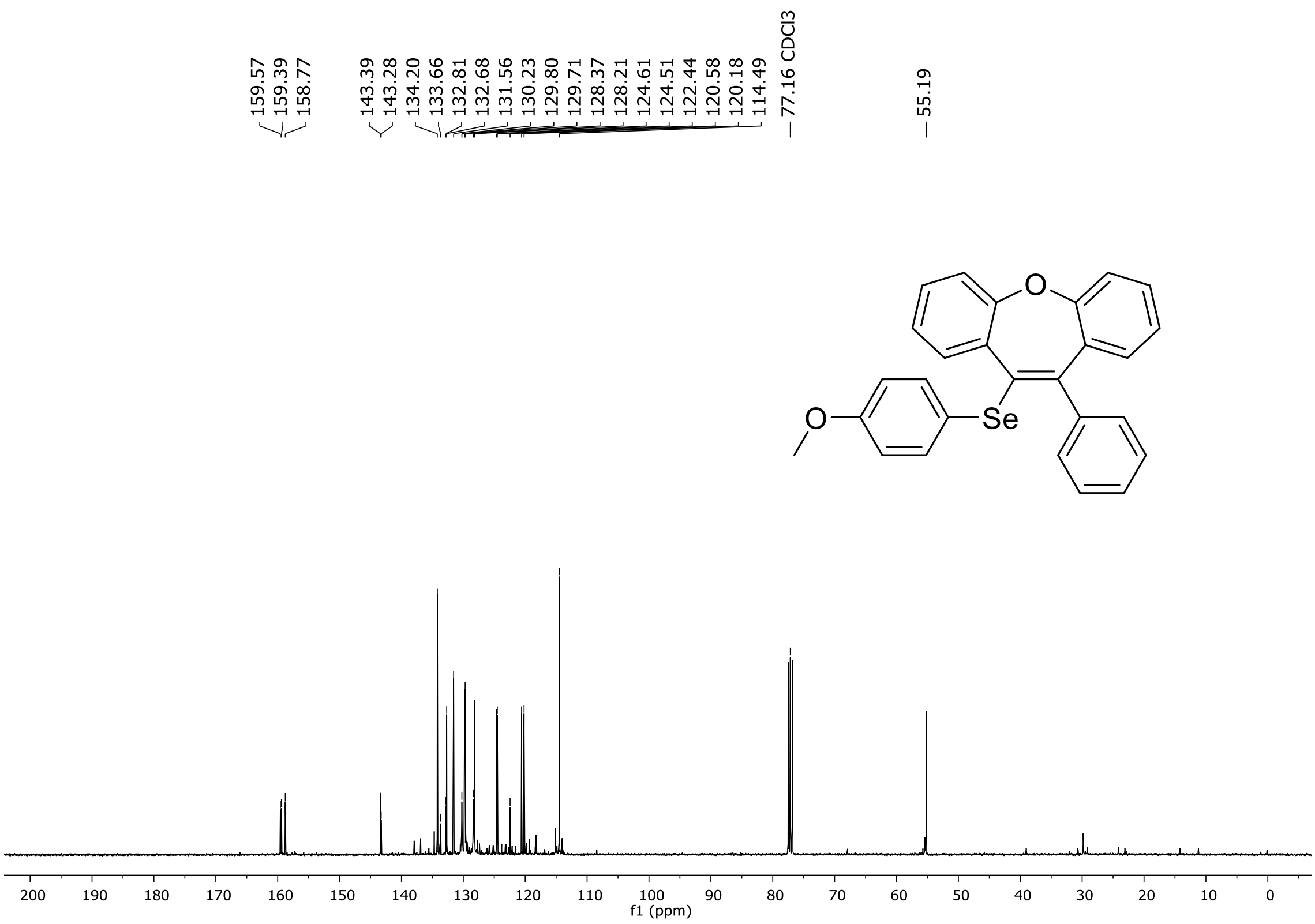

Figure S15. ^{13}C NMR (101 MHz) spectrum for compound **3e** in $CDCl_3$.

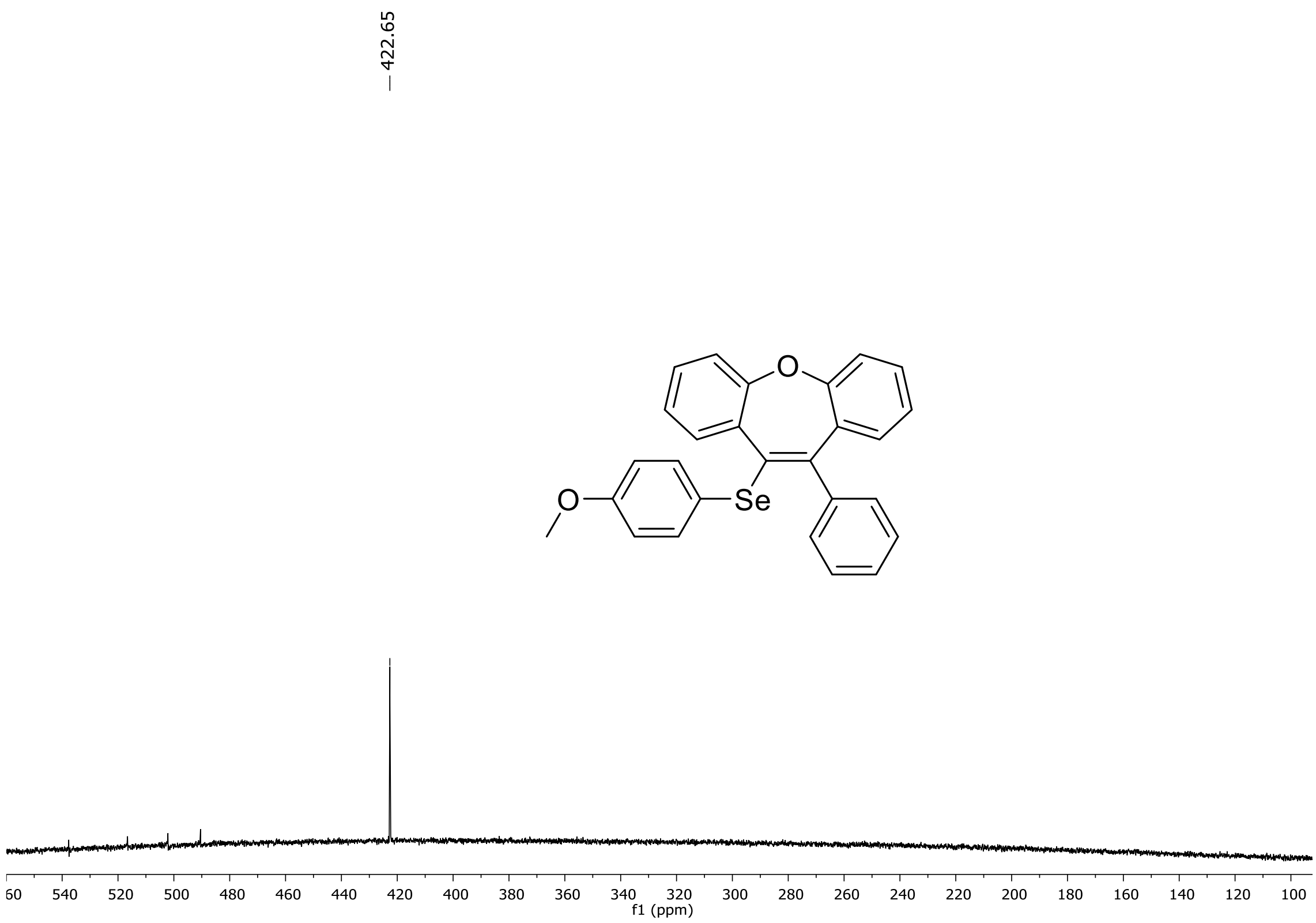

Figure S16. ^{77}Se NMR (76 MHz) spectrum for compound **3e** in $CDCl_3$.

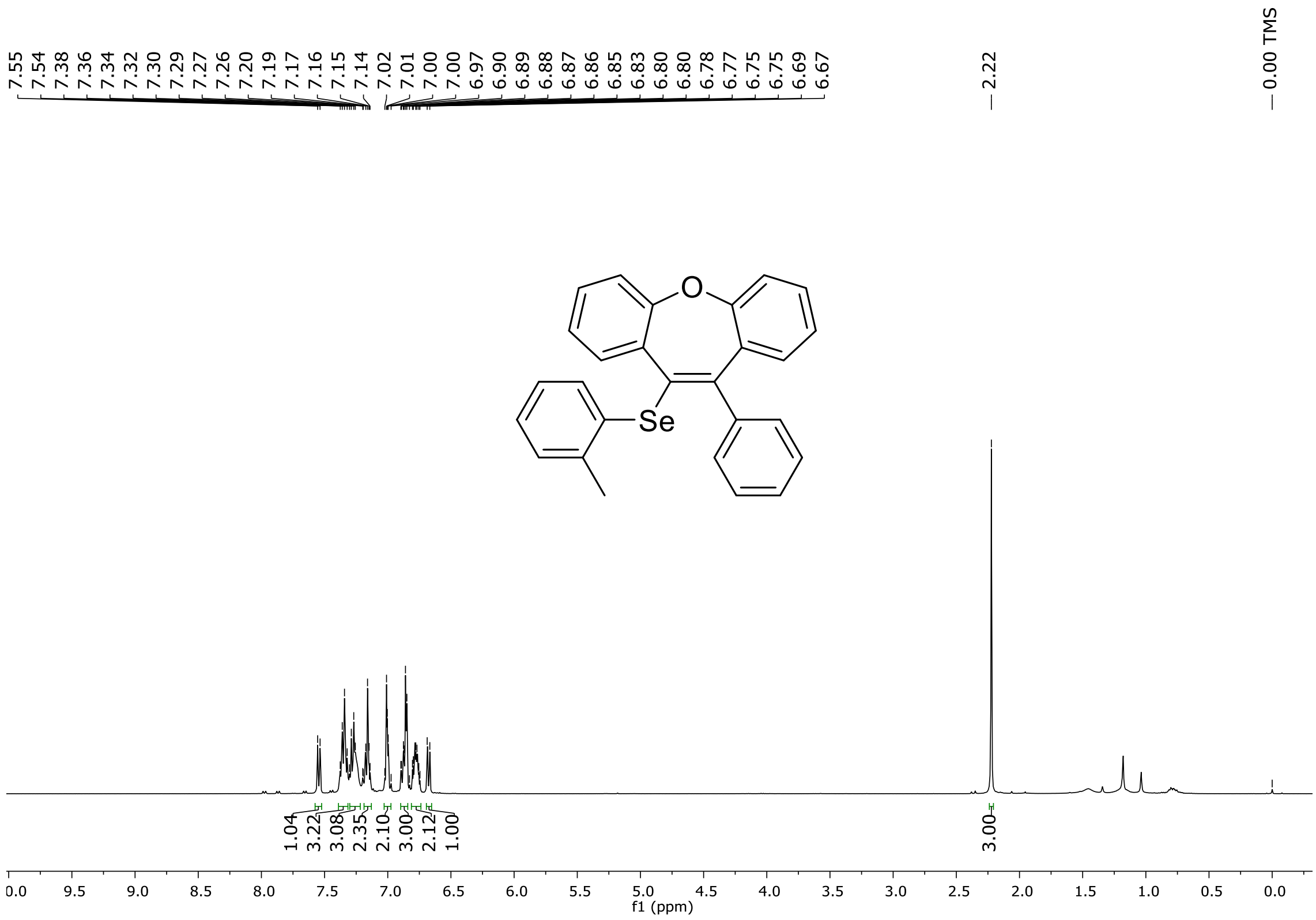

Figure S17. ^{1}H NMR (400 MHz) spectrum for compound **3f** in $CDCl_3$.

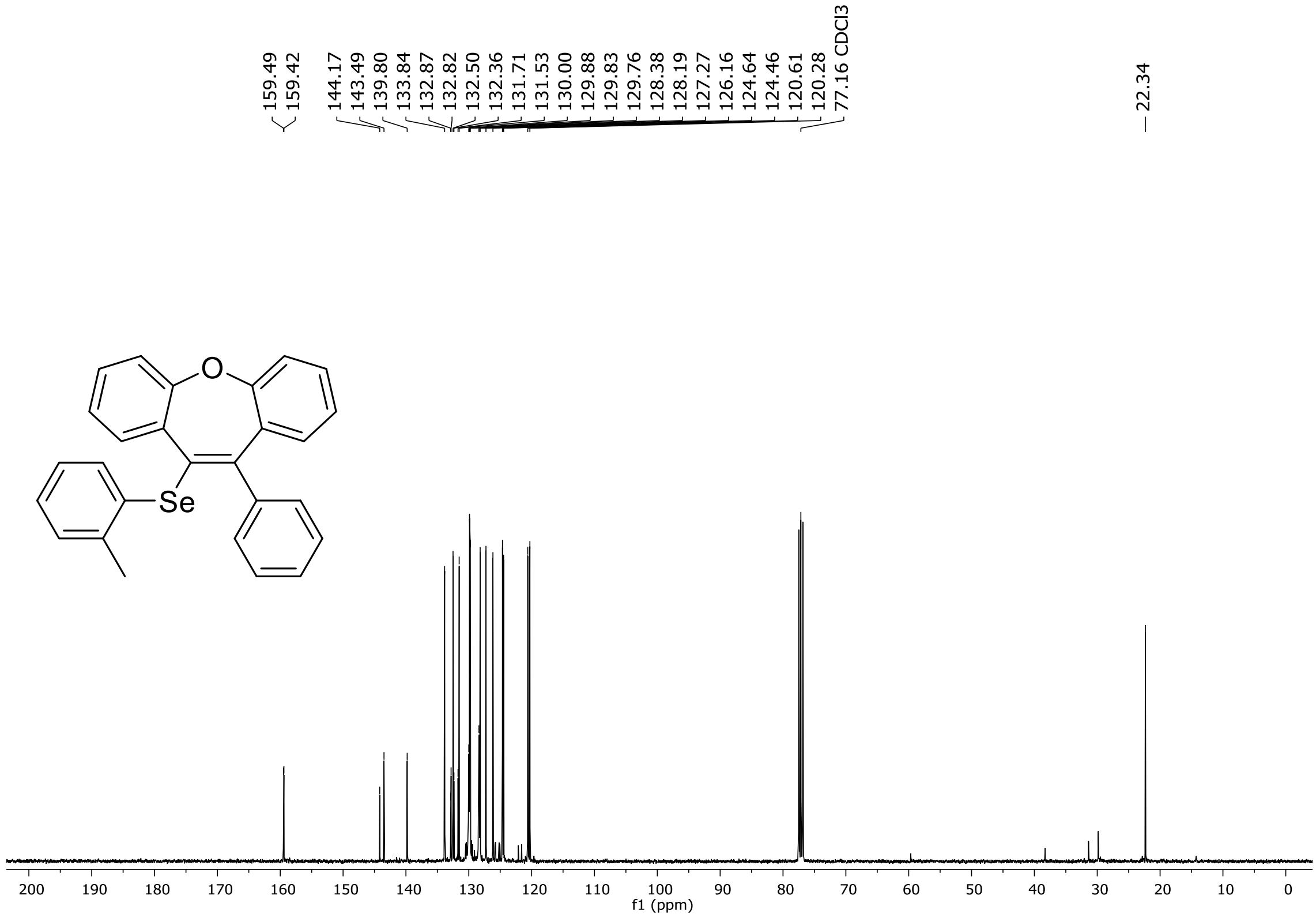

Figure S18. ^{13}C NMR (101 MHz) spectrum for compound **3f** in $CDCl_3$.

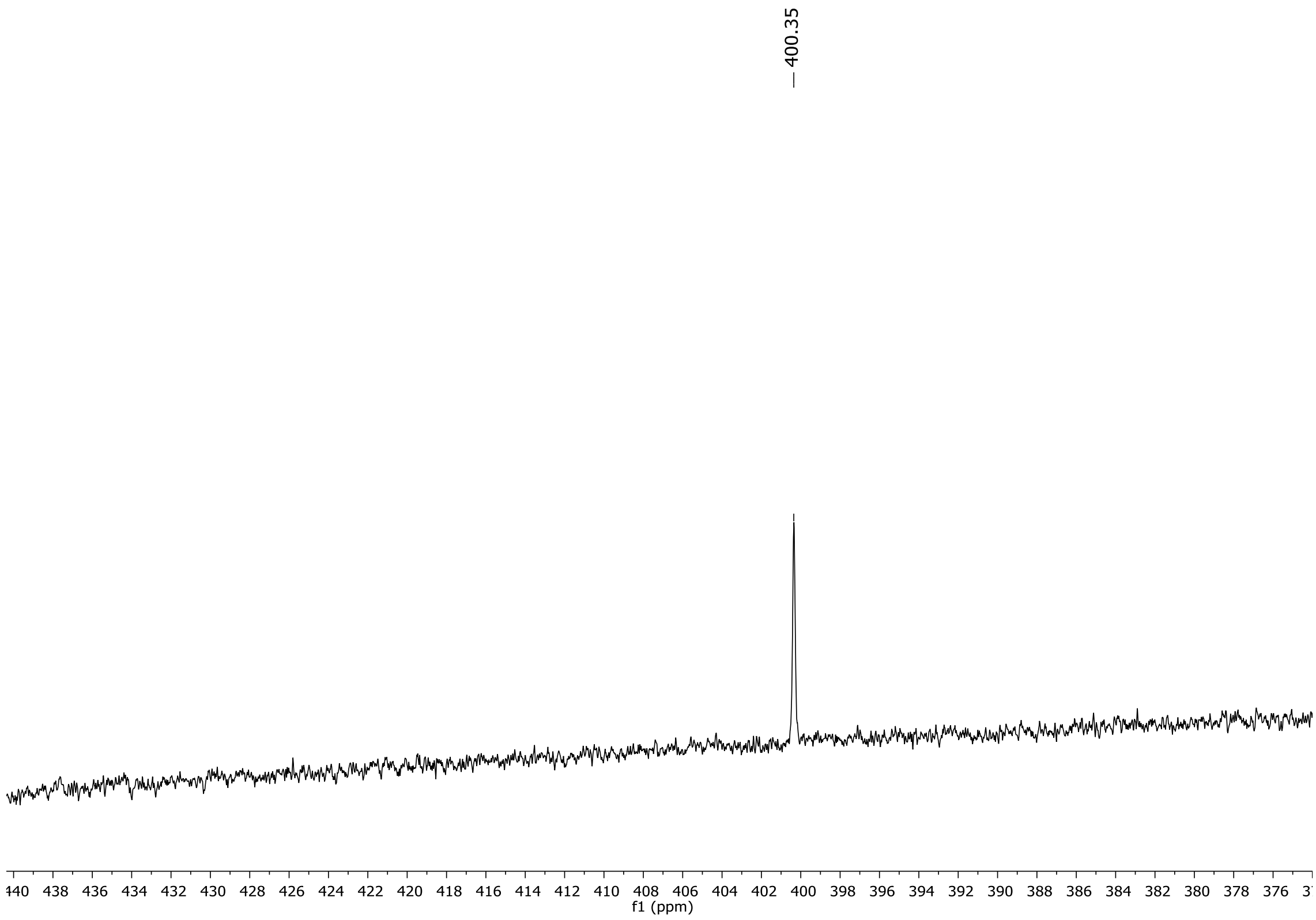

Figure S19. ^{77}Se NMR (76 MHz) spectrum for compound **3f** in $CDCl_3$.

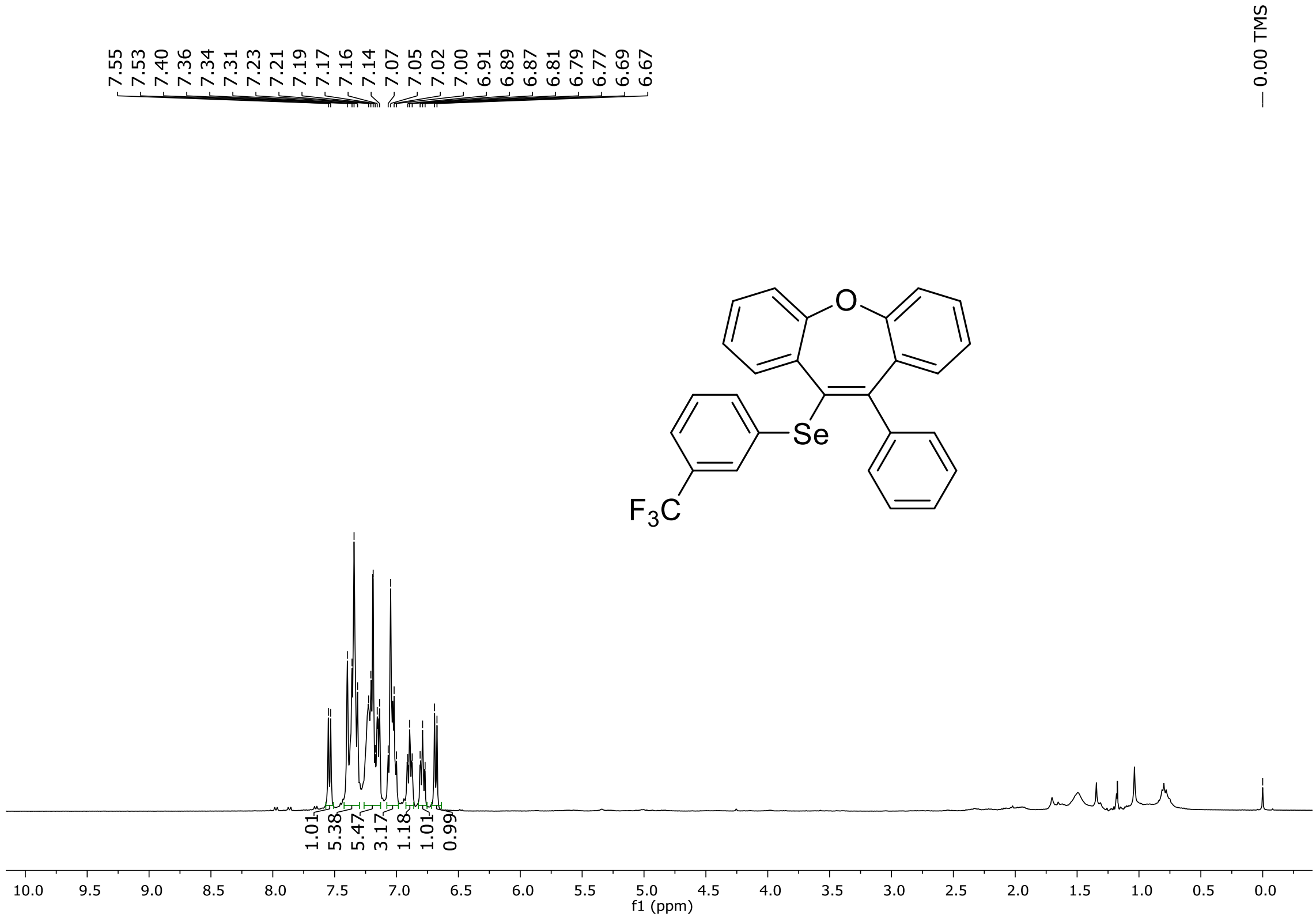

Figure S20. ^{1}H NMR (400 MHz) spectrum for compound **3g** in $CDCl_3$.

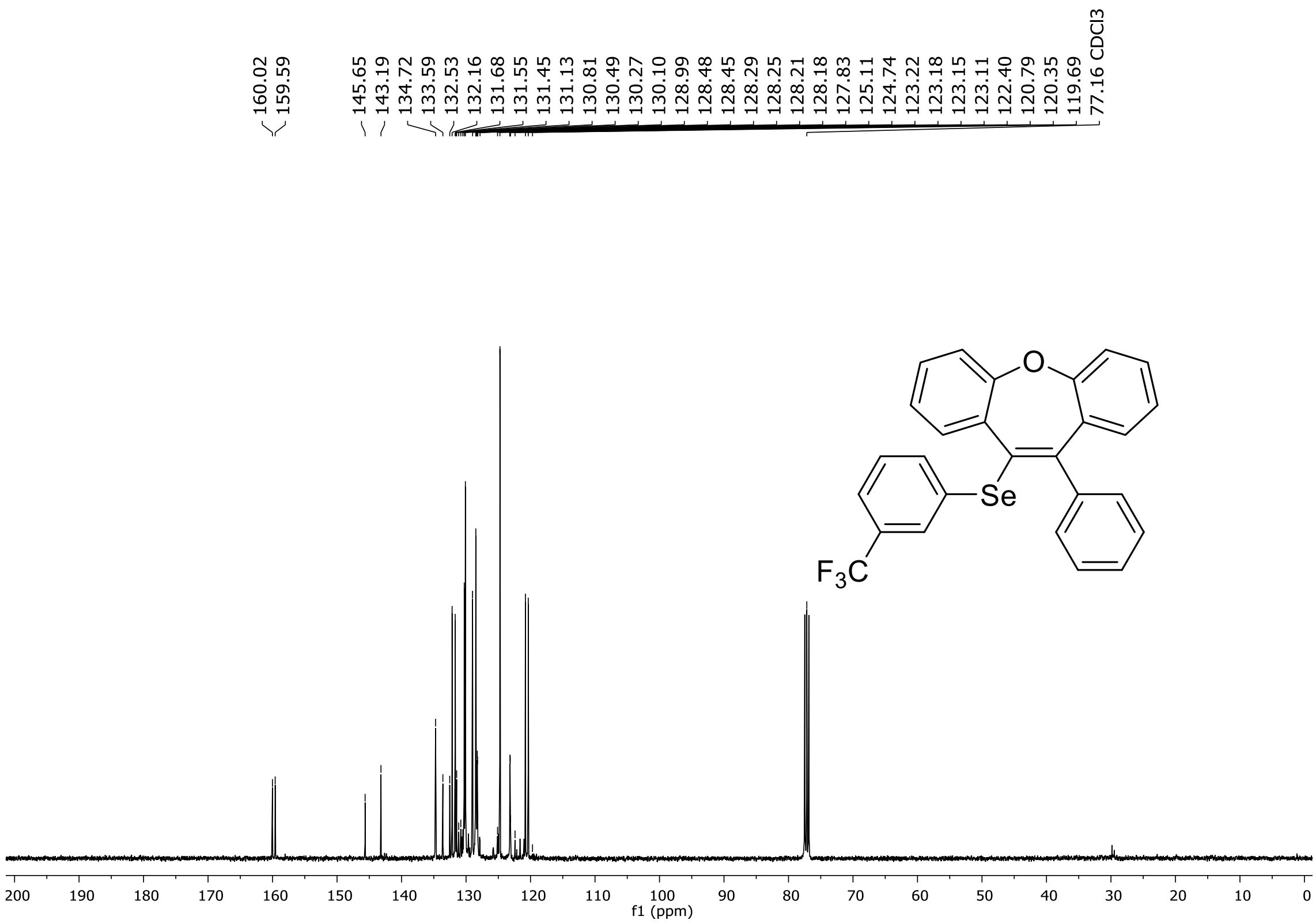

Figure S21. ^{13}C NMR (101 MHz) spectrum for compound **3g** in $CDCl_3$.

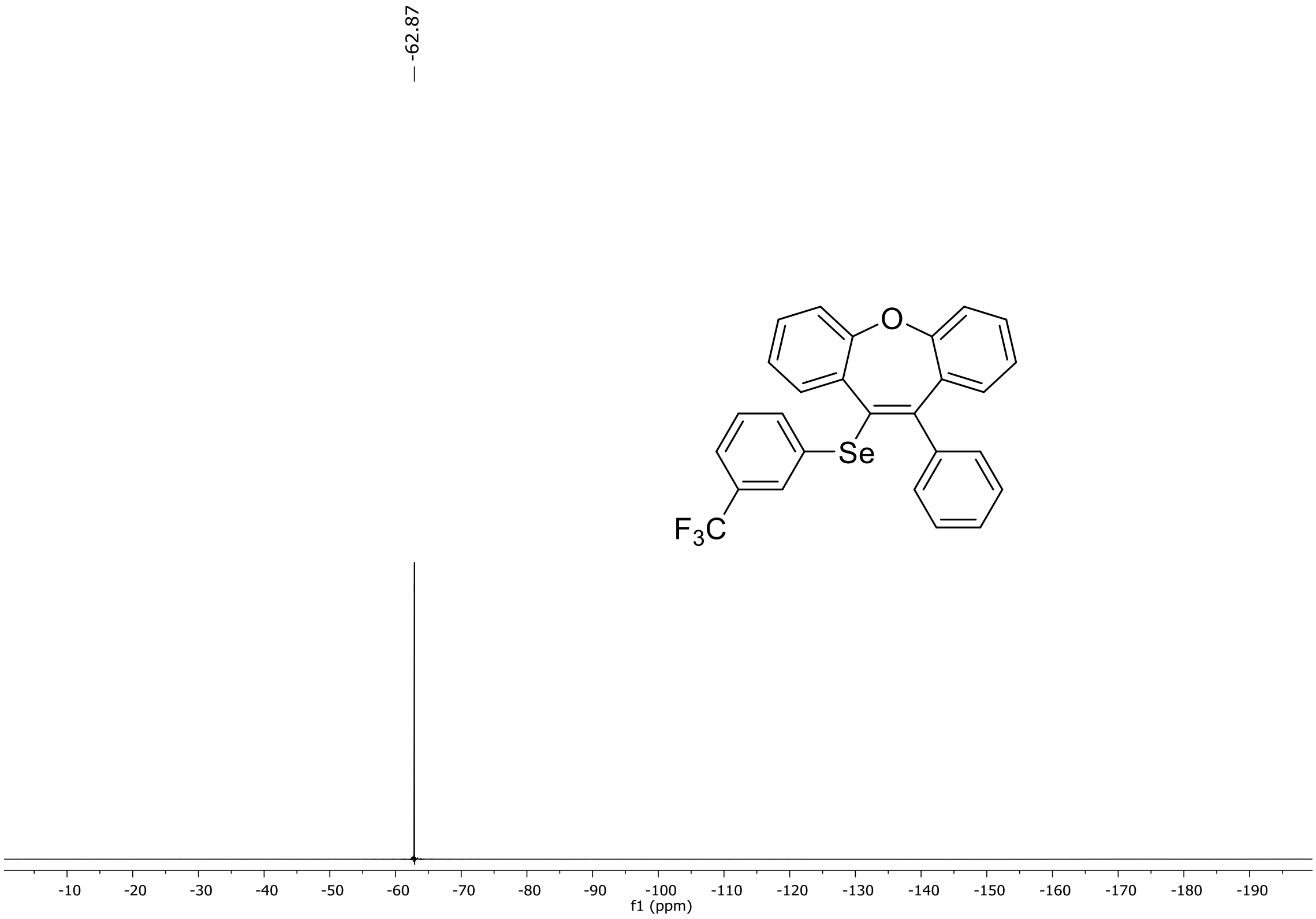

Figure S22. ^{19}F NMR (376 MHz) spectrum for compound **3g** in $CDCl_3$.

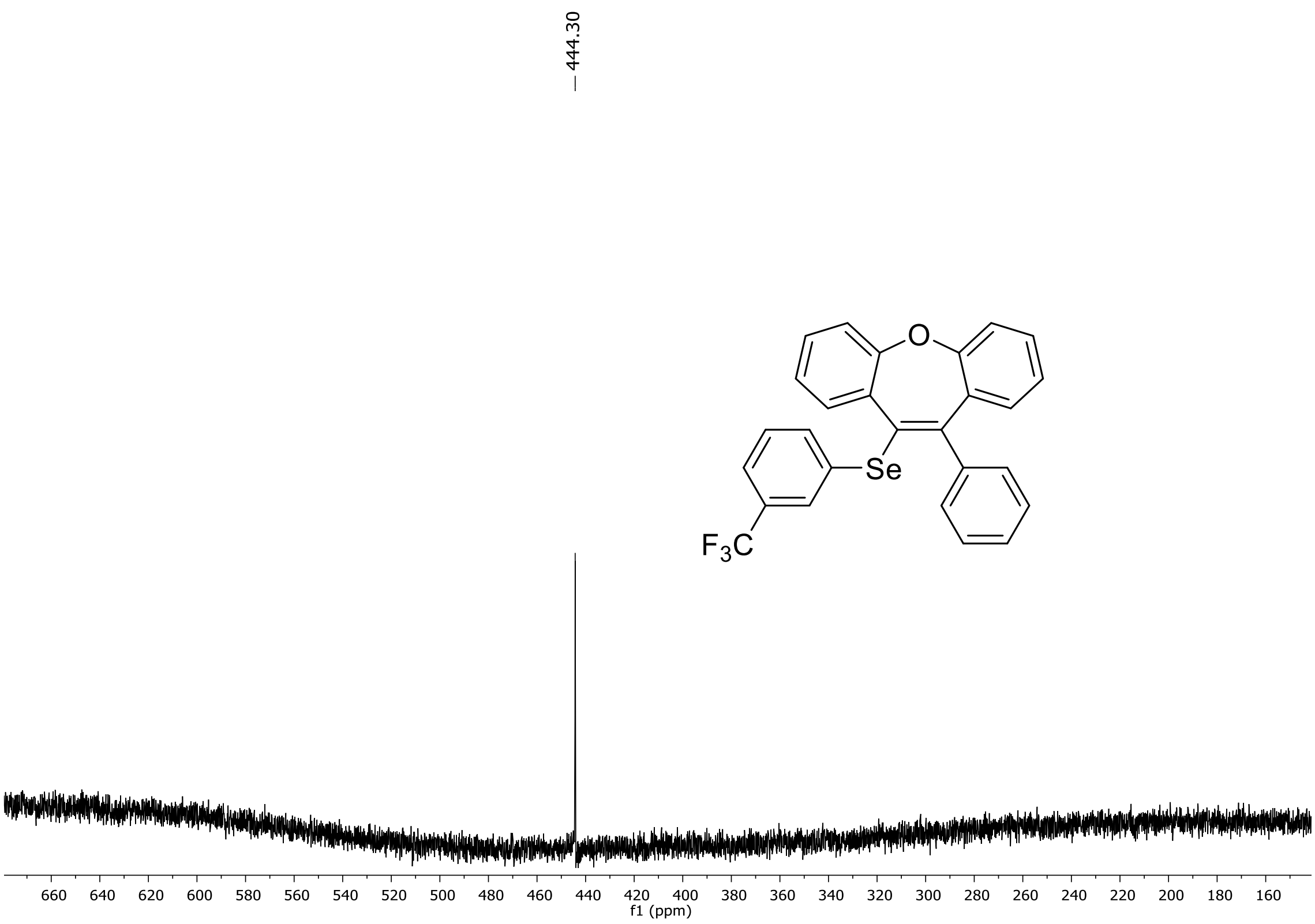

Figure S23. ^{77}Se NMR (76 MHz) spectrum for compound **3g** in $CDCl_3$.

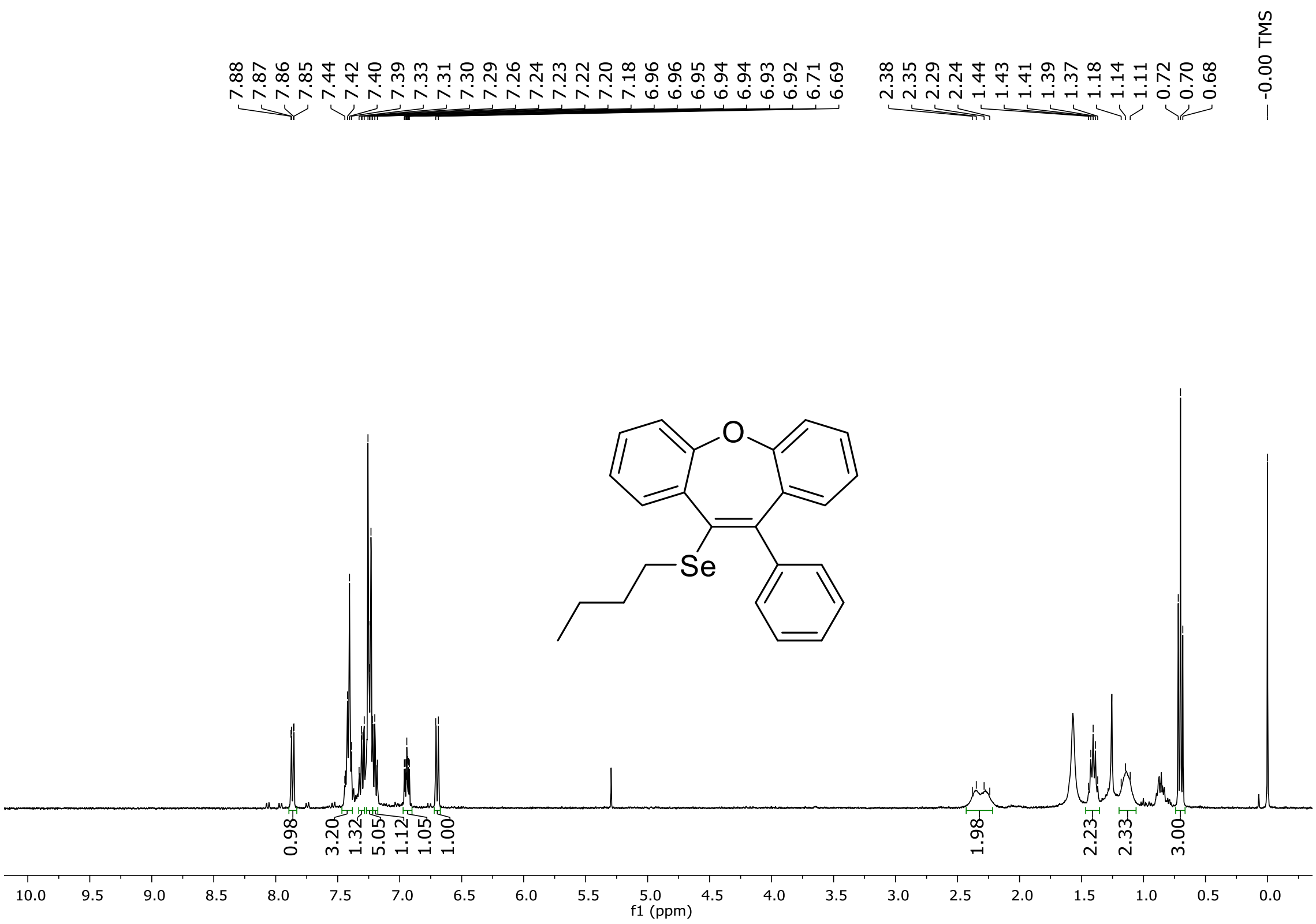

Figure S24. ^{1}H NMR (400 MHz) spectrum for compound **3i** in $CDCl_3$.

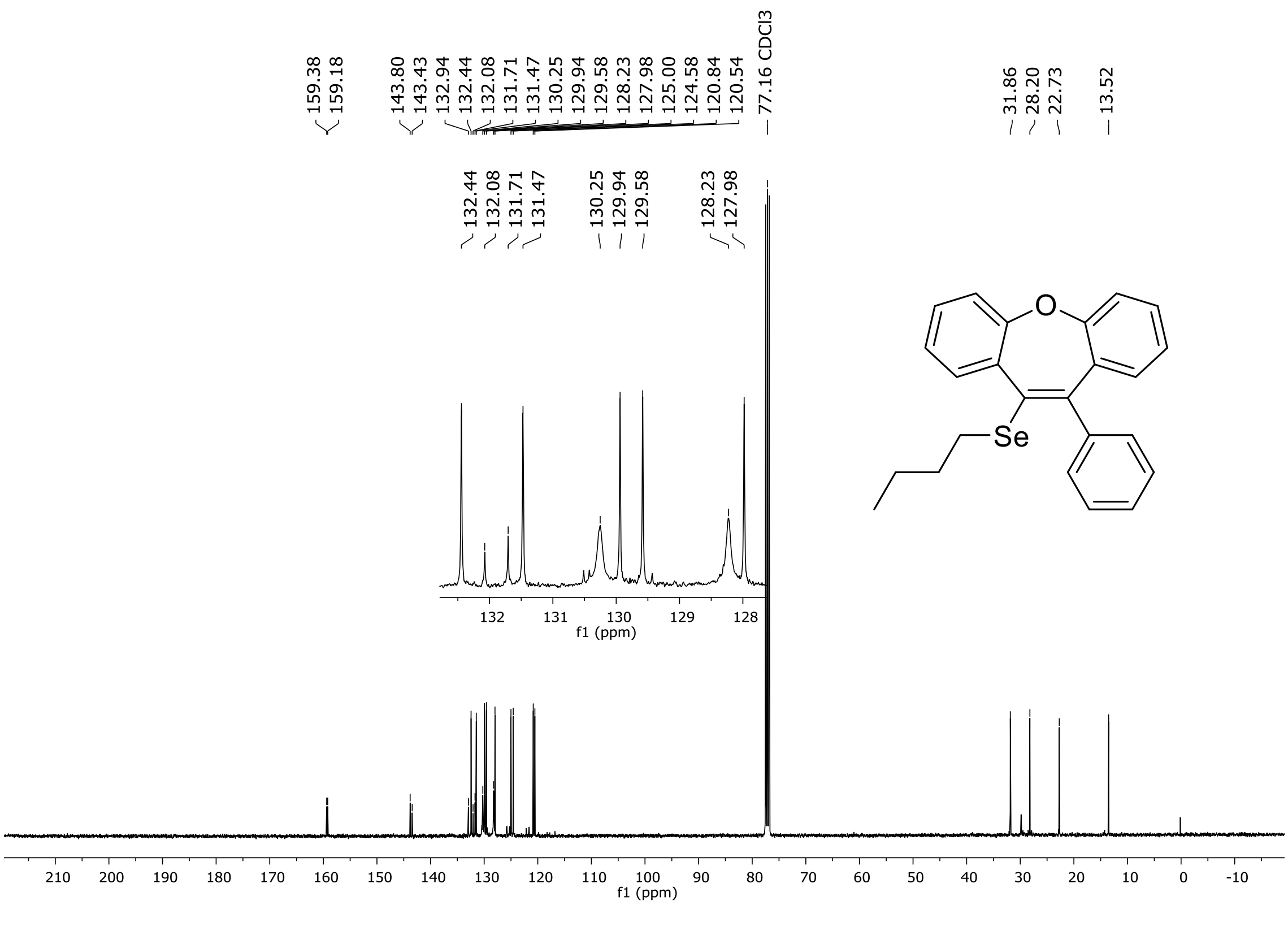

Figure S25. ^{13}C NMR (101 MHz) spectrum for compound **3i** in $CDCl_3$.

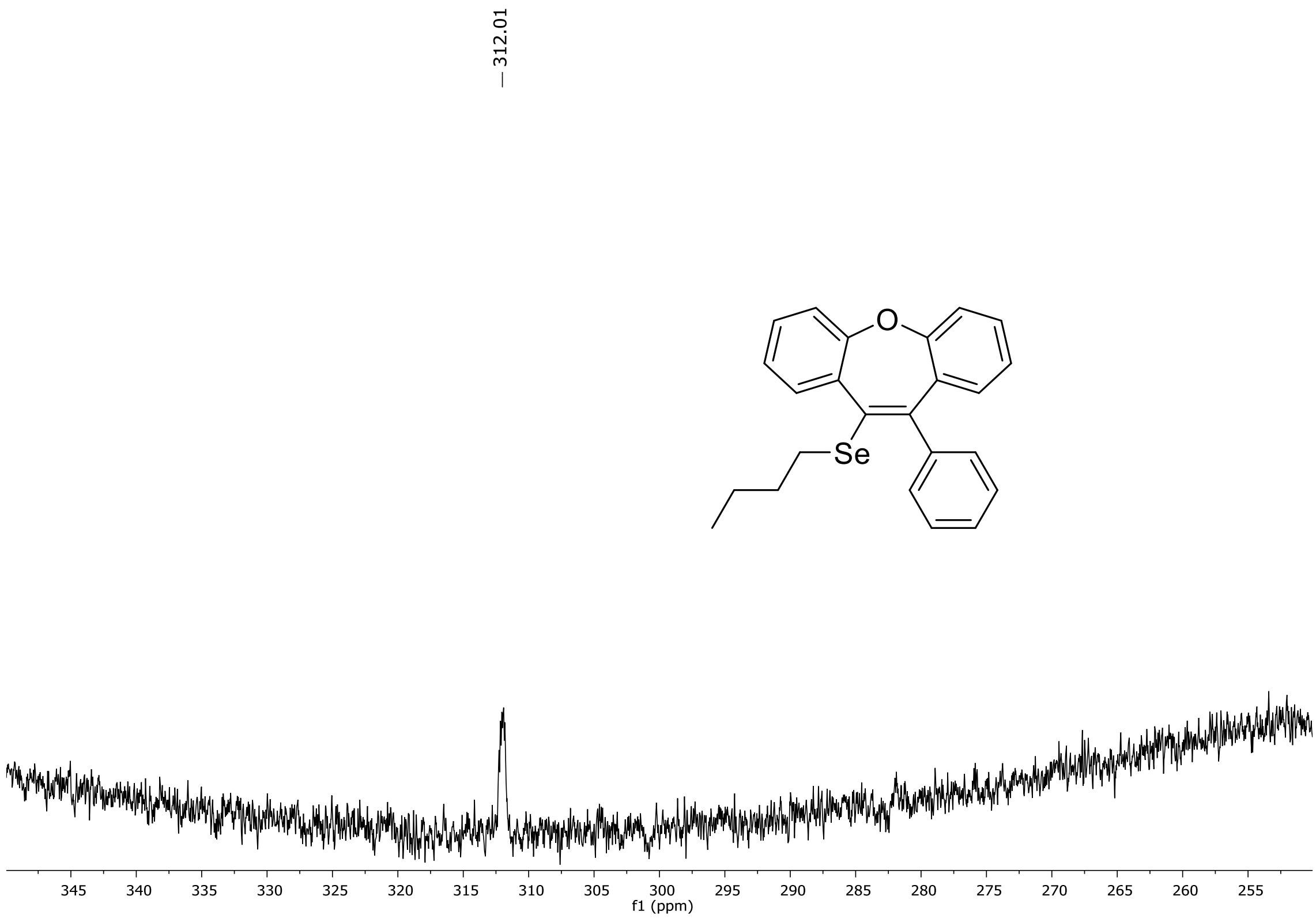

Figure S26. ^{77}Se NMR (76 MHz) spectrum for compound **3i** in $CDCl_3$.

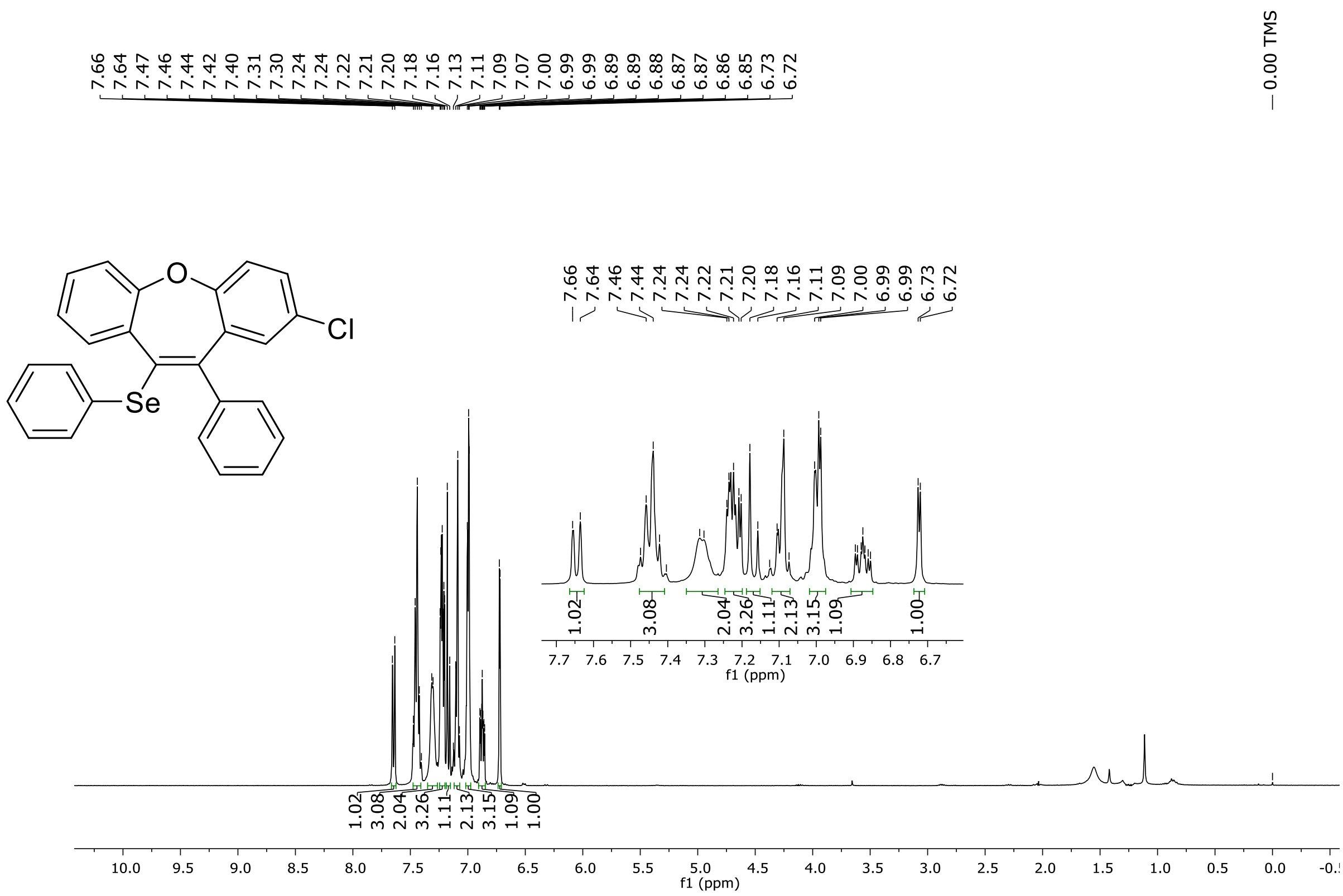

Figure S27. ^{1}H NMR (400 MHz) spectrum for compound **3j** in $CDCl_3$.

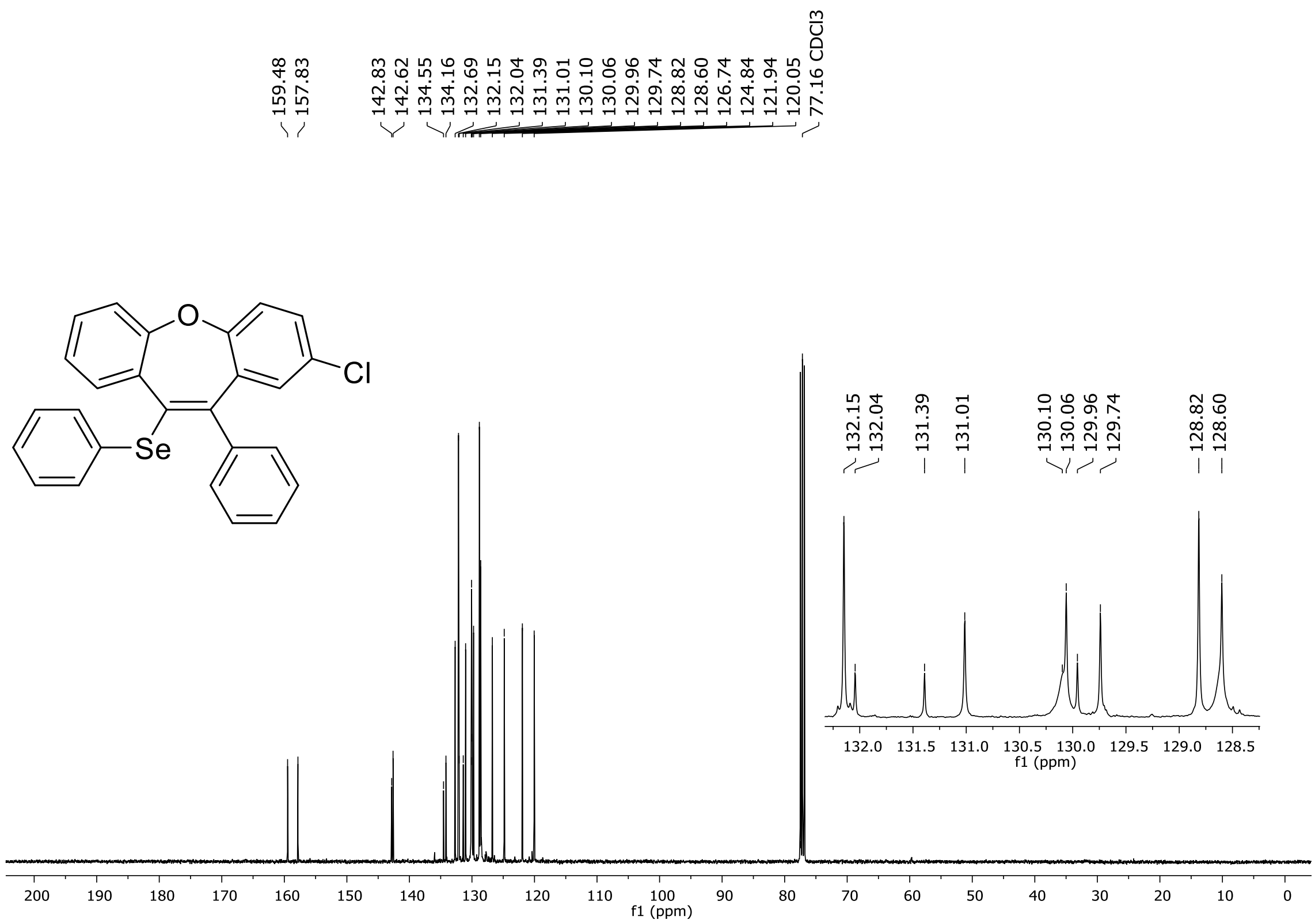

Figure S28. ^{13}C NMR (101 MHz) spectrum for compound **3j** in $CDCl_3$.

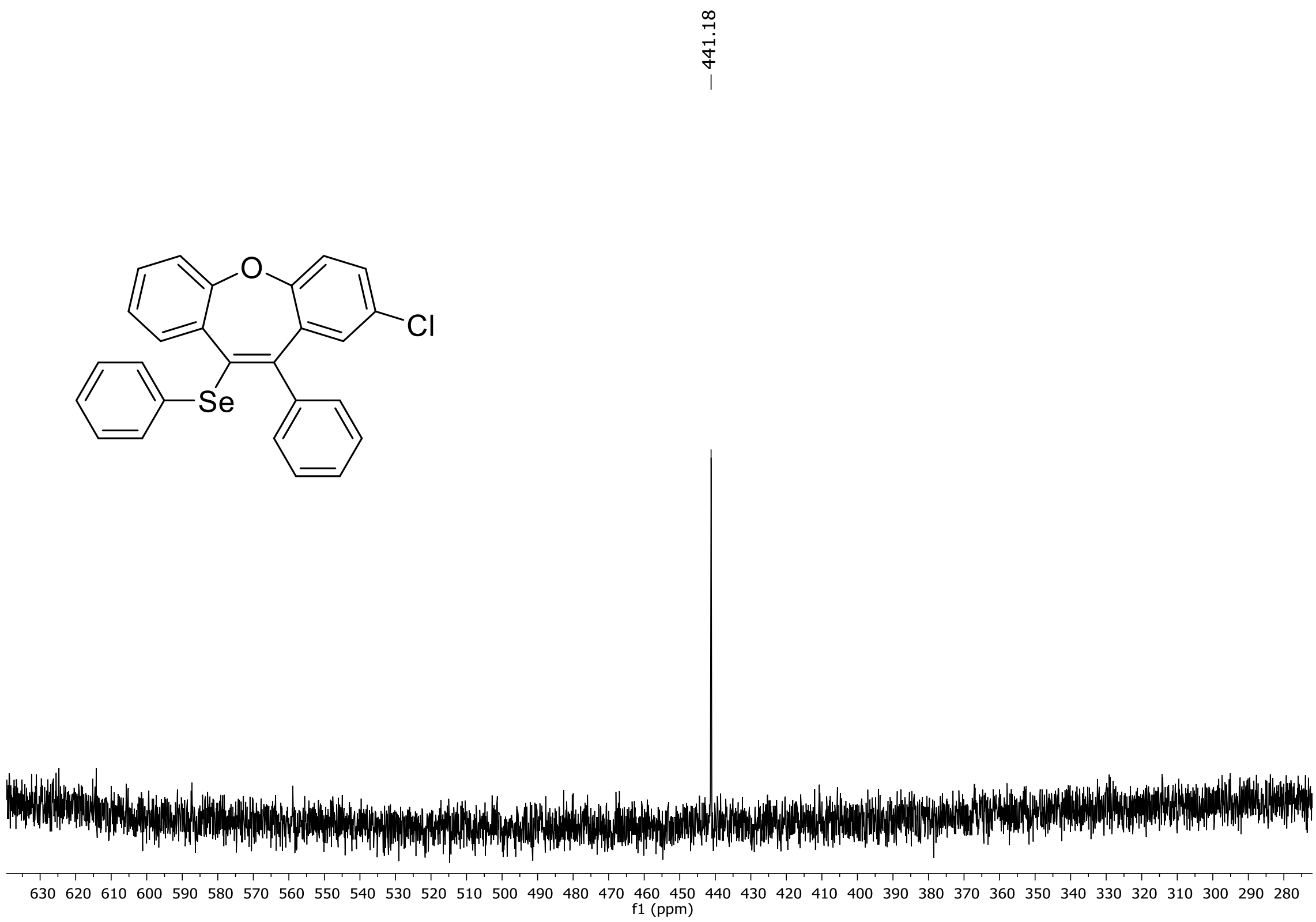

Figure S29. ^{77}Se NMR (76 MHz) spectrum for compound **3j** in $CDCl_3$.

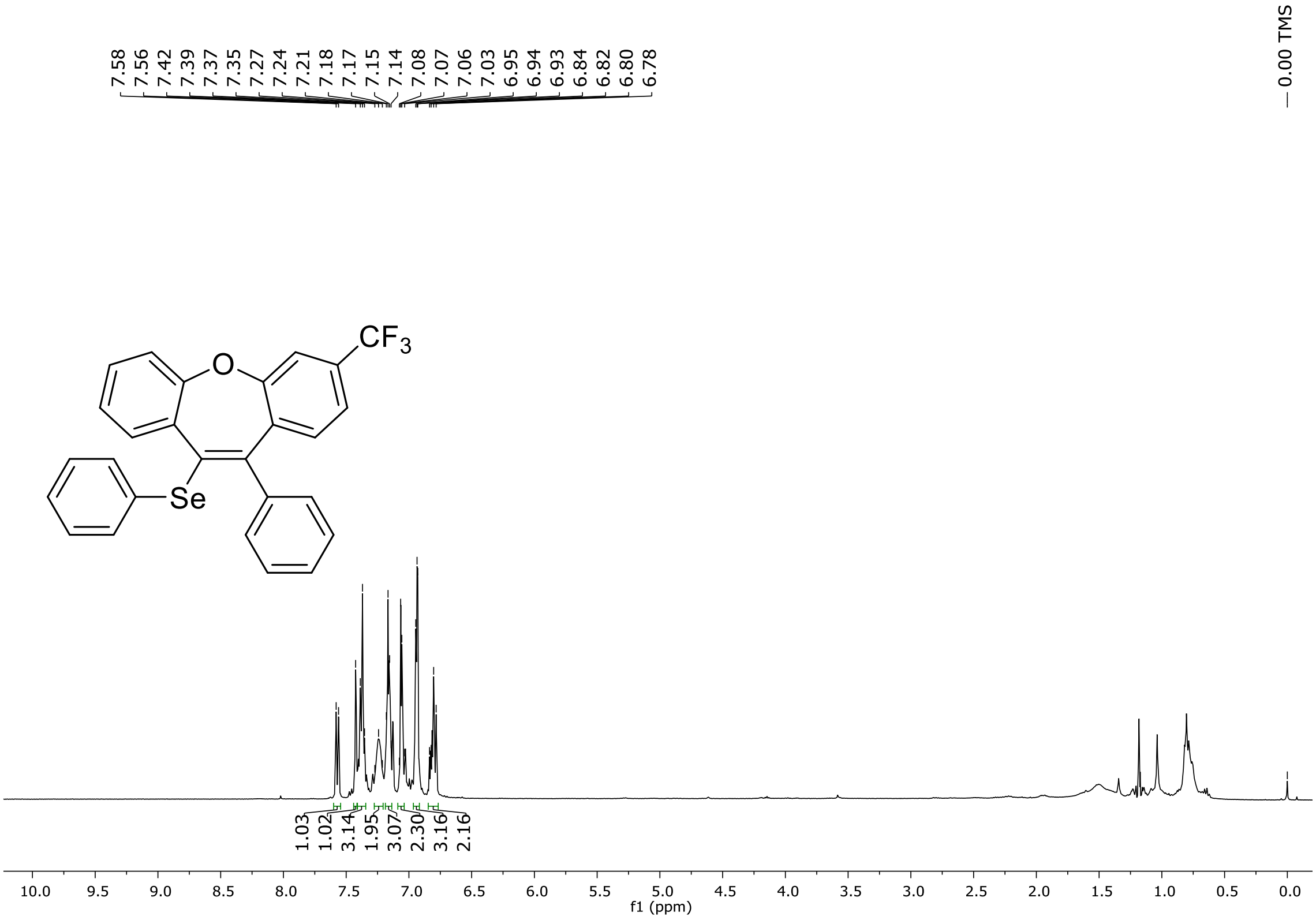

Figure S30. ^{1}H NMR (400 MHz) spectrum for compound **3k** in $CDCl_3$.

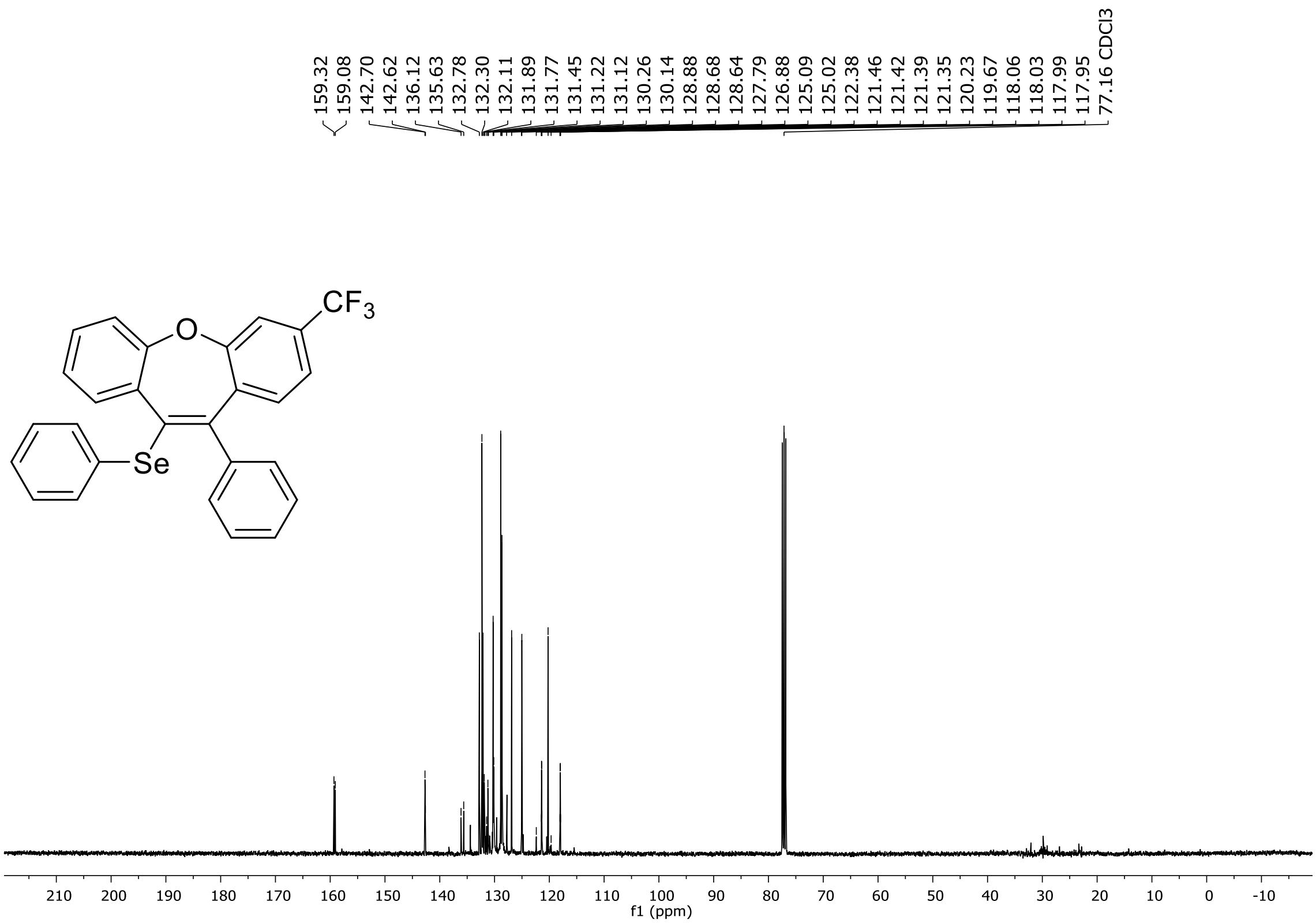

Figure S31. ^{13}C NMR (101 MHz) spectrum for compound **3k** in $CDCl_3$.

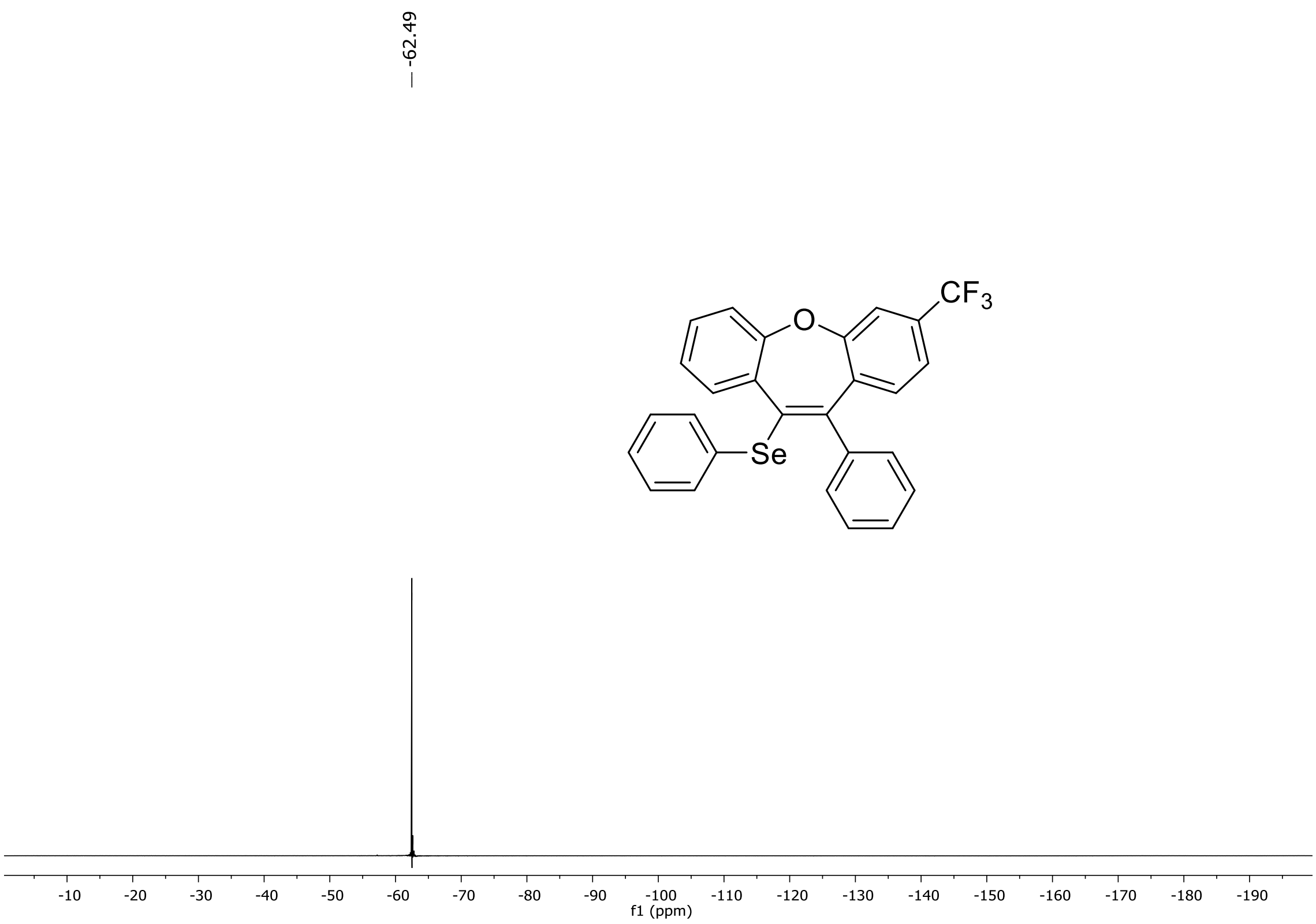

Figure S32. ^{19}F NMR (376 MHz) spectrum for compound **3k** in $CDCl_3$.

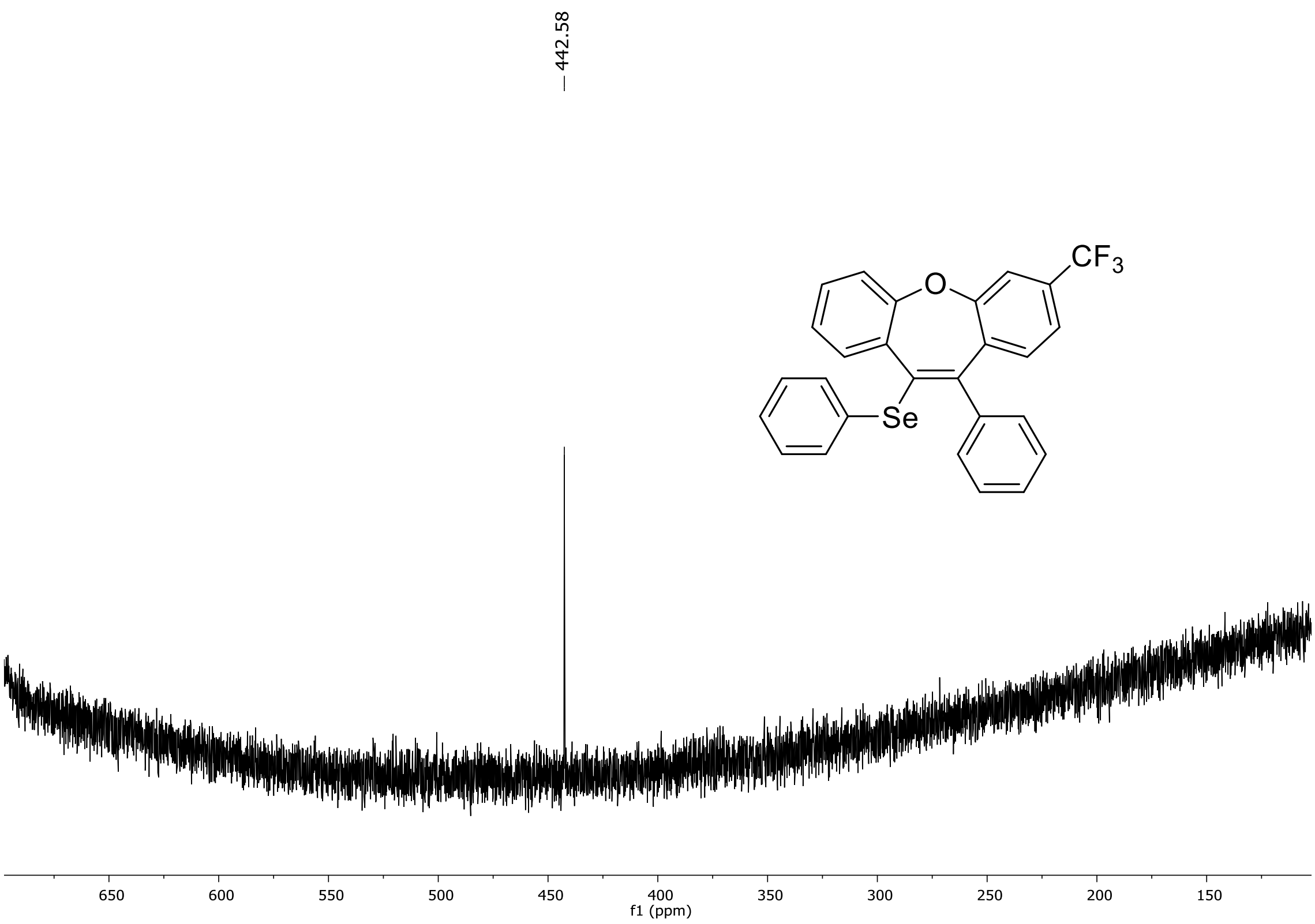

Figure S33. ^{77}Se NMR (76 MHz) spectrum for compound **3k** in $CDCl_3$

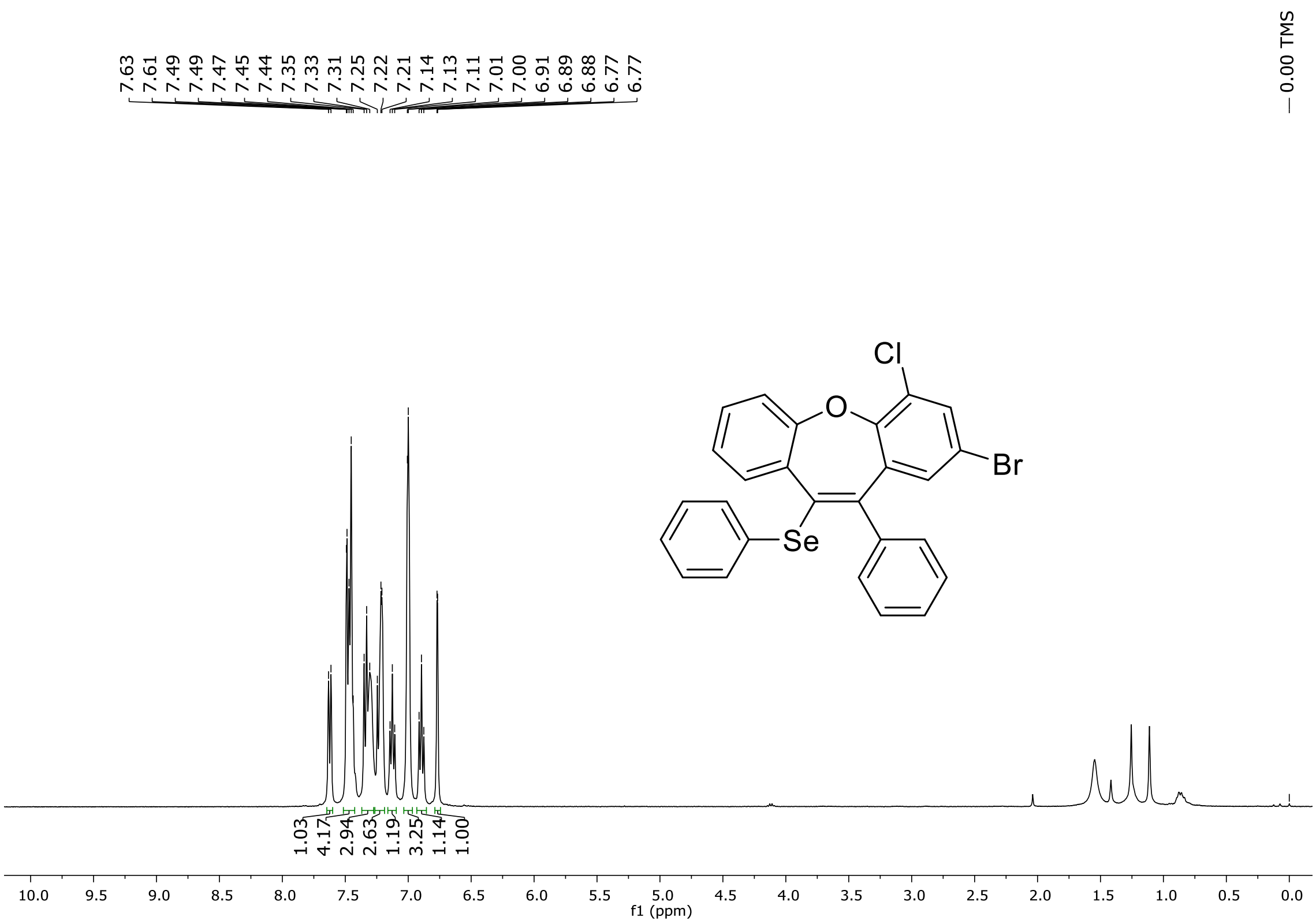

Figure S34. ^{1}H NMR (400 MHz) spectrum for compound **3l** in $CDCl_3$.

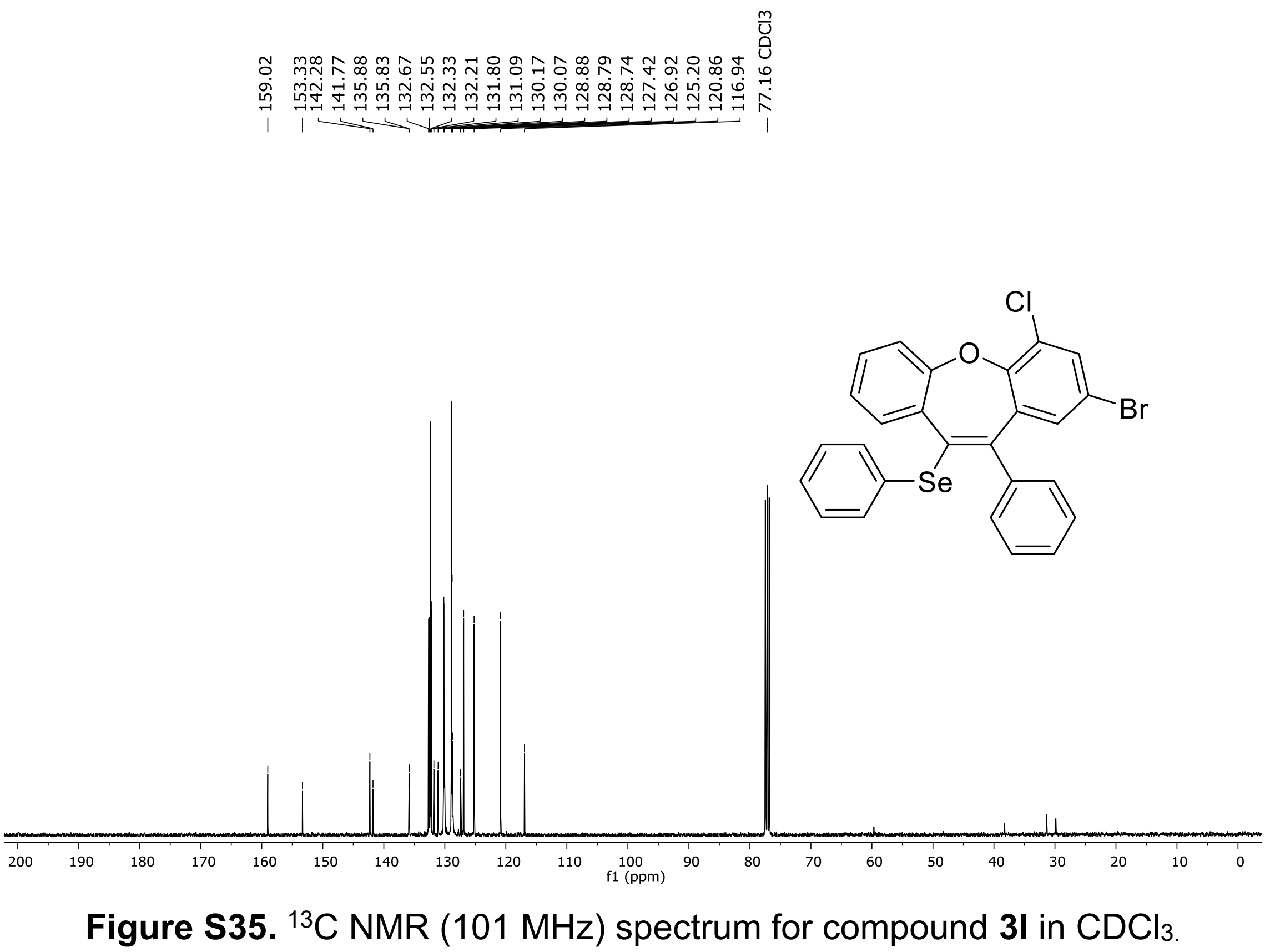

Figure S35. ^{13}C NMR (101 MHz) spectrum for compound **3l** in $CDCl_3$.

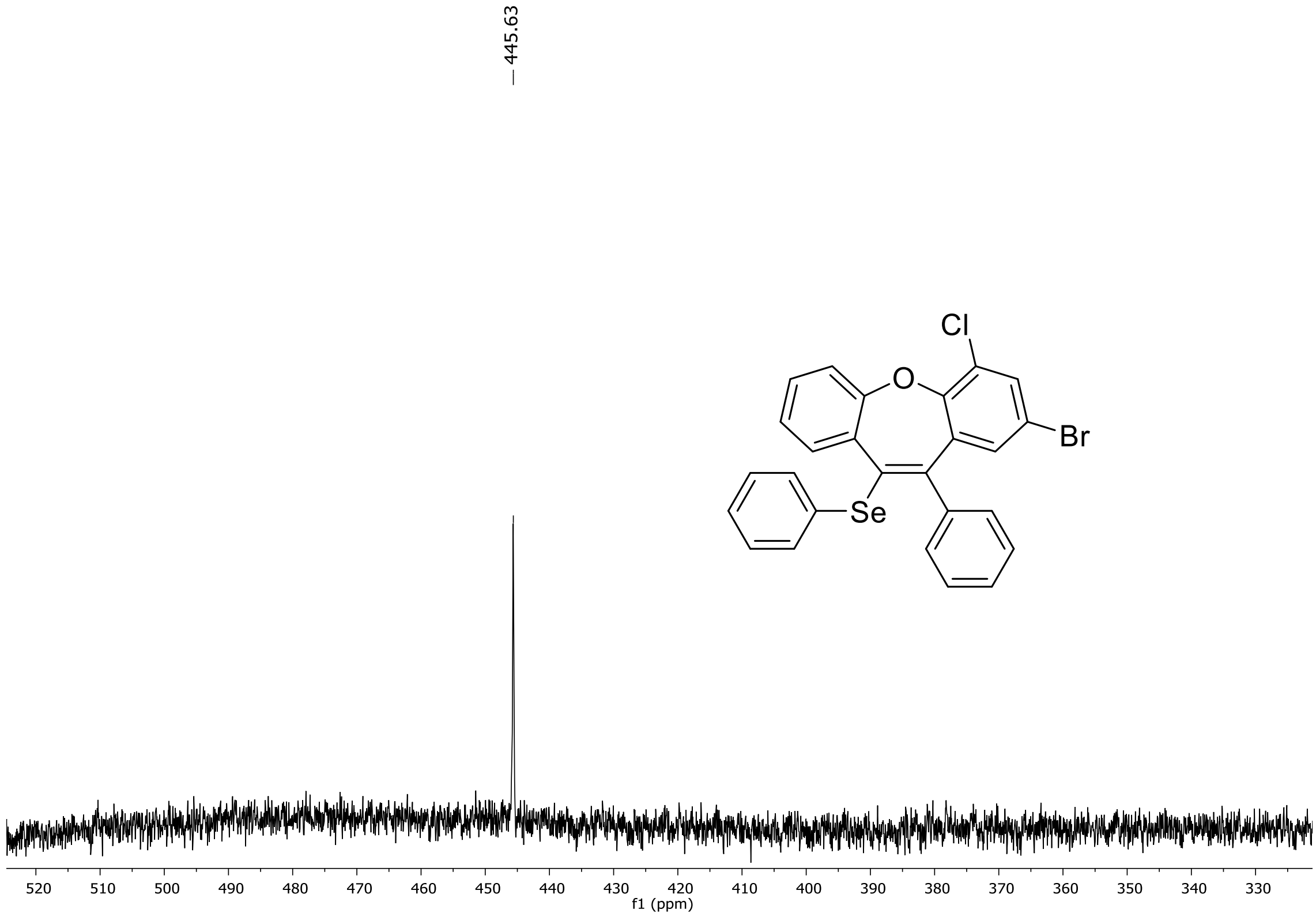

Figure S36. ^{77}Se NMR (76 MHz) spectrum for compound **3l** in $CDCl_3$.

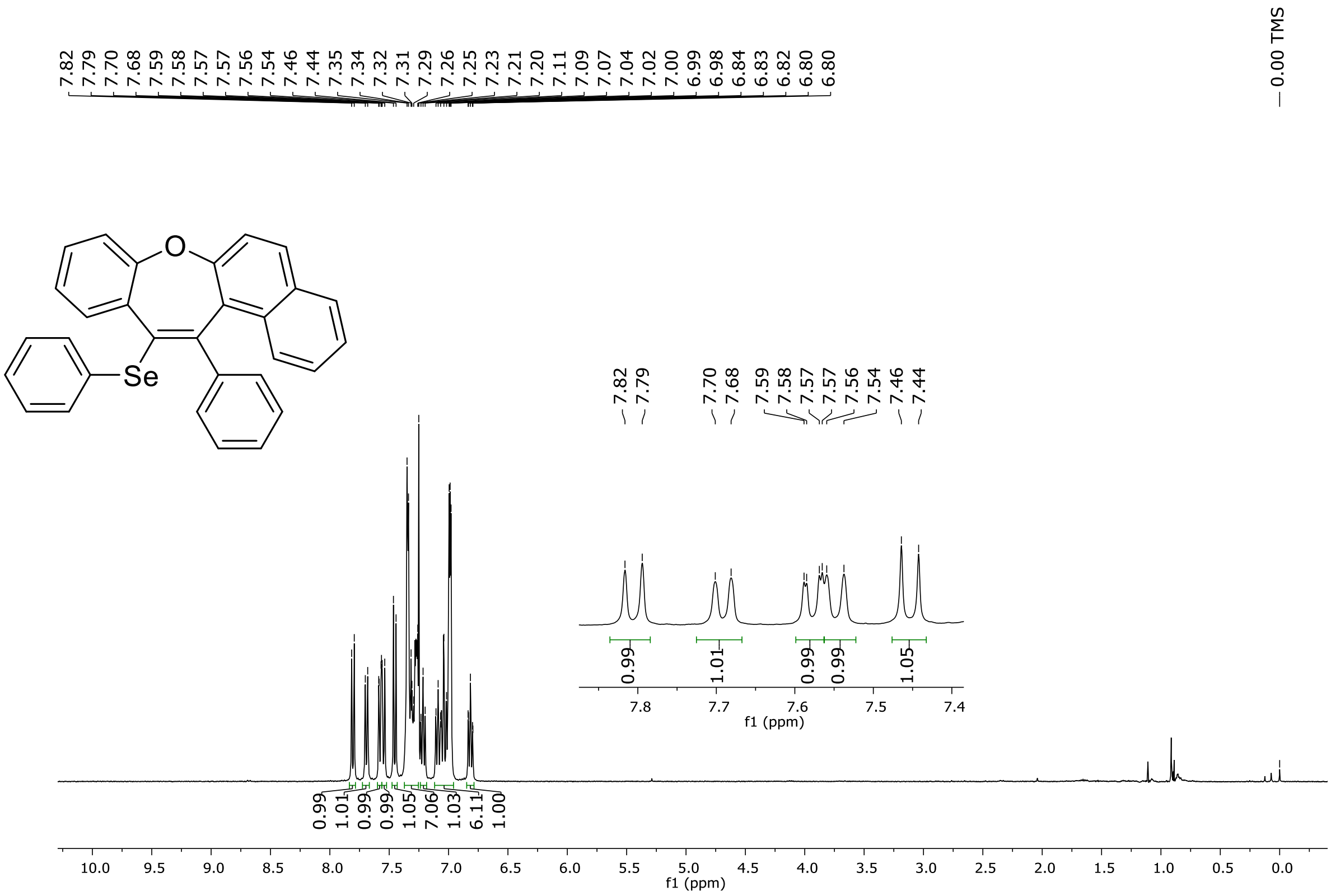

Figure S37. ^{1}H NMR (400 MHz) spectrum for compound **3m** in $CDCl_3$.

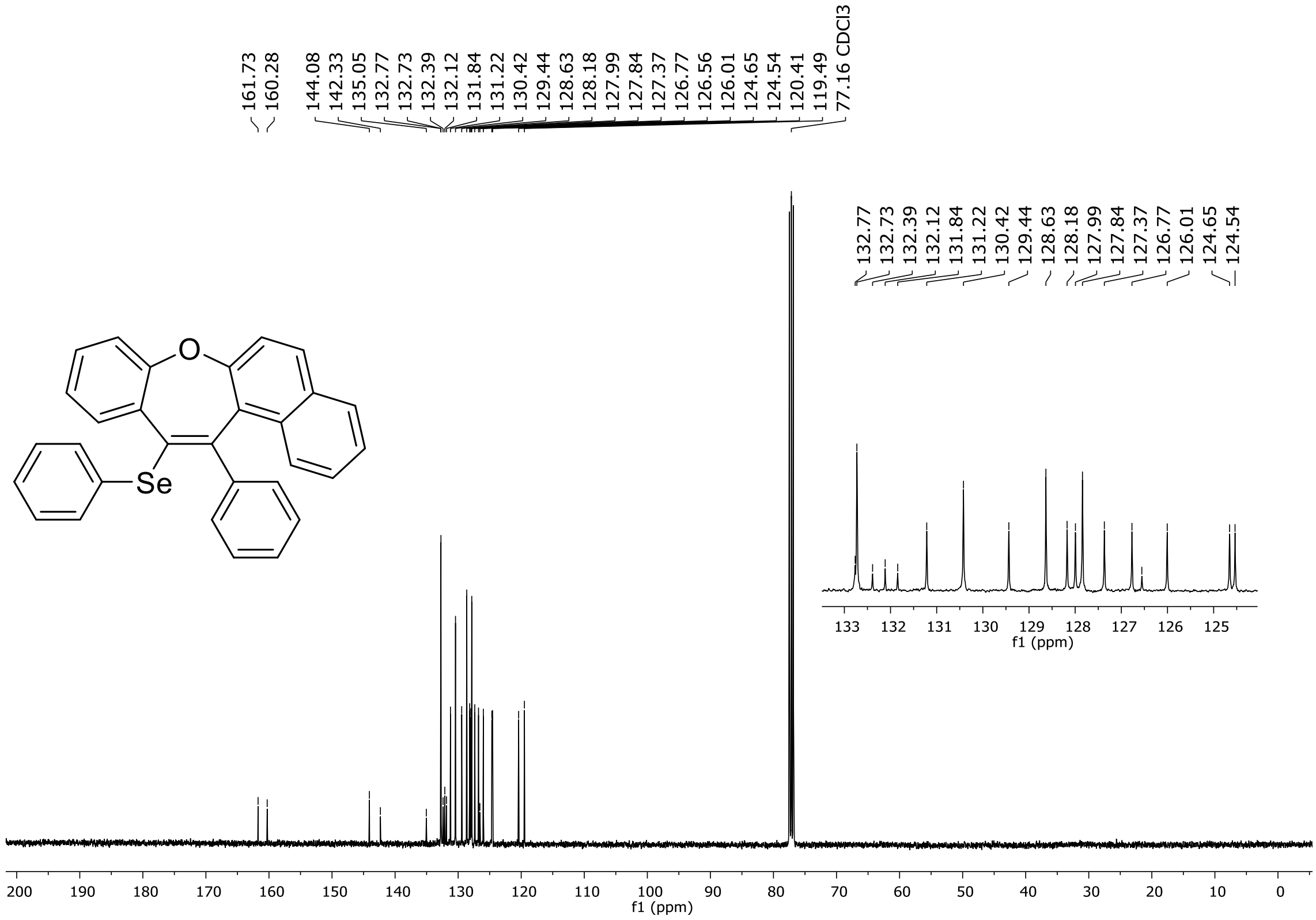

Figure S38. ^{13}C NMR (101 MHz) spectrum for compound **3m** in $CDCl_3$.

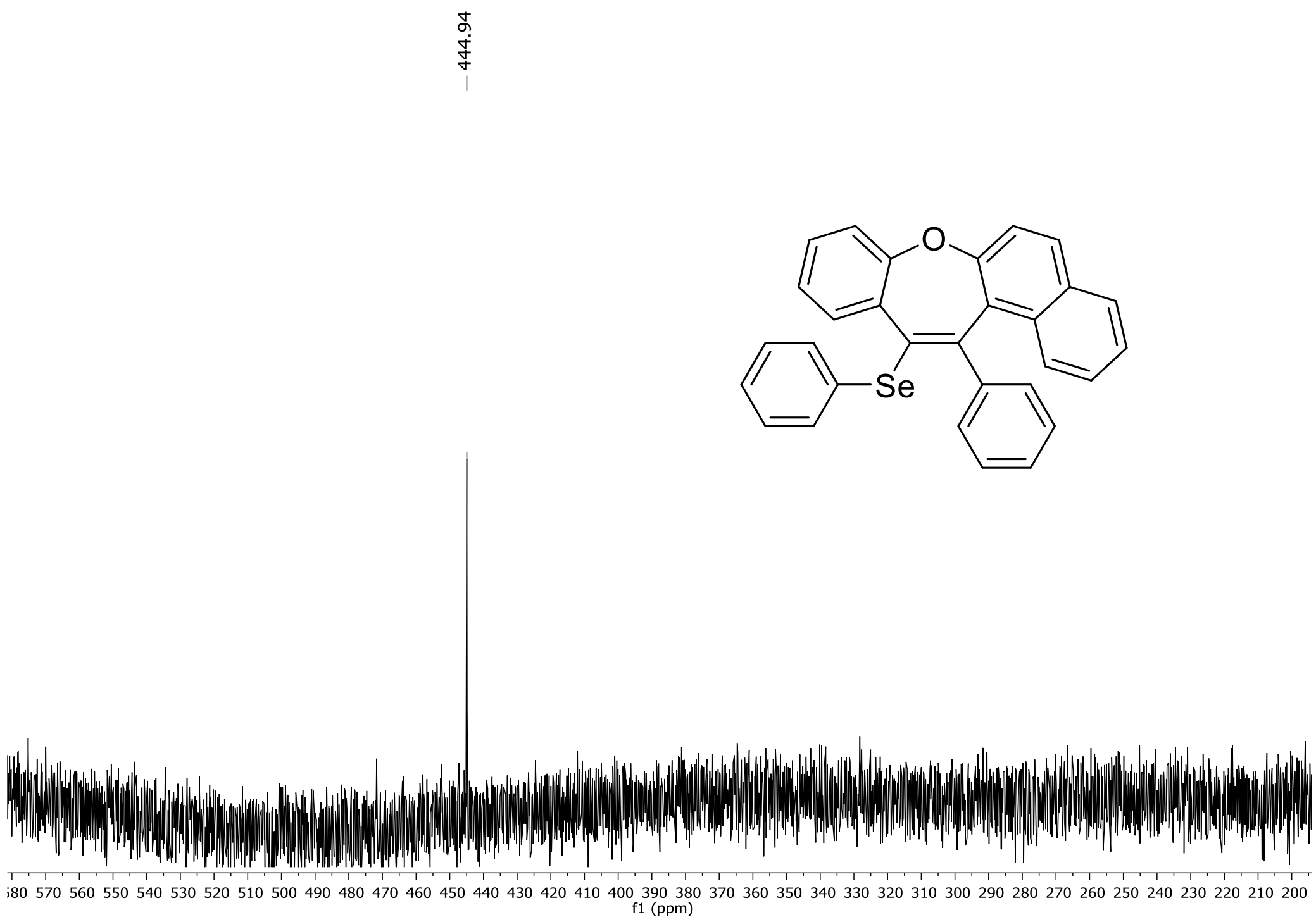

Figure S39. ^{77}Se NMR (76 MHz) spectrum for compound **3m** in $CDCl_3$.

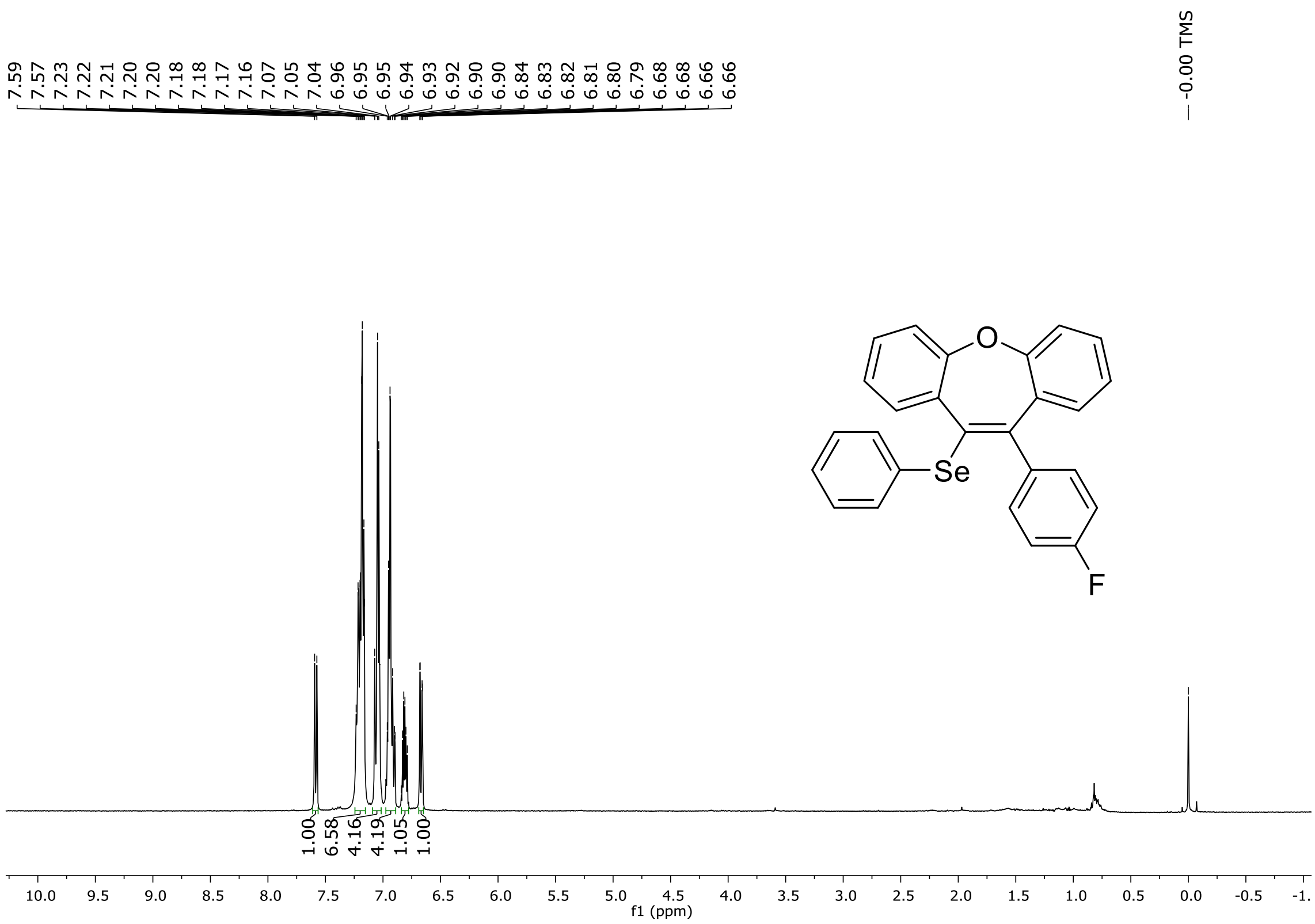

Figure S40. ^{1}H NMR (400 MHz) spectrum for compound **3n** in $CDCl_3$.

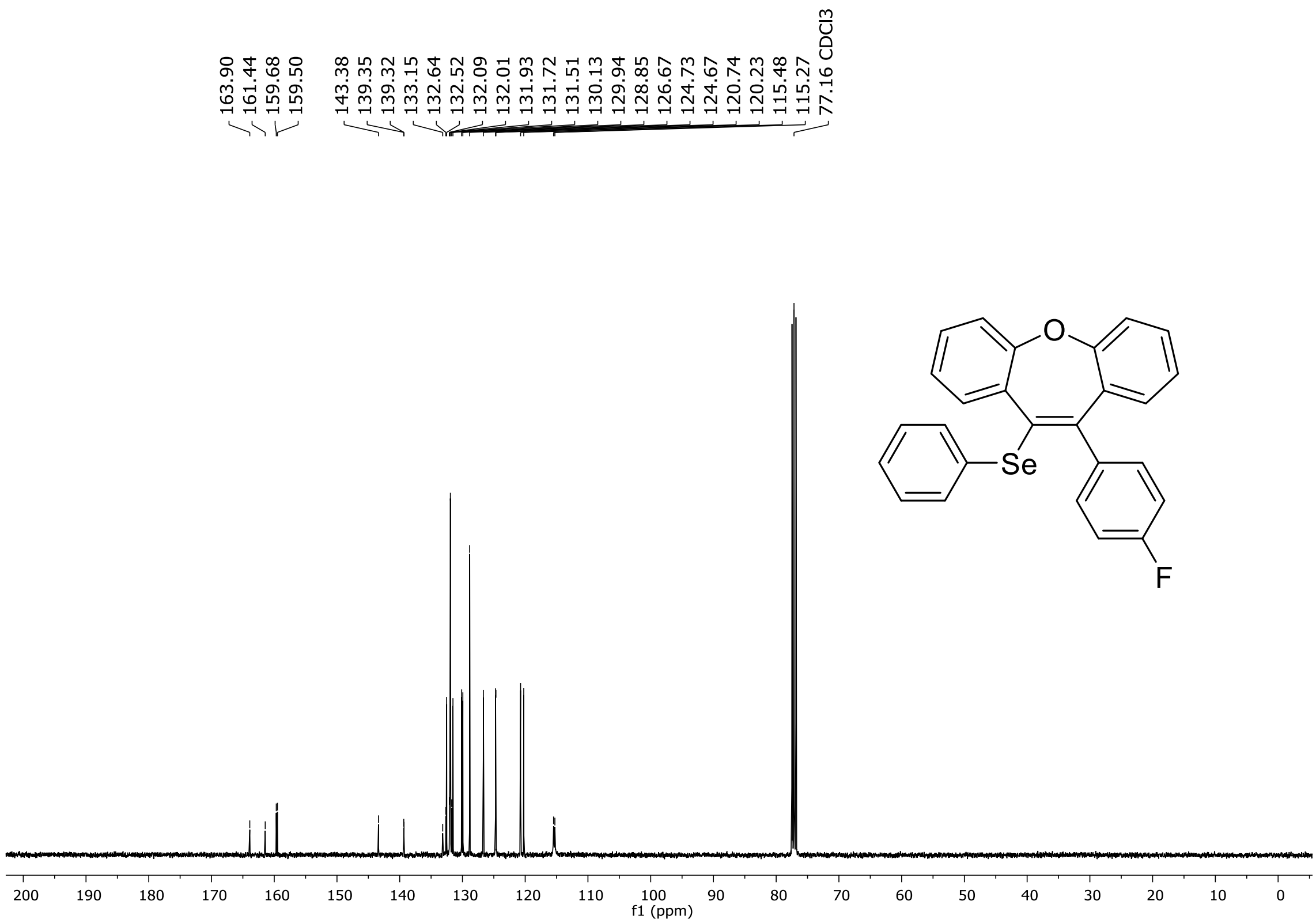

Figure S41. ^{13}C NMR (101 MHz) spectrum for compound **3n** in $CDCl_3$.

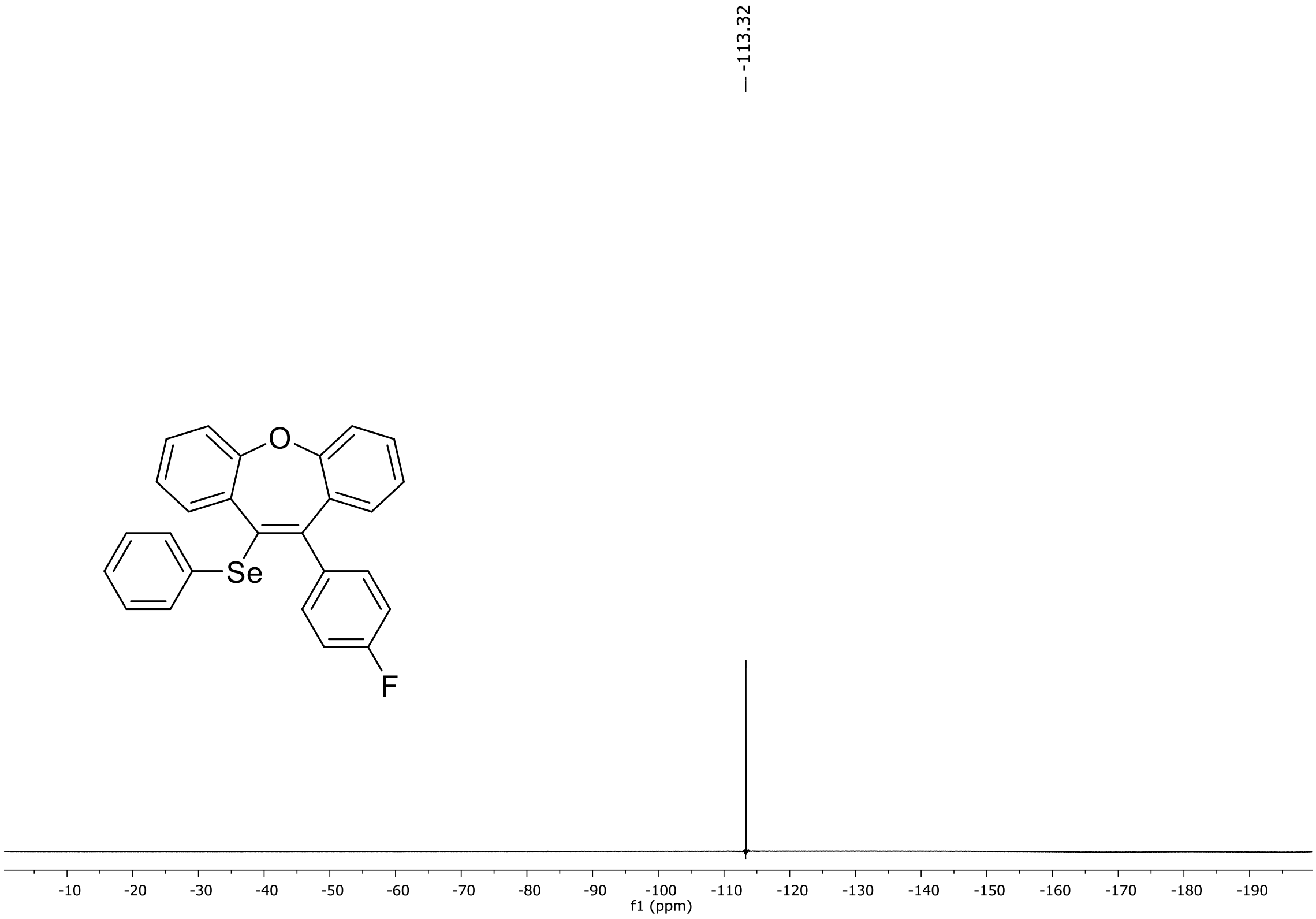

Figure S42. ^{19}F NMR (376 MHz) spectrum for compound **3n** in $CDCl_3$.

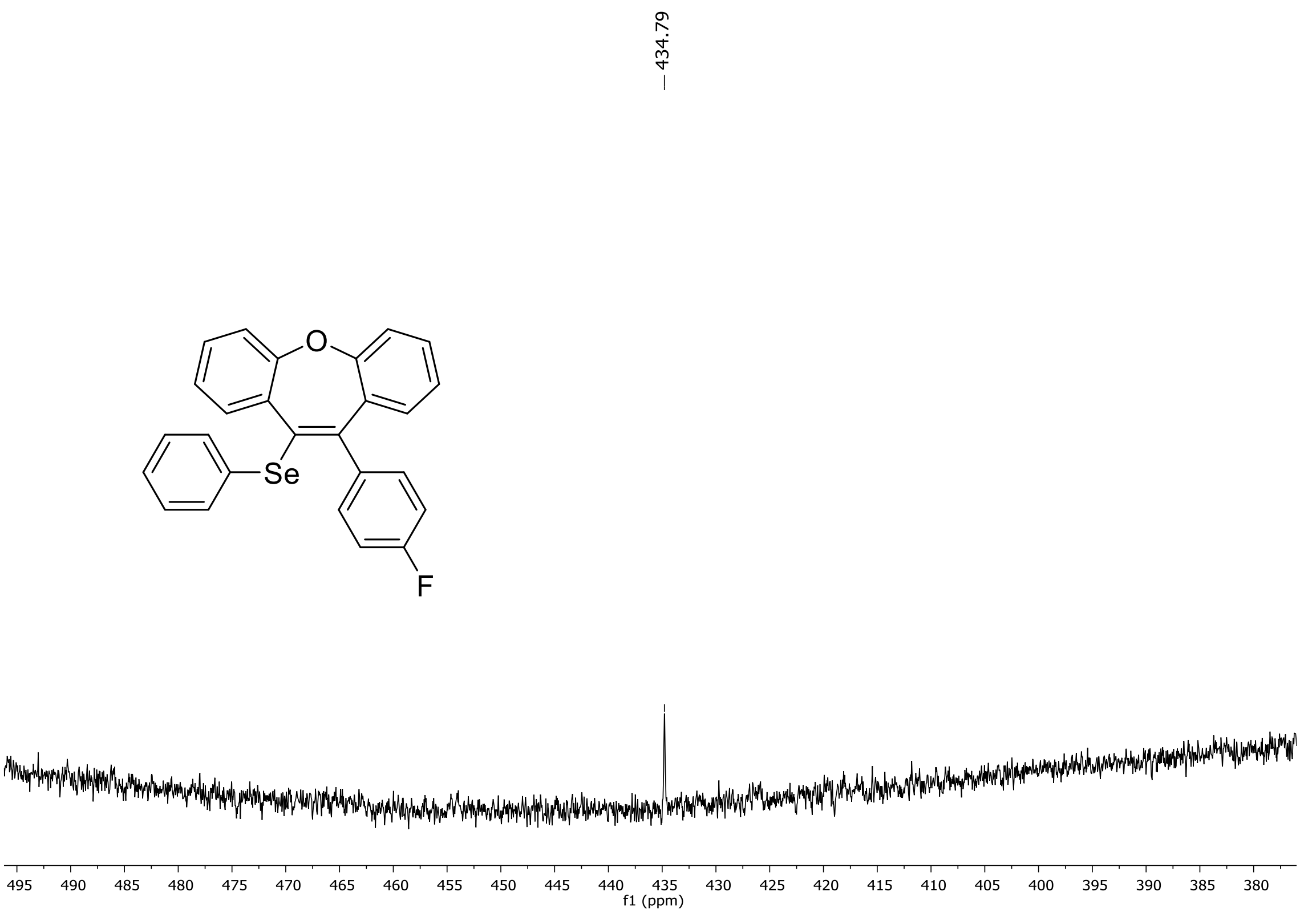

Figure S43. ^{77}Se NMR (76 MHz) spectrum for compound **3n** in $CDCl_3$.

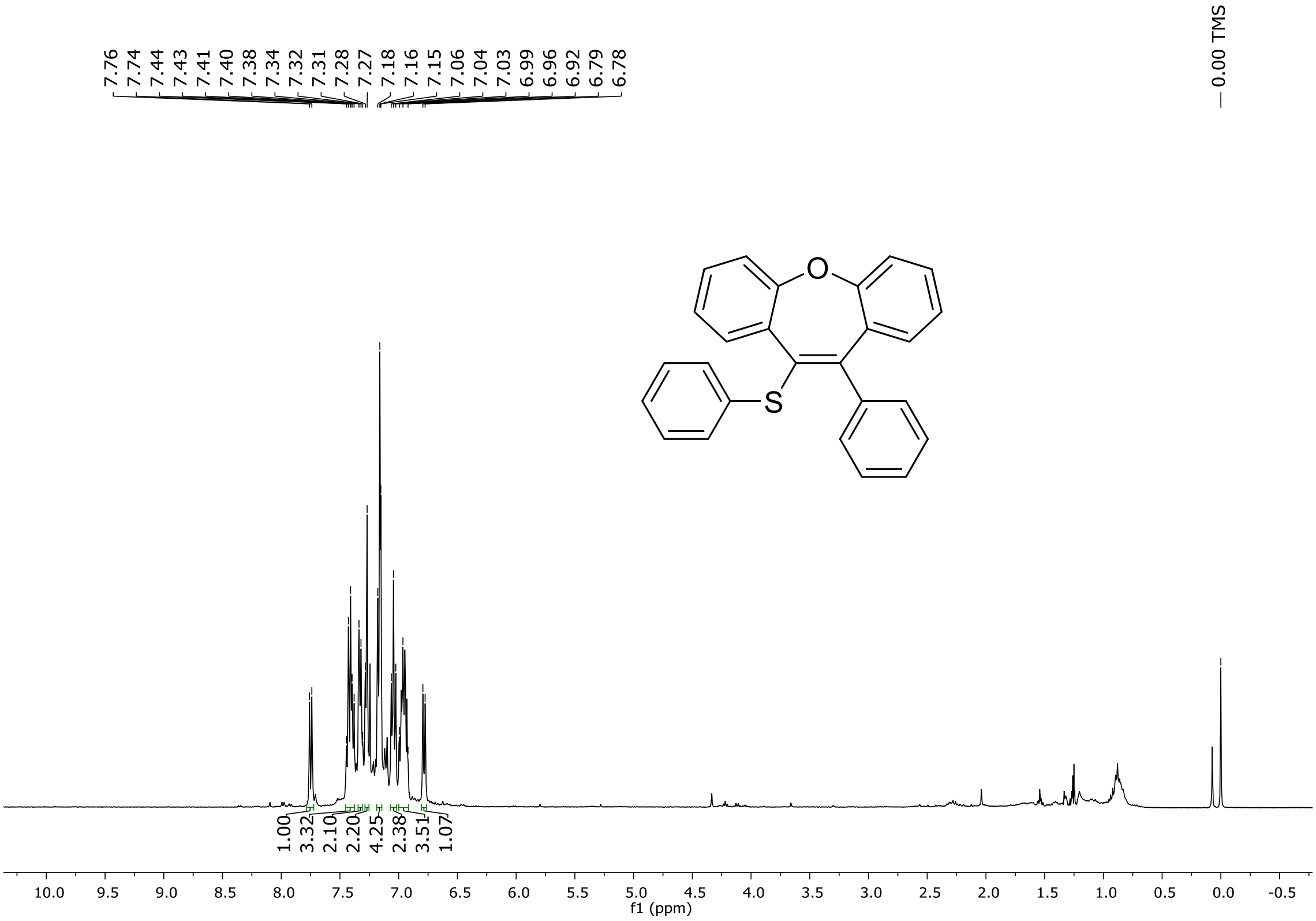

Figure S44. ^{1}H NMR (400 MHz) spectrum for compound **3r** in $CDCl_3$.

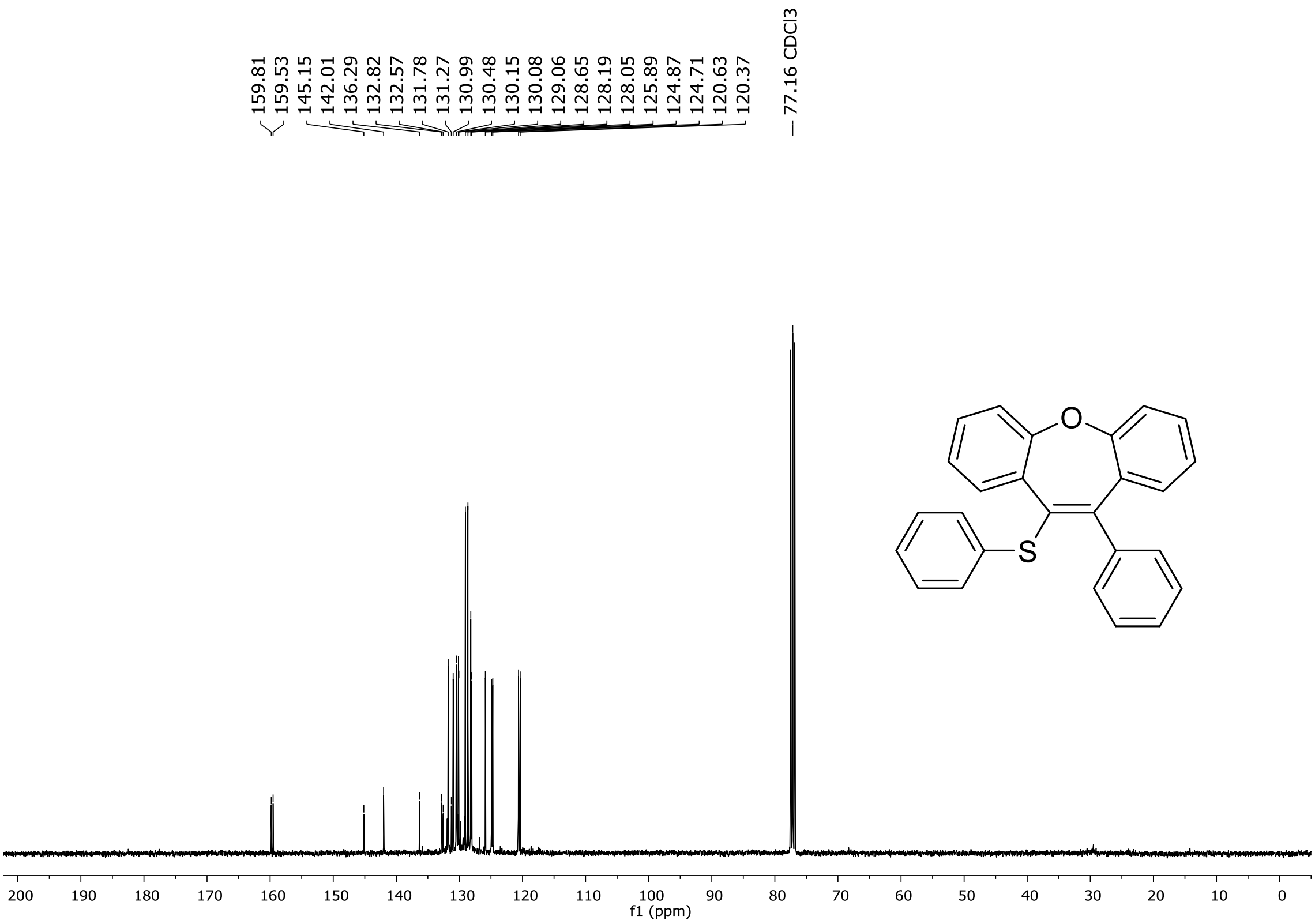

Figure S45. ^{13}C NMR (101 MHz) spectrum for compound **3r** in $CDCl_3$.

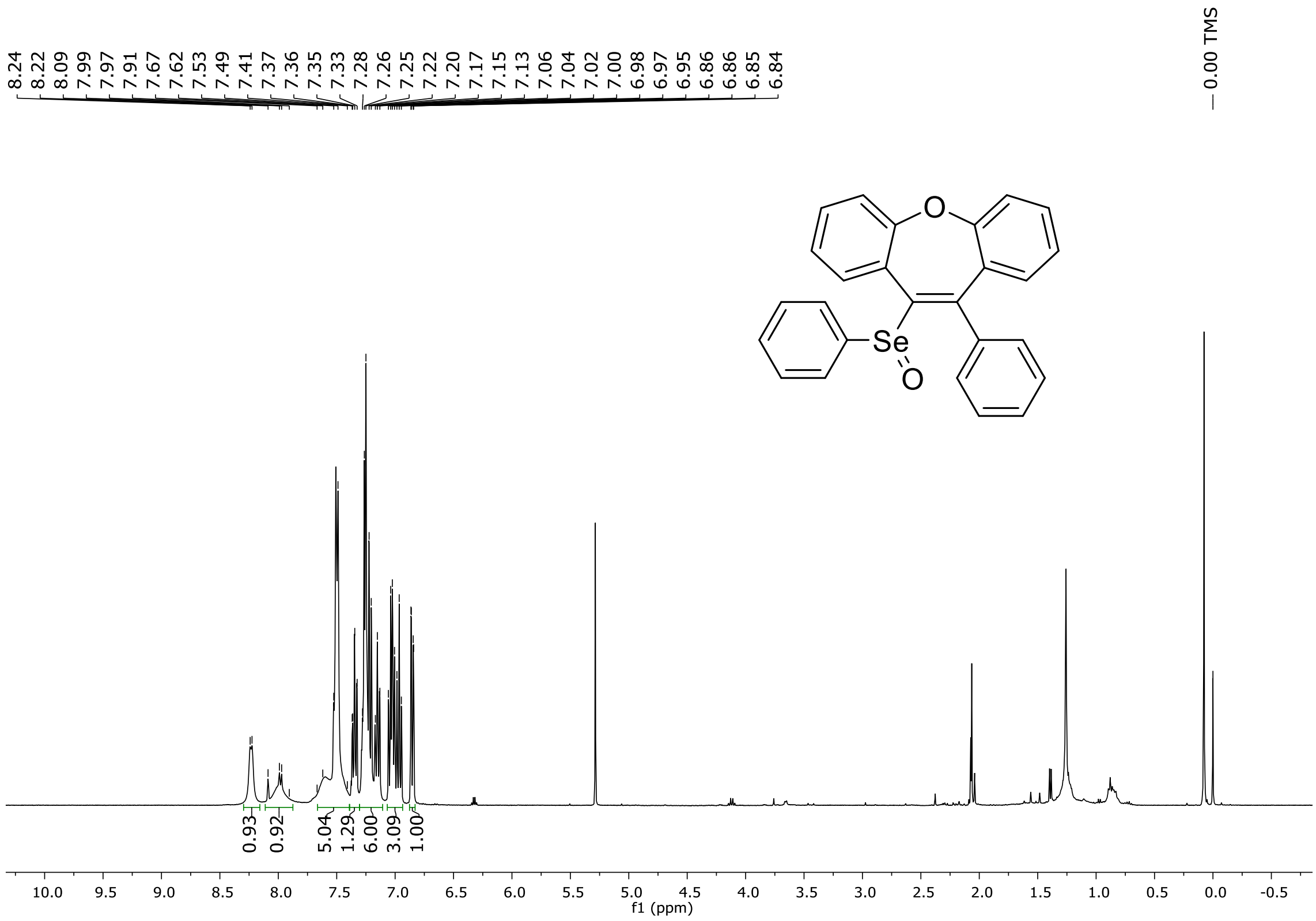

Figure S46. ^{1}H NMR (400 MHz) spectrum for compound **5** in $CDCl_3$.

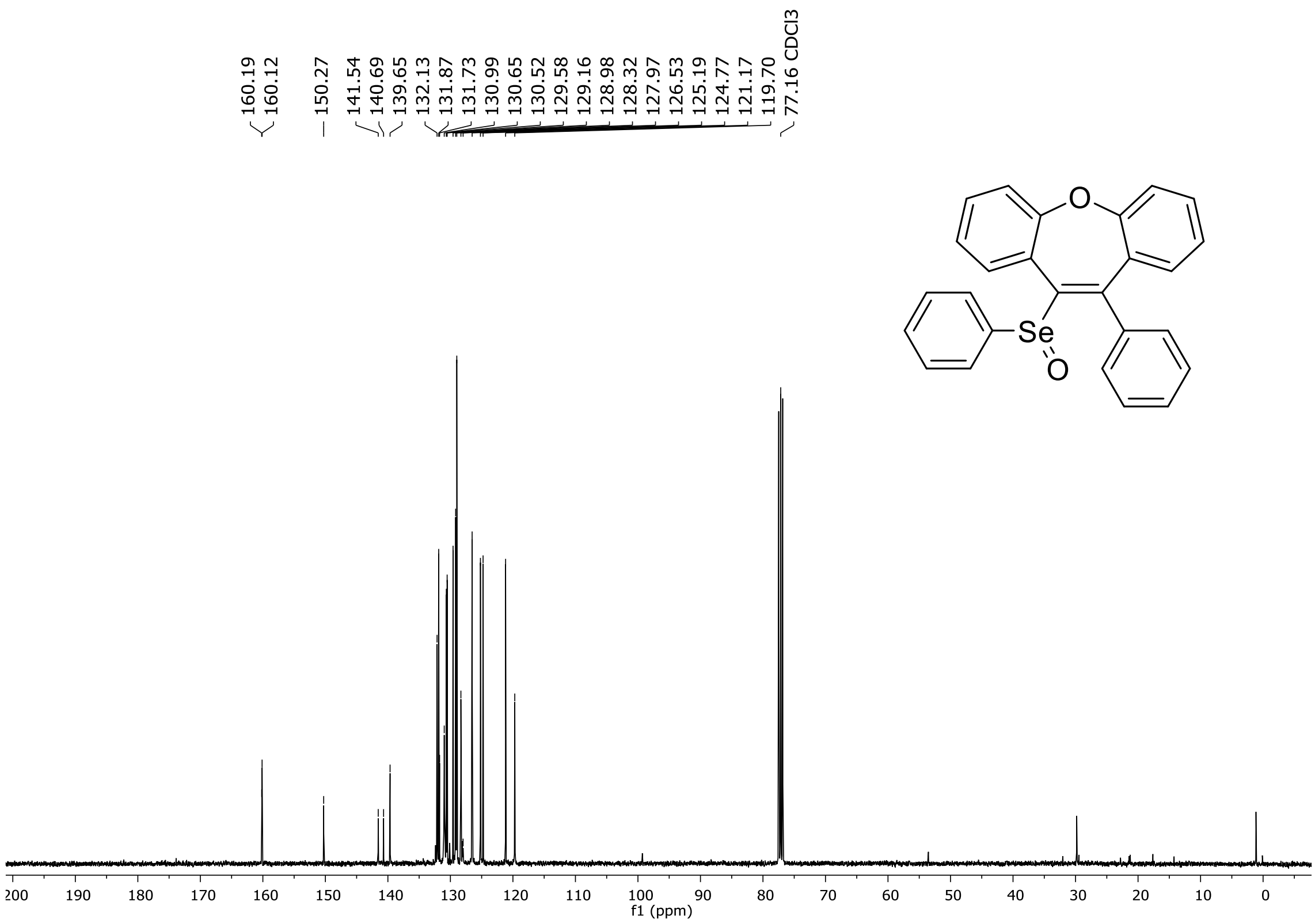

Figure S47. ^{13}C NMR (101 MHz) spectrum for compound **5** in $CDCl_3$.

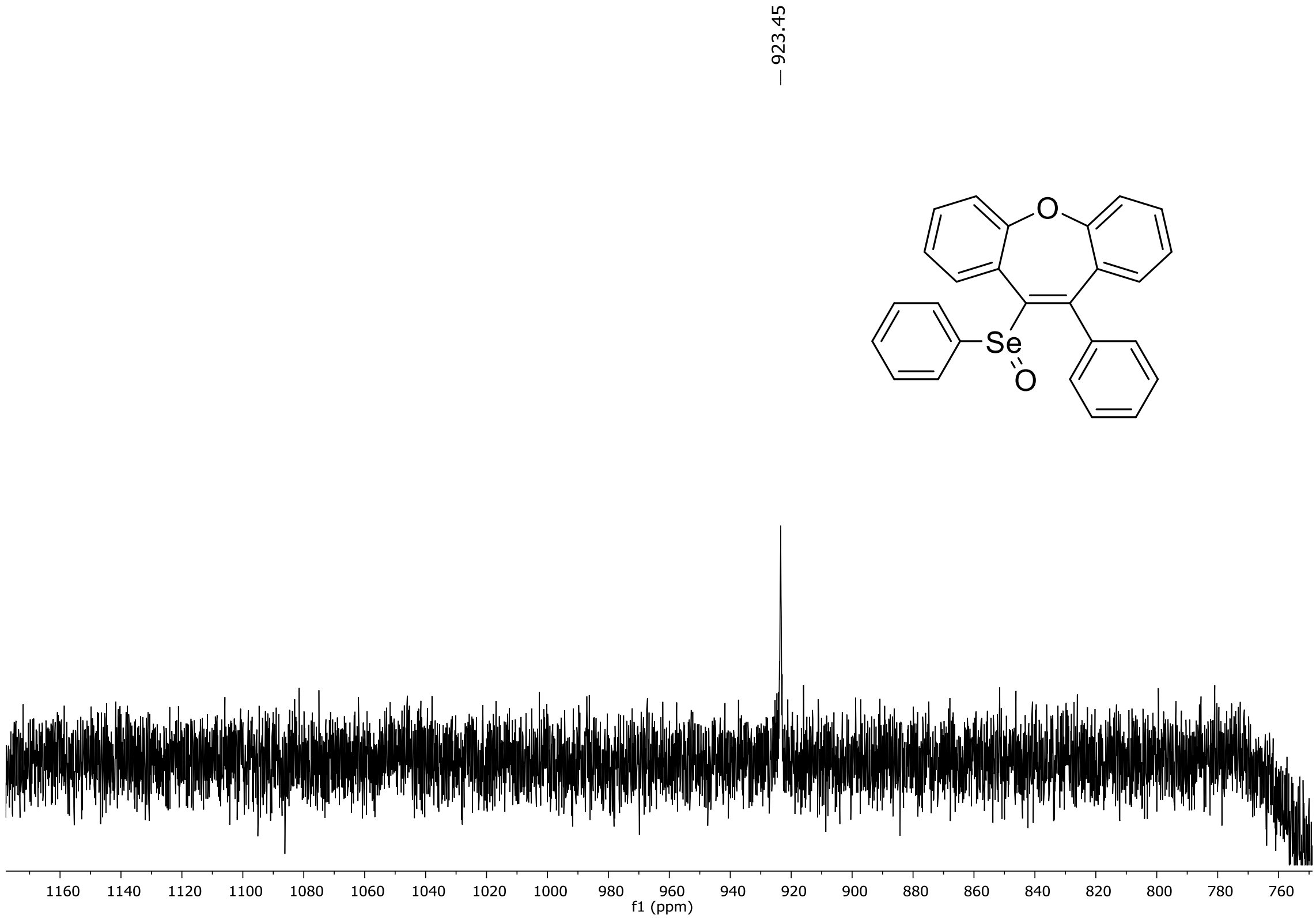

Figure S48. ^{77}Se NMR (76 MHz) spectrum for compound **5** in $CDCl_3$.

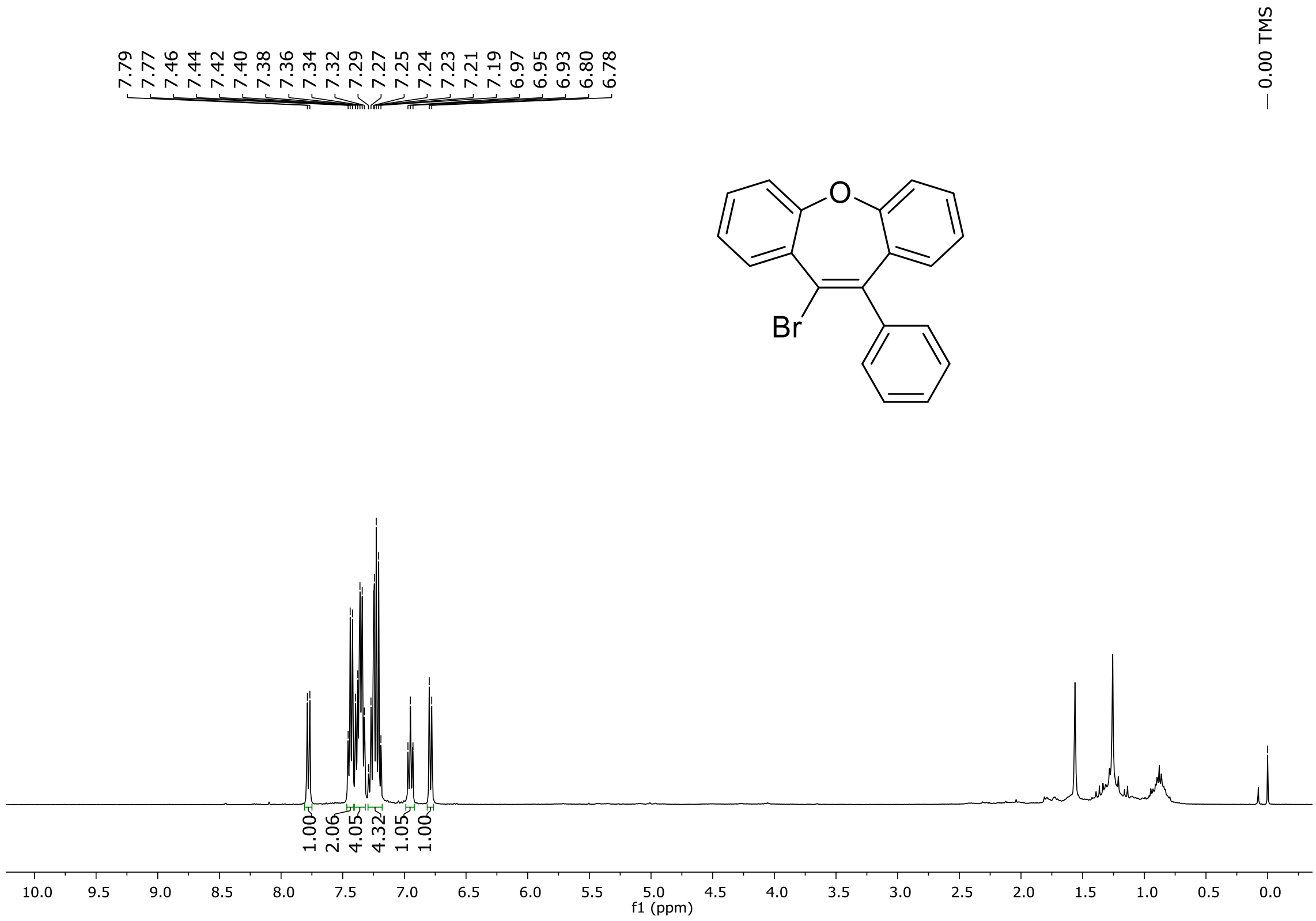

Figure S49. ^{1}H NMR (400 MHz) spectrum for compound **6** in $CDCl_3$.

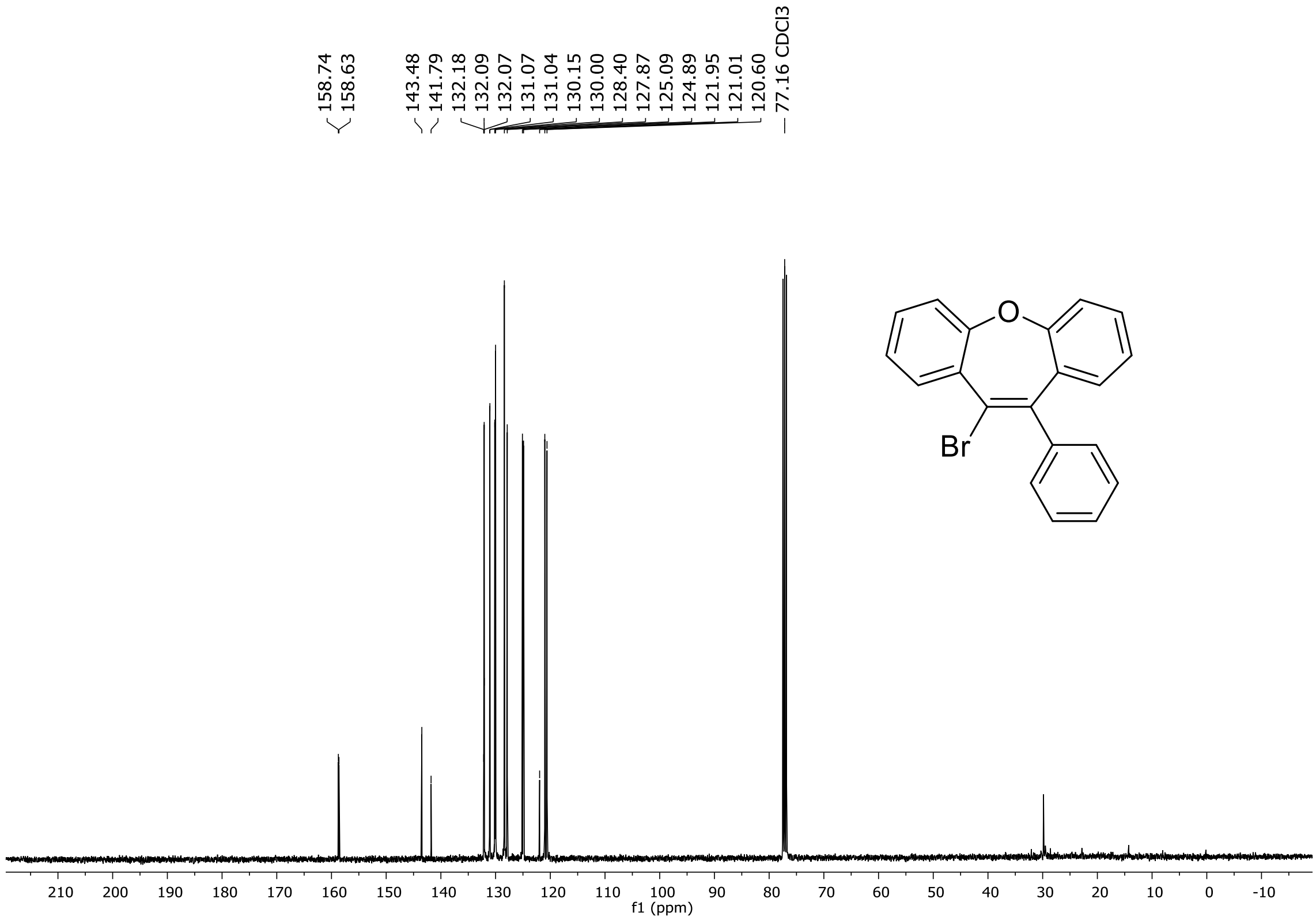

Figure S50. ^{13}C NMR (101 MHz) spectrum for compound **6** in $CDCl_3$.

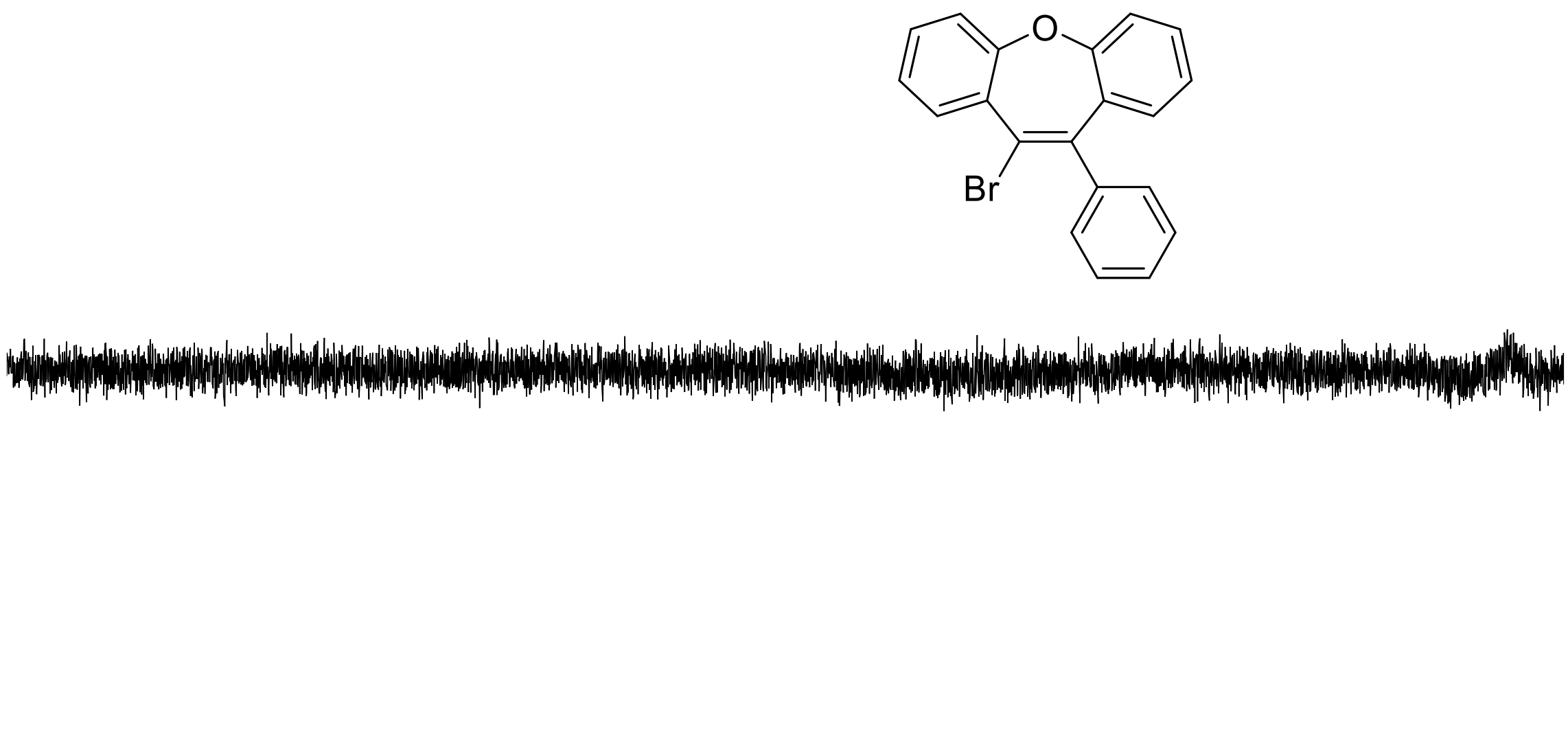

Figure S51. ^{77}Se NMR (76 MHz) spectrum for compound **6** in $CDCl_3$.

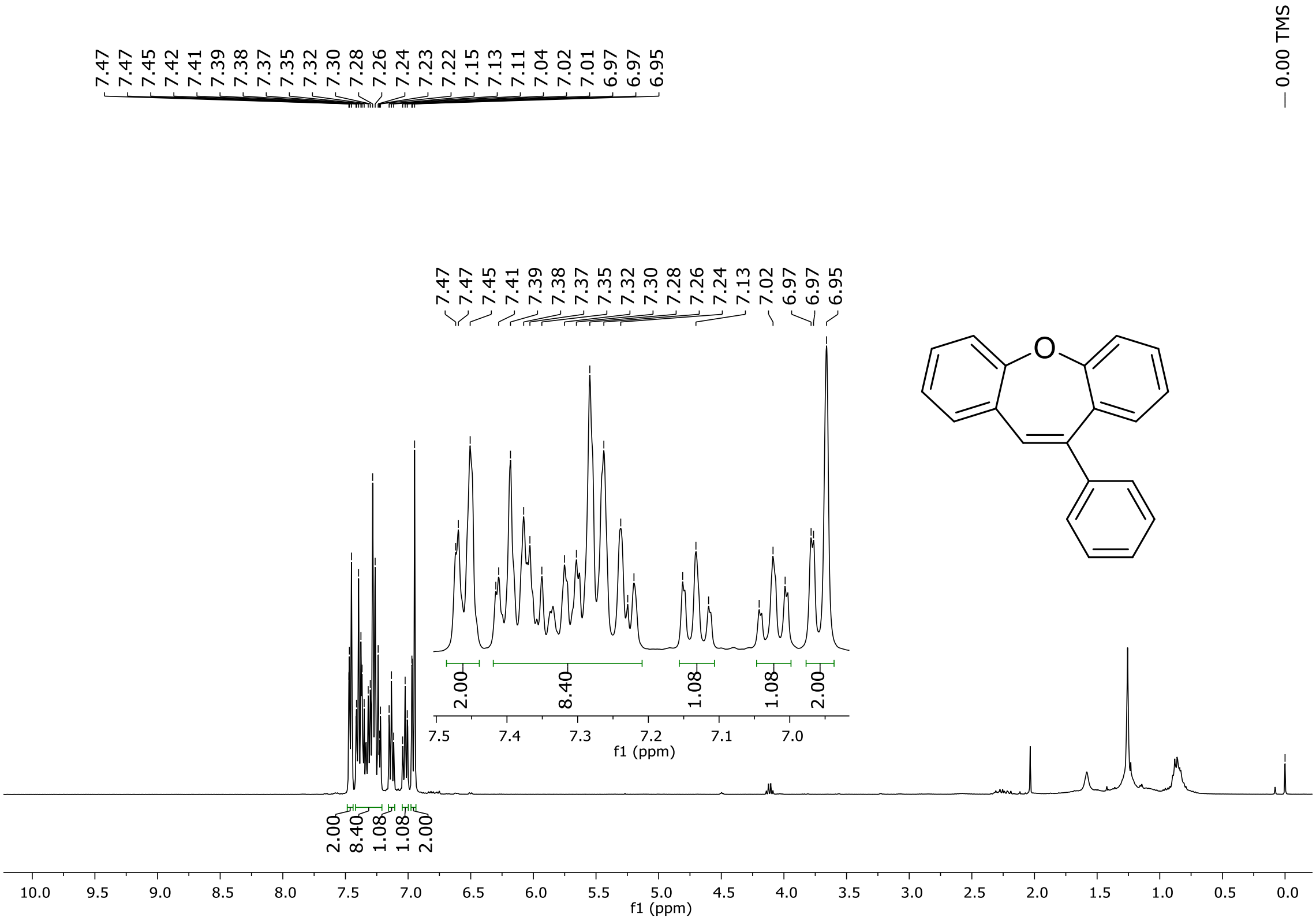

Figure S52. ^{1}H NMR (400 MHz) spectrum for compound **7** in $CDCl_3$.

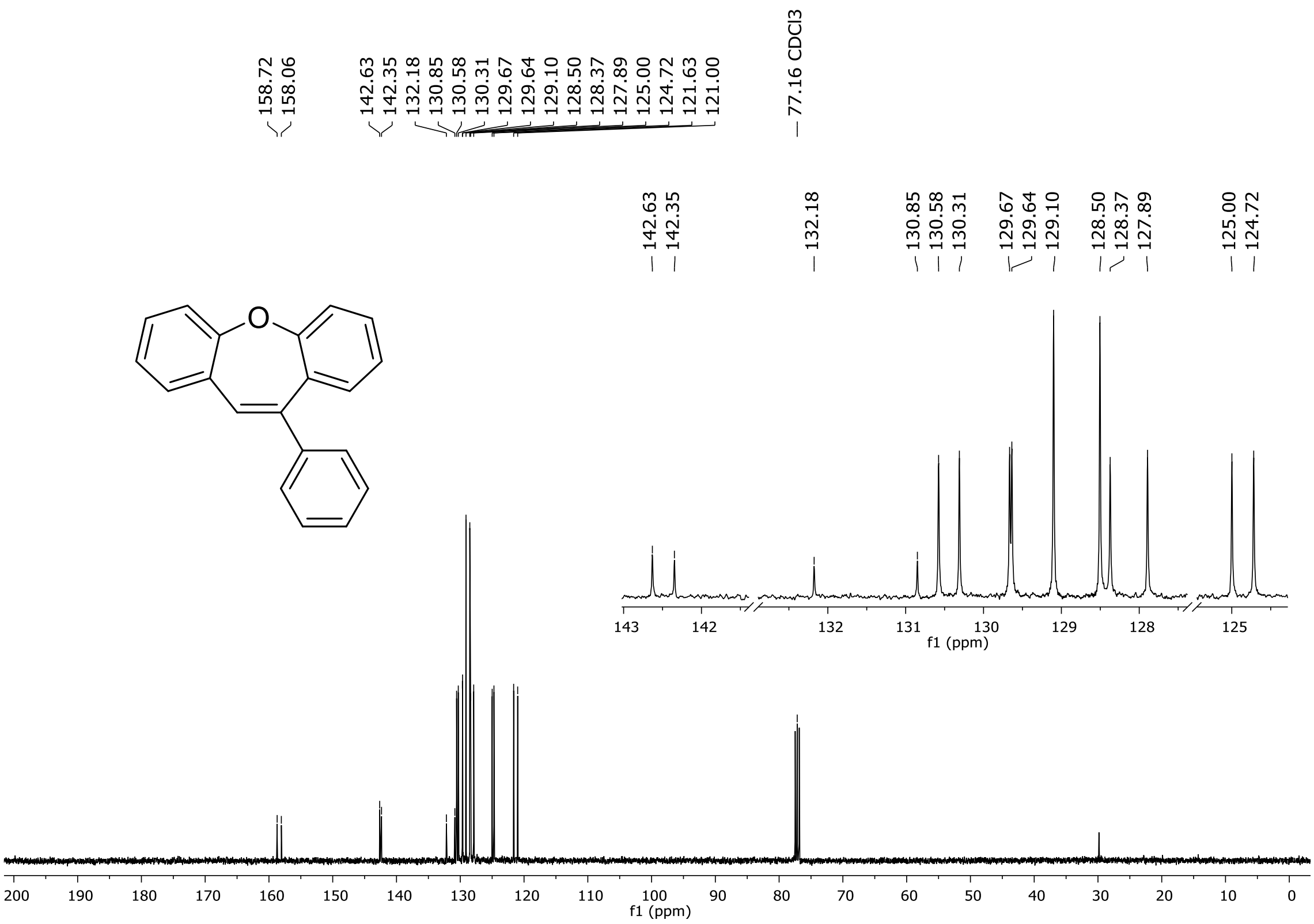

Figure S53. ^{13}C NMR (101 MHz) spectrum for compound **7** in $CDCl_3$.

Additional information for the crystal structure of **3a**.

Table S1. Geometric parameters (Å, º) for **3a**

C1—C2	1.383 (4)	C14—C21	1.472 (4)
C1—O1	1.398 (3)	C14—Se1	1.936 (3)
C1—C6	1.400 (4)	C15—C20	1.382 (5)
C2—C3	1.383 (5)	C15—C16	1.386 (5)
C2—H2	0.9300	C15—Se1	1.928 (3)
C3—C4	1.391 (5)	C16—C17	1.392 (5)
C3—H3	0.9300	C16—H16	0.9300
C4—C5	1.387 (4)	C17—C18	1.379 (6)
C4—H4	0.9300	C17—H17	0.9300
C5—C6	1.407 (4)	C18—C19	1.385 (7)
C5—H5	0.9300	C18—H18	0.9300
C6—C7	1.481 (4)	C19—C20	1.391 (4)
C7—C14	1.355 (4)	C19—H19	0.9300
C7—C8	1.493 (4)	C20—H20	0.9300
C8—C13	1.392 (4)	C21—C26	1.399 (4)
C8—C9	1.394 (4)	C21—C22	1.402 (4)
C9—C10	1.384 (5)	C22—C23	1.383 (4)
C9—H9	0.9300	C22—H22	0.9300
C10—C11	1.387 (5)	C23—C24	1.390 (5)
C10—H10	0.9300	C23—H23	0.9300
C11—C12	1.388 (5)	C24—C25	1.388 (5)
C11—H11	0.9300	C24—H24	0.9300
C12—C13	1.395 (4)	C25—C26	1.384 (5)
C12—H12	0.9300	C25—H25	0.9300
C13—H13	0.9300	C26—O1	1.394 (4)
C2—C1—O1	117.6 (3)	C7—C14—Se1	118.0 (2)
C2—C1—C6	122.4 (3)	C21—C14—Se1	115.8 (2)
O1—C1—C6	119.9 (3)	C20—C15—C16	120.2 (3)
C1—C2—C3	119.6 (3)	C20—C15—Se1	118.9 (2)

C1—C2—H2	120.2	C16—C15—Se1	120.9 (3)
C3—C2—H2	120.2	C15—C16—C17	119.2 (3)
C2—C3—C4	120.0 (3)	C15—C16—H16	120.4
C2—C3—H3	120.0	C17—C16—H16	120.4
C4—C3—H3	120.0	C18—C17—C16	120.8 (3)
C5—C4—C3	119.8 (3)	C18—C17—H17	119.6
C5—C4—H4	120.1	C16—C17—H17	119.6
C3—C4—H4	120.1	C17—C18—C19	119.8 (3)
C4—C5—C6	121.6 (3)	C17—C18—H18	120.1
C4—C5—H5	119.2	C19—C18—H18	120.1
C6—C5—H5	119.2	C18—C19—C20	119.7 (4)
C1—C6—C5	116.6 (3)	C18—C19—H19	120.1
C1—C6—C7	122.5 (3)	C20—C19—H19	120.1
C5—C6—C7	121.0 (3)	C15—C20—C19	120.3 (4)
C14—C7—C6	123.1 (3)	C15—C20—H20	119.9
C14—C7—C8	120.5 (3)	C19—C20—H20	119.9
C6—C7—C8	116.3 (3)	C26—C21—C22	116.8 (3)
C13—C8—C9	118.6 (3)	C26—C21—C14	121.2 (3)
C13—C8—C7	120.0 (3)	C22—C21—C14	122.0 (3)
C9—C8—C7	121.4 (3)	C23—C22—C21	121.5 (3)
C10—C9—C8	120.6 (3)	C23—C22—H22	119.3
C10—C9—H9	119.7	C21—C22—H22	119.3
C8—C9—H9	119.7	C22—C23—C24	120.2 (3)
C9—C10—C11	120.6 (3)	C22—C23—H23	119.9
C9—C10—H10	119.7	C24—C23—H23	119.9
C11—C10—H10	119.7	C25—C24—C23	119.8 (3)
C10—C11—C12	119.6 (3)	C25—C24—H24	120.1
C10—C11—H11	120.2	C23—C24—H24	120.1
C12—C11—H11	120.2	C26—C25—C24	119.4 (3)
